# Precision measurement and interpretation of inclusive $W^+$, $W^-$ and $Z/\gamma ^*$ production cross sections with the ATLAS detector

**DOI:** 10.1140/epjc/s10052-017-4911-9

**Published:** 2017-06-02

**Authors:** M. Aaboud, G. Aad, B. Abbott, J. Abdallah, O. Abdinov, B. Abeloos, O. S. AbouZeid, N. L. Abraham, H. Abramowicz, H. Abreu, R. Abreu, Y. Abulaiti, B. S. Acharya, S. Adachi, L. Adamczyk, D. L. Adams, J. Adelman, S. Adomeit, T. Adye, A. A. Affolder, T. Agatonovic-Jovin, J. A. Aguilar-Saavedra, S. P. Ahlen, F. Ahmadov, G. Aielli, H. Akerstedt, T. P. A. Åkesson, A. V. Akimov, G. L. Alberghi, J. Albert, S. Albrand, M. J. Alconada Verzini, M. Aleksa, I. N. Aleksandrov, C. Alexa, G. Alexander, T. Alexopoulos, M. Alhroob, B. Ali, M. Aliev, G. Alimonti, J. Alison, S. P. Alkire, B. M. M. Allbrooke, B. W. Allen, P. P. Allport, A. Aloisio, A. Alonso, F. Alonso, C. Alpigiani, A. A. Alshehri, M. Alstaty, B. Alvarez Gonzalez, D. Álvarez Piqueras, M. G. Alviggi, B. T. Amadio, Y. Amaral Coutinho, C. Amelung, D. Amidei, S. P. Amor Dos Santos, A. Amorim, S. Amoroso, G. Amundsen, C. Anastopoulos, L. S. Ancu, N. Andari, T. Andeen, C. F. Anders, J. K. Anders, K. J. Anderson, A. Andreazza, V. Andrei, S. Angelidakis, I. Angelozzi, A. Angerami, F. Anghinolfi, A. V. Anisenkov, N. Anjos, A. Annovi, C. Antel, M. Antonelli, A. Antonov, D. J. Antrim, F. Anulli, M. Aoki, L. Aperio Bella, G. Arabidze, Y. Arai, J. P. Araque, V. Araujo Ferraz, A. T. H. Arce, F. A. Arduh, J.-F. Arguin, S. Argyropoulos, M. Arik, A. J. Armbruster, L. J. Armitage, O. Arnaez, H. Arnold, M. Arratia, O. Arslan, A. Artamonov, G. Artoni, S. Artz, S. Asai, N. Asbah, A. Ashkenazi, B. Åsman, L. Asquith, K. Assamagan, R. Astalos, M. Atkinson, N. B. Atlay, K. Augsten, G. Avolio, B. Axen, M. K. Ayoub, G. Azuelos, M. A. Baak, A. E. Baas, M. J. Baca, H. Bachacou, K. Bachas, M. Backes, M. Backhaus, P. Bagiacchi, P. Bagnaia, Y. Bai, J. T. Baines, M. Bajic, O. K. Baker, E. M. Baldin, P. Balek, T. Balestri, F. Balli, W. K. Balunas, E. Banas, Sw. Banerjee, A. A. E. Bannoura, L. Barak, E. L. Barberio, D. Barberis, M. Barbero, T. Barillari, M.-S. Barisits, T. Barklow, N. Barlow, S. L. Barnes, B. M. Barnett, R. M. Barnett, Z. Barnovska-Blenessy, A. Baroncelli, G. Barone, A. J. Barr, L. Barranco Navarro, F. Barreiro, J. Barreiro Guimarães da Costa, R. Bartoldus, A. E. Barton, P. Bartos, A. Basalaev, A. Bassalat, R. L. Bates, S. J. Batista, J. R. Batley, M. Battaglia, M. Bauce, F. Bauer, H. S. Bawa, J. B. Beacham, M. D. Beattie, T. Beau, P. H. Beauchemin, P. Bechtle, H. P. Beck, K. Becker, M. Becker, M. Beckingham, C. Becot, A. J. Beddall, A. Beddall, V. A. Bednyakov, M. Bedognetti, C. P. Bee, L. J. Beemster, T. A. Beermann, M. Begel, J. K. Behr, A. S. Bell, G. Bella, L. Bellagamba, A. Bellerive, M. Bellomo, K. Belotskiy, O. Beltramello, N. L. Belyaev, O. Benary, D. Benchekroun, M. Bender, K. Bendtz, N. Benekos, Y. Benhammou, E. Benhar Noccioli, J. Benitez, D. P. Benjamin, J. R. Bensinger, S. Bentvelsen, L. Beresford, M. Beretta, D. Berge, E. Bergeaas Kuutmann, N. Berger, J. Beringer, S. Berlendis, N. R. Bernard, C. Bernius, F. U. Bernlochner, T. Berry, P. Berta, C. Bertella, G. Bertoli, F. Bertolucci, I. A. Bertram, C. Bertsche, D. Bertsche, G. J. Besjes, O. Bessidskaia Bylund, M. Bessner, N. Besson, C. Betancourt, A. Bethani, S. Bethke, A. J. Bevan, R. M. Bianchi, M. Bianco, O. Biebel, D. Biedermann, R. Bielski, N. V. Biesuz, M. Biglietti, J. Bilbao De Mendizabal, T. R. V. Billoud, H. Bilokon, M. Bindi, A. Bingul, C. Bini, S. Biondi, T. Bisanz, D. M. Bjergaard, C. W. Black, J. E. Black, K. M. Black, D. Blackburn, R. E. Blair, T. Blazek, I. Bloch, C. Blocker, A. Blue, W. Blum, U. Blumenschein, S. Blunier, G. J. Bobbink, V. S. Bobrovnikov, S. S. Bocchetta, A. Bocci, C. Bock, M. Boehler, D. Boerner, J. A. Bogaerts, D. Bogavac, A. G. Bogdanchikov, C. Bohm, V. Boisvert, P. Bokan, T. Bold, A. S. Boldyrev, M. Bomben, M. Bona, M. Boonekamp, A. Borisov, G. Borissov, J. Bortfeldt, D. Bortoletto, V. Bortolotto, K. Bos, D. Boscherini, M. Bosman, J. D. Bossio Sola, J. Boudreau, J. Bouffard, E. V. Bouhova-Thacker, D. Boumediene, C. Bourdarios, S. K. Boutle, A. Boveia, J. Boyd, I. R. Boyko, J. Bracinik, A. Brandt, G. Brandt, O. Brandt, U. Bratzler, B. Brau, J. E. Brau, W. D. Breaden Madden, K. Brendlinger, A. J. Brennan, L. Brenner, R. Brenner, S. Bressler, T. M. Bristow, D. Britton, D. Britzger, F. M. Brochu, I. Brock, R. Brock, G. Brooijmans, T. Brooks, W. K. Brooks, J. Brosamer, E. Brost, J. H Broughton, P. A. Bruckman de Renstrom, D. Bruncko, R. Bruneliere, A. Bruni, G. Bruni, L. S. Bruni, B. H. Brunt, M. Bruschi, N. Bruscino, P. Bryant, L. Bryngemark, T. Buanes, Q. Buat, P. Buchholz, A. G. Buckley, I. A. Budagov, F. Buehrer, M. K. Bugge, O. Bulekov, D. Bullock, H. Burckhart, S. Burdin, C. D. Burgard, A. M. Burger, B. Burghgrave, K. Burka, S. Burke, I. Burmeister, J. T. P. Burr, E. Busato, D. Büscher, V. Büscher, P. Bussey, J. M. Butler, C. M. Buttar, J. M. Butterworth, P. Butti, W. Buttinger, A. Buzatu, A. R. Buzykaev, S. Cabrera Urbán, D. Caforio, V. M. Cairo, O. Cakir, N. Calace, P. Calafiura, A. Calandri, G. Calderini, P. Calfayan, G. Callea, L. P. Caloba, S. Calvente Lopez, D. Calvet, S. Calvet, T. P. Calvet, R. Camacho Toro, S. Camarda, P. Camarri, D. Cameron, R. Caminal Armadans, C. Camincher, S. Campana, M. Campanelli, A. Camplani, A. Campoverde, V. Canale, A. Canepa, M. Cano Bret, J. Cantero, T. Cao, M. D. M. Capeans Garrido, I. Caprini, M. Caprini, M. Capua, R. M. Carbone, R. Cardarelli, F. Cardillo, I. Carli, T. Carli, G. Carlino, B. T. Carlson, L. Carminati, R. M. D. Carney, S. Caron, E. Carquin, G. D. Carrillo-Montoya, J. R. Carter, J. Carvalho, D. Casadei, M. P. Casado, M. Casolino, D. W. Casper, E. Castaneda-Miranda, R. Castelijn, A. Castelli, V. Castillo Gimenez, N. F. Castro, A. Catinaccio, J. R. Catmore, A. Cattai, J. Caudron, V. Cavaliere, E. Cavallaro, D. Cavalli, M. Cavalli-Sforza, V. Cavasinni, F. Ceradini, L. Cerda Alberich, A. S. Cerqueira, A. Cerri, L. Cerrito, F. Cerutti, A. Cervelli, S. A. Cetin, A. Chafaq, D. Chakraborty, S. K. Chan, Y. L. Chan, P. Chang, J. D. Chapman, D. G. Charlton, A. Chatterjee, C. C. Chau, C. A. Chavez Barajas, S. Che, S. Cheatham, A. Chegwidden, S. Chekanov, S. V. Chekulaev, G. A. Chelkov, M. A. Chelstowska, C. Chen, H. Chen, S. Chen, S. Chen, X. Chen, Y. Chen, H. C. Cheng, H. J. Cheng, Y. Cheng, A. Cheplakov, E. Cheremushkina, R. Cherkaoui El Moursli, V. Chernyatin, E. Cheu, L. Chevalier, V. Chiarella, G. Chiarelli, G. Chiodini, A. S. Chisholm, A. Chitan, Y. H. Chiu, M. V. Chizhov, K. Choi, A. R. Chomont, S. Chouridou, B. K. B. Chow, V. Christodoulou, D. Chromek-Burckhart, J. Chudoba, A. J. Chuinard, J. J. Chwastowski, L. Chytka, A. K. Ciftci, D. Cinca, V. Cindro, I. A. Cioara, C. Ciocca, A. Ciocio, F. Cirotto, Z. H. Citron, M. Citterio, M. Ciubancan, A. Clark, B. L. Clark, M. R. Clark, P. J. Clark, R. N. Clarke, C. Clement, Y. Coadou, M. Cobal, A. Coccaro, J. Cochran, L. Colasurdo, B. Cole, A. P. Colijn, J. Collot, T. Colombo, P. Conde Muiño, E. Coniavitis, S. H. Connell, I. A. Connelly, V. Consorti, S. Constantinescu, G. Conti, F. Conventi, M. Cooke, B. D. Cooper, A. M. Cooper-Sarkar, F. Cormier, K. J. R. Cormier, T. Cornelissen, M. Corradi, F. Corriveau, A. Cortes-Gonzalez, G. Cortiana, G. Costa, M. J. Costa, D. Costanzo, G. Cottin, G. Cowan, B. E. Cox, K. Cranmer, S. J. Crawley, G. Cree, S. Crépé-Renaudin, F. Crescioli, W. A. Cribbs, M. Crispin Ortuzar, M. Cristinziani, V. Croft, G. Crosetti, A. Cueto, T. Cuhadar Donszelmann, J. Cummings, M. Curatolo, J. Cúth, H. Czirr, P. Czodrowski, G. D’amen, S. D’Auria, M. D’Onofrio, M. J. Da Cunha Sargedas De Sousa, C. Da Via, W. Dabrowski, T. Dado, T. Dai, O. Dale, F. Dallaire, C. Dallapiccola, M. Dam, J. R. Dandoy, N. P. Dang, A. C. Daniells, N. S. Dann, M. Danninger, M. Dano Hoffmann, V. Dao, G. Darbo, S. Darmora, J. Dassoulas, A. Dattagupta, W. Davey, C. David, T. Davidek, M. Davies, P. Davison, E. Dawe, I. Dawson, K. De, R. de Asmundis, A. De Benedetti, S. De Castro, S. De Cecco, N. De Groot, P. de Jong, H. De la Torre, F. De Lorenzi, A. De Maria, D. De Pedis, A. De Salvo, U. De Sanctis, A. De Santo, J. B. De Vivie De Regie, W. J. Dearnaley, R. Debbe, C. Debenedetti, D. V. Dedovich, N. Dehghanian, I. Deigaard, M. Del Gaudio, J. Del Peso, T. Del Prete, D. Delgove, F. Deliot, C. M. Delitzsch, A. Dell’Acqua, L. Dell’Asta, M. Dell’Orso, M. Della Pietra, D. della Volpe, M. Delmastro, P. A. Delsart, D. A. DeMarco, S. Demers, M. Demichev, A. Demilly, S. P. Denisov, D. Denysiuk, D. Derendarz, J. E. Derkaoui, F. Derue, P. Dervan, K. Desch, C. Deterre, K. Dette, P. O. Deviveiros, A. Dewhurst, S. Dhaliwal, A. Di Ciaccio, L. Di Ciaccio, W. K. Di Clemente, C. Di Donato, A. Di Girolamo, B. Di Girolamo, B. Di Micco, R. Di Nardo, K. F. Di Petrillo, A. Di Simone, R. Di Sipio, D. Di Valentino, C. Diaconu, M. Diamond, F. A. Dias, M. A. Diaz, E. B. Diehl, J. Dietrich, S. Díez Cornell, A. Dimitrievska, J. Dingfelder, P. Dita, S. Dita, F. Dittus, F. Djama, T. Djobava, J. I. Djuvsland, M. A. B. do Vale, D. Dobos, M. Dobre, C. Doglioni, J. Dolejsi, Z. Dolezal, M. Donadelli, S. Donati, P. Dondero, J. Donini, J. Dopke, A. Doria, M. T. Dova, A. T. Doyle, E. Drechsler, M. Dris, Y. Du, J. Duarte-Campderros, E. Duchovni, G. Duckeck, O. A. Ducu, D. Duda, A. Dudarev, A. Chr. Dudder, E. M. Duffield, L. Duflot, M. Dührssen, M. Dumancic, A. K. Duncan, M. Dunford, H. Duran Yildiz, M. Düren, A. Durglishvili, D. Duschinger, B. Dutta, M. Dyndal, C. Eckardt, K. M. Ecker, R. C. Edgar, N. C. Edwards, T. Eifert, G. Eigen, K. Einsweiler, T. Ekelof, M. El Kacimi, V. Ellajosyula, M. Ellert, S. Elles, F. Ellinghaus, A. A. Elliot, N. Ellis, J. Elmsheuser, M. Elsing, D. Emeliyanov, Y. Enari, O. C. Endner, J. S. Ennis, J. Erdmann, A. Ereditato, G. Ernis, J. Ernst, M. Ernst, S. Errede, E. Ertel, M. Escalier, H. Esch, C. Escobar, B. Esposito, A. I. Etienvre, E. Etzion, H. Evans, A. Ezhilov, M. Ezzi, F. Fabbri, L. Fabbri, G. Facini, R. M. Fakhrutdinov, S. Falciano, R. J. Falla, J. Faltova, Y. Fang, M. Fanti, A. Farbin, A. Farilla, C. Farina, E. M. Farina, T. Farooque, S. Farrell, S. M. Farrington, P. Farthouat, F. Fassi, P. Fassnacht, D. Fassouliotis, M. Faucci Giannelli, A. Favareto, W. J. Fawcett, L. Fayard, O. L. Fedin, W. Fedorko, S. Feigl, L. Feligioni, C. Feng, E. J. Feng, H. Feng, A. B. Fenyuk, L. Feremenga, P. Fernandez Martinez, S. Fernandez Perez, J. Ferrando, A. Ferrari, P. Ferrari, R. Ferrari, D. E. Ferreira de Lima, A. Ferrer, D. Ferrere, C. Ferretti, F. Fiedler, A. Filipčič, M. Filipuzzi, F. Filthaut, M. Fincke-Keeler, K. D. Finelli, M. C. N. Fiolhais, L. Fiorini, A. Fischer, C. Fischer, J. Fischer, W. C. Fisher, N. Flaschel, I. Fleck, P. Fleischmann, G. T. Fletcher, R. R. M. Fletcher, T. Flick, B. M. Flierl, L. R. Flores Castillo, M. J. Flowerdew, G. T. Forcolin, A. Formica, A. Forti, A. G. Foster, D. Fournier, H. Fox, S. Fracchia, P. Francavilla, M. Franchini, D. Francis, L. Franconi, M. Franklin, M. Frate, M. Fraternali, D. Freeborn, S. M. Fressard-Batraneanu, F. Friedrich, D. Froidevaux, J. A. Frost, C. Fukunaga, E. Fullana Torregrosa, T. Fusayasu, J. Fuster, C. Gabaldon, O. Gabizon, A. Gabrielli, A. Gabrielli, G. P. Gach, S. Gadatsch, G. Gagliardi, L. G. Gagnon, P. Gagnon, C. Galea, B. Galhardo, E. J. Gallas, B. J. Gallop, P. Gallus, G. Galster, K. K. Gan, S. Ganguly, J. Gao, Y. Gao, Y. S. Gao, F. M. Garay Walls, C. García, J. E. García Navarro, M. Garcia-Sciveres, R. W. Gardner, N. Garelli, V. Garonne, A. Gascon Bravo, K. Gasnikova, C. Gatti, A. Gaudiello, G. Gaudio, L. Gauthier, I. L. Gavrilenko, C. Gay, G. Gaycken, E. N. Gazis, Z. Gecse, C. N. P. Gee, Ch. Geich-Gimbel, M. Geisen, M. P. Geisler, K. Gellerstedt, C. Gemme, M. H. Genest, C. Geng, S. Gentile, C. Gentsos, S. George, D. Gerbaudo, A. Gershon, S. Ghasemi, M. Ghneimat, B. Giacobbe, S. Giagu, P. Giannetti, S. M. Gibson, M. Gignac, M. Gilchriese, T. P. S. Gillam, D. Gillberg, G. Gilles, D. M. Gingrich, N. Giokaris, M. P. Giordani, F. M. Giorgi, P. F. Giraud, P. Giromini, D. Giugni, F. Giuli, C. Giuliani, M. Giulini, B. K. Gjelsten, S. Gkaitatzis, I. Gkialas, E. L. Gkougkousis, L. K. Gladilin, C. Glasman, J. Glatzer, P. C. F. Glaysher, A. Glazov, M. Goblirsch-Kolb, J. Godlewski, S. Goldfarb, T. Golling, D. Golubkov, A. Gomes, R. Gonçalo, R. Goncalves Gama, J. Goncalves Pinto Firmino Da Costa, G. Gonella, L. Gonella, A. Gongadze, S. González de la Hoz, S. Gonzalez-Sevilla, L. Goossens, P. A. Gorbounov, H. A. Gordon, I. Gorelov, B. Gorini, E. Gorini, A. Gorišek, A. T. Goshaw, C. Gössling, M. I. Gostkin, C. R. Goudet, D. Goujdami, A. G. Goussiou, N. Govender, E. Gozani, L. Graber, I. Grabowska-Bold, P. O. J. Gradin, P. Grafström, J. Gramling, E. Gramstad, S. Grancagnolo, V. Gratchev, P. M. Gravila, H. M. Gray, E. Graziani, Z. D. Greenwood, C. Grefe, K. Gregersen, I. M. Gregor, P. Grenier, K. Grevtsov, J. Griffiths, A. A. Grillo, K. Grimm, S. Grinstein, Ph. Gris, J.-F. Grivaz, S. Groh, E. Gross, J. Grosse-Knetter, G. C. Grossi, Z. J. Grout, L. Guan, W. Guan, J. Guenther, F. Guescini, D. Guest, O. Gueta, B. Gui, E. Guido, T. Guillemin, S. Guindon, U. Gul, C. Gumpert, J. Guo, W. Guo, Y. Guo, R. Gupta, S. Gupta, G. Gustavino, P. Gutierrez, N. G. Gutierrez Ortiz, C. Gutschow, C. Guyot, C. Gwenlan, C. B. Gwilliam, A. Haas, C. Haber, H. K. Hadavand, N. Haddad, A. Hadef, S. Hageböck, M. Hagihara, H. Hakobyan, M. Haleem, J. Haley, G. Halladjian, G. D. Hallewell, K. Hamacher, P. Hamal, K. Hamano, A. Hamilton, G. N. Hamity, P. G. Hamnett, L. Han, S. Han, K. Hanagaki, K. Hanawa, M. Hance, B. Haney, P. Hanke, R. Hanna, J. B. Hansen, J. D. Hansen, M. C. Hansen, P. H. Hansen, K. Hara, A. S. Hard, T. Harenberg, F. Hariri, S. Harkusha, R. D. Harrington, P. F. Harrison, F. Hartjes, N. M. Hartmann, M. Hasegawa, Y. Hasegawa, A. Hasib, S. Hassani, S. Haug, R. Hauser, L. Hauswald, M. Havranek, C. M. Hawkes, R. J. Hawkings, D. Hayakawa, D. Hayden, C. P. Hays, J. M. Hays, H. S. Hayward, S. J. Haywood, S. J. Head, T. Heck, V. Hedberg, L. Heelan, S. Heim, T. Heim, B. Heinemann, J. J. Heinrich, L. Heinrich, C. Heinz, J. Hejbal, L. Helary, S. Hellman, C. Helsens, J. Henderson, R. C. W. Henderson, Y. Heng, S. Henkelmann, A. M. Henriques Correia, S. Henrot-Versille, G. H. Herbert, H. Herde, V. Herget, Y. Hernández Jiménez, G. Herten, R. Hertenberger, L. Hervas, G. G. Hesketh, N. P. Hessey, J. W. Hetherly, E. Higón-Rodriguez, E. Hill, J. C. Hill, K. H. Hiller, S. J. Hillier, I. Hinchliffe, E. Hines, M. Hirose, D. Hirschbuehl, O. Hladik, X. Hoad, J. Hobbs, N. Hod, M. C. Hodgkinson, P. Hodgson, A. Hoecker, M. R. Hoeferkamp, F. Hoenig, D. Hohn, T. R. Holmes, M. Homann, S. Honda, T. Honda, T. M. Hong, B. H. Hooberman, W. H. Hopkins, Y. Horii, A. J. Horton, J.-Y. Hostachy, S. Hou, A. Hoummada, J. Howarth, J. Hoya, M. Hrabovsky, I. Hristova, J. Hrivnac, T. Hryn’ova, A. Hrynevich, P. J. Hsu, S.-C. Hsu, Q. Hu, S. Hu, Y. Huang, Z. Hubacek, F. Hubaut, F. Huegging, T. B. Huffman, E. W. Hughes, G. Hughes, M. Huhtinen, P. Huo, N. Huseynov, J. Huston, J. Huth, G. Iacobucci, G. Iakovidis, I. Ibragimov, L. Iconomidou-Fayard, E. Ideal, Z. Idrissi, P. Iengo, O. Igonkina, T. Iizawa, Y. Ikegami, M. Ikeno, Y. Ilchenko, D. Iliadis, N. Ilic, G. Introzzi, P. Ioannou, M. Iodice, K. Iordanidou, V. Ippolito, N. Ishijima, M. Ishino, M. Ishitsuka, C. Issever, S. Istin, F. Ito, J. M. Iturbe Ponce, R. Iuppa, H. Iwasaki, J. M. Izen, V. Izzo, S. Jabbar, B. Jackson, P. Jackson, V. Jain, K. B. Jakobi, K. Jakobs, S. Jakobsen, T. Jakoubek, D. O. Jamin, D. K. Jana, R. Jansky, J. Janssen, M. Janus, P. A. Janus, G. Jarlskog, N. Javadov, T. Javůrek, M. Javurkova, F. Jeanneau, L. Jeanty, J. Jejelava, G.-Y. Jeng, P. Jenni, C. Jeske, S. Jézéquel, H. Ji, J. Jia, H. Jiang, Y. Jiang, Z. Jiang, S. Jiggins, J. Jimenez Pena, S. Jin, A. Jinaru, O. Jinnouchi, H. Jivan, P. Johansson, K. A. Johns, C. A. Johnson, W. J. Johnson, K. Jon-And, G. Jones, R. W. L. Jones, S. Jones, T. J. Jones, J. Jongmanns, P. M. Jorge, J. Jovicevic, X. Ju, A. Juste Rozas, M. K. Köhler, A. Kaczmarska, M. Kado, H. Kagan, M. Kagan, S. J. Kahn, T. Kaji, E. Kajomovitz, C. W. Kalderon, A. Kaluza, S. Kama, A. Kamenshchikov, N. Kanaya, S. Kaneti, L. Kanjir, V. A. Kantserov, J. Kanzaki, B. Kaplan, L. S. Kaplan, A. Kapliy, D. Kar, K. Karakostas, A. Karamaoun, N. Karastathis, M. J. Kareem, E. Karentzos, M. Karnevskiy, S. N. Karpov, Z. M. Karpova, K. Karthik, V. Kartvelishvili, A. N. Karyukhin, K. Kasahara, L. Kashif, R. D. Kass, A. Kastanas, Y. Kataoka, C. Kato, A. Katre, J. Katzy, K. Kawade, K. Kawagoe, T. Kawamoto, G. Kawamura, V. F. Kazanin, R. Keeler, R. Kehoe, J. S. Keller, J. J. Kempster, H. Keoshkerian, O. Kepka, B. P. Kerševan, S. Kersten, R. A. Keyes, M. Khader, F. Khalil-zada, A. Khanov, A. G. Kharlamov, T. Kharlamova, T. J. Khoo, V. Khovanskiy, E. Khramov, J. Khubua, S. Kido, C. R. Kilby, H. Y. Kim, S. H. Kim, Y. K. Kim, N. Kimura, O. M. Kind, B. T. King, M. King, D. Kirchmeier, J. Kirk, A. E. Kiryunin, T. Kishimoto, D. Kisielewska, F. Kiss, K. Kiuchi, O. Kivernyk, E. Kladiva, T. Klapdor-kleingrothaus, M. H. Klein, M. Klein, U. Klein, K. Kleinknecht, P. Klimek, A. Klimentov, R. Klingenberg, T. Klioutchnikova, E.-E. Kluge, P. Kluit, S. Kluth, J. Knapik, E. Kneringer, E. B. F. G. Knoops, A. Knue, A. Kobayashi, D. Kobayashi, T. Kobayashi, M. Kobel, M. Kocian, P. Kodys, T. Koffas, E. Koffeman, N. M. Köhler, T. Koi, H. Kolanoski, M. Kolb, I. Koletsou, A. A. Komar, Y. Komori, T. Kondo, N. Kondrashova, K. Köneke, A. C. König, T. Kono, R. Konoplich, N. Konstantinidis, R. Kopeliansky, S. Koperny, A. K. Kopp, K. Korcyl, K. Kordas, A. Korn, A. A. Korol, I. Korolkov, E. V. Korolkova, O. Kortner, S. Kortner, T. Kosek, V. V. Kostyukhin, A. Kotwal, A. Koulouris, A. Kourkoumeli-Charalampidi, C. Kourkoumelis, V. Kouskoura, A. B. Kowalewska, R. Kowalewski, T. Z. Kowalski, C. Kozakai, W. Kozanecki, A. S. Kozhin, V. A. Kramarenko, G. Kramberger, D. Krasnopevtsev, M. W. Krasny, A. Krasznahorkay, A. Kravchenko, M. Kretz, J. Kretzschmar, K. Kreutzfeldt, P. Krieger, K. Krizka, K. Kroeninger, H. Kroha, J. Kroll, J. Kroseberg, J. Krstic, U. Kruchonak, H. Krüger, N. Krumnack, M. C. Kruse, M. Kruskal, T. Kubota, H. Kucuk, S. Kuday, J. T. Kuechler, S. Kuehn, A. Kugel, F. Kuger, T. Kuhl, V. Kukhtin, R. Kukla, Y. Kulchitsky, S. Kuleshov, M. Kuna, T. Kunigo, A. Kupco, O. Kuprash, H. Kurashige, L. L. Kurchaninov, Y. A. Kurochkin, M. G. Kurth, V. Kus, E. S. Kuwertz, M. Kuze, J. Kvita, T. Kwan, D. Kyriazopoulos, A. La Rosa, J. L. La Rosa Navarro, L. La Rotonda, C. Lacasta, F. Lacava, J. Lacey, H. Lacker, D. Lacour, E. Ladygin, R. Lafaye, B. Laforge, T. Lagouri, S. Lai, S. Lammers, W. Lampl, E. Lançon, U. Landgraf, M. P. J. Landon, M. C. Lanfermann, V. S. Lang, J. C. Lange, A. J. Lankford, F. Lanni, K. Lantzsch, A. Lanza, A. Lapertosa, S. Laplace, C. Lapoire, J. F. Laporte, T. Lari, F. Lasagni Manghi, M. Lassnig, P. Laurelli, W. Lavrijsen, A. T. Law, P. Laycock, T. Lazovich, M. Lazzaroni, B. Le, O. Le Dortz, E. Le Guirriec, E. P. Le Quilleuc, M. LeBlanc, T. LeCompte, F. Ledroit-Guillon, C. A. Lee, S. C. Lee, L. Lee, B. Lefebvre, G. Lefebvre, M. Lefebvre, F. Legger, C. Leggett, A. Lehan, G. Lehmann Miotto, X. Lei, W. A. Leight, A. G. Leister, M. A. L. Leite, R. Leitner, D. Lellouch, B. Lemmer, K. J. C. Leney, T. Lenz, B. Lenzi, R. Leone, S. Leone, C. Leonidopoulos, S. Leontsinis, G. Lerner, C. Leroy, A. A. J. Lesage, C. G. Lester, M. Levchenko, J. Levêque, D. Levin, L. J. Levinson, M. Levy, A. Lewis, D. Lewis, M. Leyton, B. Li, C. Li, H. Li, L. Li, L. Li, Q. Li, S. Li, X. Li, Y. Li, Z. Liang, B. Liberti, A. Liblong, P. Lichard, K. Lie, J. Liebal, W. Liebig, A. Limosani, S. C. Lin, T. H. Lin, B. E. Lindquist, A. E. Lionti, E. Lipeles, A. Lipniacka, M. Lisovyi, T. M. Liss, A. Lister, A. M. Litke, B. Liu, D. Liu, H. Liu, H. Liu, J. Liu, J. B. Liu, K. Liu, L. Liu, M. Liu, Y. L. Liu, Y. Liu, M. Livan, A. Lleres, J. Llorente Merino, S. L. Lloyd, F. Lo Sterzo, E. M. Lobodzinska, P. Loch, F. K. Loebinger, K. M. Loew, A. Loginov, T. Lohse, K. Lohwasser, M. Lokajicek, B. A. Long, J. D. Long, R. E. Long, L. Longo, K. A. Looper, J. A. Lopez, D. Lopez Mateos, B. Lopez Paredes, I. Lopez Paz, A. Lopez Solis, J. Lorenz, N. Lorenzo Martinez, M. Losada, P. J. Lösel, X. Lou, A. Lounis, J. Love, P. A. Love, H. Lu, N. Lu, H. J. Lubatti, C. Luci, A. Lucotte, C. Luedtke, F. Luehring, W. Lukas, L. Luminari, O. Lundberg, B. Lund-Jensen, P. M. Luzi, D. Lynn, R. Lysak, E. Lytken, V. Lyubushkin, H. Ma, L. L. Ma, Y. Ma, G. Maccarrone, A. Macchiolo, C. M. Macdonald, B. Maček, J. Machado Miguens, D. Madaffari, R. Madar, H. J. Maddocks, W. F. Mader, A. Madsen, J. Maeda, S. Maeland, T. Maeno, A. Maevskiy, E. Magradze, J. Mahlstedt, C. Maiani, C. Maidantchik, A. A. Maier, T. Maier, A. Maio, S. Majewski, Y. Makida, N. Makovec, B. Malaescu, Pa. Malecki, V. P. Maleev, F. Malek, U. Mallik, D. Malon, C. Malone, S. Maltezos, S. Malyukov, J. Mamuzic, G. Mancini, L. Mandelli, I. Mandić, J. Maneira, L. Manhaes de Andrade Filho, J. Manjarres Ramos, A. Mann, A. Manousos, B. Mansoulie, J. D. Mansour, R. Mantifel, M. Mantoani, S. Manzoni, L. Mapelli, G. Marceca, L. March, G. Marchiori, M. Marcisovsky, M. Marjanovic, D. E. Marley, F. Marroquim, S. P. Marsden, Z. Marshall, S. Marti-Garcia, B. Martin, T. A. Martin, V. J. Martin, B. Martin dit Latour, M. Martinez, V. I. Martinez Outschoorn, S. Martin-Haugh, V. S. Martoiu, A. C. Martyniuk, A. Marzin, L. Masetti, T. Mashimo, R. Mashinistov, J. Masik, A. L. Maslennikov, I. Massa, L. Massa, P. Mastrandrea, A. Mastroberardino, T. Masubuchi, P. Mättig, J. Mattmann, J. Maurer, S. J. Maxfield, D. A. Maximov, R. Mazini, I. Maznas, S. M. Mazza, N. C. Mc Fadden, G. Mc Goldrick, S. P. Mc Kee, A. McCarn, R. L. McCarthy, T. G. McCarthy, L. I. McClymont, E. F. McDonald, J. A. Mcfayden, G. Mchedlidze, S. J. McMahon, R. A. McPherson, M. Medinnis, S. Meehan, S. Mehlhase, A. Mehta, K. Meier, C. Meineck, B. Meirose, D. Melini, B. R. Mellado Garcia, M. Melo, F. Meloni, S. B. Menary, L. Meng, X. T. Meng, A. Mengarelli, S. Menke, E. Meoni, S. Mergelmeyer, P. Mermod, L. Merola, C. Meroni, F. S. Merritt, A. Messina, J. Metcalfe, A. S. Mete, C. Meyer, C. Meyer, J.-P. Meyer, J. Meyer, H. Meyer Zu Theenhausen, F. Miano, R. P. Middleton, S. Miglioranzi, L. Mijović, G. Mikenberg, M. Mikestikova, M. Mikuž, M. Milesi, A. Milic, D. W. Miller, C. Mills, A. Milov, D. A. Milstead, A. A. Minaenko, Y. Minami, I. A. Minashvili, A. I. Mincer, B. Mindur, M. Mineev, Y. Minegishi, Y. Ming, L. M. Mir, K. P. Mistry, T. Mitani, J. Mitrevski, V. A. Mitsou, A. Miucci, P. S. Miyagawa, A. Mizukami, J. U. Mjörnmark, M. Mlynarikova, T. Moa, K. Mochizuki, P. Mogg, S. Mohapatra, S. Molander, R. Moles-Valls, R. Monden, M. C. Mondragon, K. Mönig, J. Monk, E. Monnier, A. Montalbano, J. Montejo Berlingen, F. Monticelli, S. Monzani, R. W. Moore, N. Morange, D. Moreno, M. Moreno Llácer, P. Morettini, S. Morgenstern, D. Mori, T. Mori, M. Morii, M. Morinaga, V. Morisbak, S. Moritz, A. K. Morley, G. Mornacchi, J. D. Morris, L. Morvaj, P. Moschovakos, M. Mosidze, H. J. Moss, J. Moss, K. Motohashi, R. Mount, E. Mountricha, E. J. W. Moyse, S. Muanza, R. D. Mudd, F. Mueller, J. Mueller, R. S. P. Mueller, T. Mueller, D. Muenstermann, P. Mullen, G. A. Mullier, F. J. Munoz Sanchez, J. A. Murillo Quijada, W. J. Murray, H. Musheghyan, M. Muškinja, A. G. Myagkov, M. Myska, B. P. Nachman, O. Nackenhorst, K. Nagai, R. Nagai, K. Nagano, Y. Nagasaka, K. Nagata, M. Nagel, E. Nagy, A. M. Nairz, Y. Nakahama, K. Nakamura, T. Nakamura, I. Nakano, R. F. Naranjo Garcia, R. Narayan, D. I. Narrias Villar, I. Naryshkin, T. Naumann, G. Navarro, R. Nayyar, H. A. Neal, P. Yu. Nechaeva, T. J. Neep, A. Negri, M. Negrini, S. Nektarijevic, C. Nellist, A. Nelson, S. Nemecek, P. Nemethy, A. A. Nepomuceno, M. Nessi, M. S. Neubauer, M. Neumann, R. M. Neves, P. Nevski, P. R. Newman, T. Nguyen Manh, R. B. Nickerson, R. Nicolaidou, J. Nielsen, V. Nikolaenko, I. Nikolic-Audit, K. Nikolopoulos, J. K. Nilsen, P. Nilsson, Y. Ninomiya, A. Nisati, R. Nisius, T. Nobe, M. Nomachi, I. Nomidis, T. Nooney, S. Norberg, M. Nordberg, N. Norjoharuddeen, O. Novgorodova, S. Nowak, M. Nozaki, L. Nozka, K. Ntekas, E. Nurse, F. Nuti, D. C. O’Neil, A. A. O’Rourke, V. O’Shea, F. G. Oakham, H. Oberlack, T. Obermann, J. Ocariz, A. Ochi, I. Ochoa, J. P. Ochoa-Ricoux, S. Oda, S. Odaka, H. Ogren, A. Oh, S. H. Oh, C. C. Ohm, H. Ohman, H. Oide, H. Okawa, Y. Okumura, T. Okuyama, A. Olariu, L. F. Oleiro Seabra, S. A. Olivares Pino, D. Oliveira Damazio, A. Olszewski, J. Olszowska, A. Onofre, K. Onogi, P. U. E. Onyisi, M. J. Oreglia, Y. Oren, D. Orestano, N. Orlando, R. S. Orr, B. Osculati, R. Ospanov, G. Otero y Garzon, H. Otono, M. Ouchrif, F. Ould-Saada, A. Ouraou, K. P. Oussoren, Q. Ouyang, M. Owen, R. E. Owen, V. E. Ozcan, N. Ozturk, K. Pachal, A. Pacheco Pages, L. Pacheco Rodriguez, C. Padilla Aranda, S. Pagan Griso, M. Paganini, F. Paige, P. Pais, K. Pajchel, G. Palacino, S. Palazzo, S. Palestini, M. Palka, D. Pallin, E. St. Panagiotopoulou, I. Panagoulias, C. E. Pandini, J. G. Panduro Vazquez, P. Pani, S. Panitkin, D. Pantea, L. Paolozzi, Th. D. Papadopoulou, K. Papageorgiou, A. Paramonov, D. Paredes Hernandez, A. J. Parker, M. A. Parker, K. A. Parker, F. Parodi, J. A. Parsons, U. Parzefall, V. R. Pascuzzi, E. Pasqualucci, S. Passaggio, Fr. Pastore, G. Pásztor, S. Pataraia, J. R. Pater, T. Pauly, J. Pearce, B. Pearson, L. E. Pedersen, M. Pedersen, S. Pedraza Lopez, R. Pedro, S. V. Peleganchuk, O. Penc, C. Peng, H. Peng, J. Penwell, B. S. Peralva, M. M. Perego, D. V. Perepelitsa, E. Perez Codina, L. Perini, H. Pernegger, S. Perrella, R. Peschke, V. D. Peshekhonov, K. Peters, R. F. Y. Peters, B. A. Petersen, T. C. Petersen, E. Petit, A. Petridis, C. Petridou, P. Petroff, E. Petrolo, M. Petrov, F. Petrucci, N. E. Pettersson, A. Peyaud, R. Pezoa, P. W. Phillips, G. Piacquadio, E. Pianori, A. Picazio, E. Piccaro, M. Piccinini, M. A. Pickering, R. Piegaia, J. E. Pilcher, A. D. Pilkington, A. W. J. Pin, M. Pinamonti, J. L. Pinfold, A. Pingel, S. Pires, H. Pirumov, M. Pitt, L. Plazak, M.-A. Pleier, V. Pleskot, E. Plotnikova, D. Pluth, R. Poettgen, L. Poggioli, D. Pohl, G. Polesello, A. Poley, A. Policicchio, R. Polifka, A. Polini, C. S. Pollard, V. Polychronakos, K. Pommès, L. Pontecorvo, B. G. Pope, G. A. Popeneciu, A. Poppleton, S. Pospisil, K. Potamianos, I. N. Potrap, C. J. Potter, C. T. Potter, G. Poulard, J. Poveda, V. Pozdnyakov, M. E. Pozo Astigarraga, P. Pralavorio, A. Pranko, S. Prell, D. Price, L. E. Price, M. Primavera, S. Prince, K. Prokofiev, F. Prokoshin, S. Protopopescu, J. Proudfoot, M. Przybycien, D. Puddu, M. Purohit, P. Puzo, J. Qian, G. Qin, Y. Qin, A. Quadt, W. B. Quayle, M. Queitsch-Maitland, D. Quilty, S. Raddum, V. Radeka, V. Radescu, S. K. Radhakrishnan, P. Radloff, P. Rados, F. Ragusa, G. Rahal, J. A. Raine, S. Rajagopalan, M. Rammensee, C. Rangel-Smith, M. G. Ratti, D. M. Rauch, F. Rauscher, S. Rave, T. Ravenscroft, I. Ravinovich, M. Raymond, A. L. Read, N. P. Readioff, M. Reale, D. M. Rebuzzi, A. Redelbach, G. Redlinger, R. Reece, R. G. Reed, K. Reeves, L. Rehnisch, J. Reichert, A. Reiss, C. Rembser, H. Ren, M. Rescigno, S. Resconi, E. D. Resseguie, O. L. Rezanova, P. Reznicek, R. Rezvani, R. Richter, S. Richter, E. Richter-Was, O. Ricken, M. Ridel, P. Rieck, C. J. Riegel, J. Rieger, O. Rifki, M. Rijssenbeek, A. Rimoldi, M. Rimoldi, L. Rinaldi, B. Ristić, E. Ritsch, I. Riu, F. Rizatdinova, E. Rizvi, C. Rizzi, R. T. Roberts, S. H. Robertson, A. Robichaud-Veronneau, D. Robinson, J. E. M. Robinson, A. Robson, C. Roda, Y. Rodina, A. Rodriguez Perez, D. Rodriguez Rodriguez, S. Roe, C. S. Rogan, O. Røhne, J. Roloff, A. Romaniouk, M. Romano, S. M. Romano Saez, E. Romero Adam, N. Rompotis, M. Ronzani, L. Roos, E. Ros, S. Rosati, K. Rosbach, P. Rose, N.-A. Rosien, V. Rossetti, E. Rossi, L. P. Rossi, J. H. N. Rosten, R. Rosten, M. Rotaru, I. Roth, J. Rothberg, D. Rousseau, A. Rozanov, Y. Rozen, X. Ruan, F. Rubbo, M. S. Rudolph, F. Rühr, A. Ruiz-Martinez, Z. Rurikova, N. A. Rusakovich, A. Ruschke, H. L. Russell, J. P. Rutherfoord, N. Ruthmann, Y. F. Ryabov, M. Rybar, G. Rybkin, S. Ryu, A. Ryzhov, G. F. Rzehorz, A. F. Saavedra, G. Sabato, S. Sacerdoti, H. F.-W. Sadrozinski, R. Sadykov, F. Safai Tehrani, P. Saha, M. Sahinsoy, M. Saimpert, T. Saito, H. Sakamoto, Y. Sakurai, G. Salamanna, A. Salamon, J. E. Salazar Loyola, D. Salek, P. H. Sales De Bruin, D. Salihagic, A. Salnikov, J. Salt, D. Salvatore, F. Salvatore, A. Salvucci, A. Salzburger, D. Sammel, D. Sampsonidis, J. Sánchez, V. Sanchez Martinez, A. Sanchez Pineda, H. Sandaker, R. L. Sandbach, M. Sandhoff, C. Sandoval, D. P. C. Sankey, M. Sannino, A. Sansoni, C. Santoni, R. Santonico, H. Santos, I. Santoyo Castillo, K. Sapp, A. Sapronov, J. G. Saraiva, B. Sarrazin, O. Sasaki, K. Sato, E. Sauvan, G. Savage, P. Savard, N. Savic, C. Sawyer, L. Sawyer, J. Saxon, C. Sbarra, A. Sbrizzi, T. Scanlon, D. A. Scannicchio, M. Scarcella, V. Scarfone, J. Schaarschmidt, P. Schacht, B. M. Schachtner, D. Schaefer, L. Schaefer, R. Schaefer, J. Schaeffer, S. Schaepe, S. Schaetzel, U. Schäfer, A. C. Schaffer, D. Schaile, R. D. Schamberger, V. Scharf, V. A. Schegelsky, D. Scheirich, M. Schernau, C. Schiavi, S. Schier, C. Schillo, M. Schioppa, S. Schlenker, K. R. Schmidt-Sommerfeld, K. Schmieden, C. Schmitt, S. Schmitt, S. Schmitt, S. Schmitz, B. Schneider, U. Schnoor, L. Schoeffel, A. Schoening, B. D. Schoenrock, E. Schopf, M. Schott, J. F. P. Schouwenberg, J. Schovancova, S. Schramm, M. Schreyer, N. Schuh, A. Schulte, M. J. Schultens, H.-C. Schultz-Coulon, H. Schulz, M. Schumacher, B. A. Schumm, Ph. Schune, A. Schwartzman, T. A. Schwarz, H. Schweiger, Ph. Schwemling, R. Schwienhorst, J. Schwindling, T. Schwindt, G. Sciolla, F. Scuri, F. Scutti, J. Searcy, P. Seema, S. C. Seidel, A. Seiden, F. Seifert, J. M. Seixas, G. Sekhniaidze, K. Sekhon, S. J. Sekula, N. Semprini-Cesari, C. Serfon, L. Serin, L. Serkin, M. Sessa, R. Seuster, H. Severini, T. Sfiligoj, F. Sforza, A. Sfyrla, E. Shabalina, N. W. Shaikh, L. Y. Shan, R. Shang, J. T. Shank, M. Shapiro, P. B. Shatalov, K. Shaw, S. M. Shaw, A. Shcherbakova, C. Y. Shehu, P. Sherwood, L. Shi, S. Shimizu, C. O. Shimmin, M. Shimojima, S. Shirabe, M. Shiyakova, A. Shmeleva, D. Shoaleh Saadi, M. J. Shochet, S. Shojaii, D. R. Shope, S. Shrestha, E. Shulga, M. A. Shupe, P. Sicho, A. M. Sickles, P. E. Sidebo, E. Sideras Haddad, O. Sidiropoulou, D. Sidorov, A. Sidoti, F. Siegert, Dj. Sijacki, J. Silva, S. B. Silverstein, V. Simak, Lj. Simic, S. Simion, E. Simioni, B. Simmons, D. Simon, M. Simon, P. Sinervo, N. B. Sinev, M. Sioli, G. Siragusa, I. Siral, S. Yu. Sivoklokov, J. Sjölin, M. B. Skinner, H. P. Skottowe, P. Skubic, M. Slater, T. Slavicek, M. Slawinska, K. Sliwa, R. Slovak, V. Smakhtin, B. H. Smart, L. Smestad, J. Smiesko, S. Yu. Smirnov, Y. Smirnov, L. N. Smirnova, O. Smirnova, J. W. Smith, M. N. K. Smith, R. W. Smith, M. Smizanska, K. Smolek, A. A. Snesarev, I. M. Snyder, S. Snyder, R. Sobie, F. Socher, A. Soffer, D. A. Soh, G. Sokhrannyi, C. A. Solans Sanchez, M. Solar, E. Yu. Soldatov, U. Soldevila, A. A. Solodkov, A. Soloshenko, O. V. Solovyanov, V. Solovyev, P. Sommer, H. Son, H. Y. Song, A. Sood, A. Sopczak, V. Sopko, V. Sorin, D. Sosa, C. L. Sotiropoulou, R. Soualah, A. M. Soukharev, D. South, B. C. Sowden, S. Spagnolo, M. Spalla, M. Spangenberg, F. Spanò, D. Sperlich, F. Spettel, R. Spighi, G. Spigo, L. A. Spiller, M. Spousta, R. D. St. Denis, A. Stabile, R. Stamen, S. Stamm, E. Stanecka, R. W. Stanek, C. Stanescu, M. Stanescu-Bellu, M. M. Stanitzki, S. Stapnes, E. A. Starchenko, G. H. Stark, J. Stark, S. H Stark, P. Staroba, P. Starovoitov, S. Stärz, R. Staszewski, P. Steinberg, B. Stelzer, H. J. Stelzer, O. Stelzer-Chilton, H. Stenzel, G. A. Stewart, J. A. Stillings, M. C. Stockton, M. Stoebe, G. Stoicea, P. Stolte, S. Stonjek, A. R. Stradling, A. Straessner, M. E. Stramaglia, J. Strandberg, S. Strandberg, A. Strandlie, M. Strauss, P. Strizenec, R. Ströhmer, D. M. Strom, R. Stroynowski, A. Strubig, S. A. Stucci, B. Stugu, N. A. Styles, D. Su, J. Su, S. Suchek, Y. Sugaya, M. Suk, V. V. Sulin, S. Sultansoy, T. Sumida, S. Sun, X. Sun, J. E. Sundermann, K. Suruliz, C. J. E. Suster, M. R. Sutton, S. Suzuki, M. Svatos, M. Swiatlowski, S. P. Swift, I. Sykora, T. Sykora, D. Ta, K. Tackmann, J. Taenzer, A. Taffard, R. Tafirout, N. Taiblum, H. Takai, R. Takashima, T. Takeshita, Y. Takubo, M. Talby, A. A. Talyshev, J. Tanaka, M. Tanaka, R. Tanaka, S. Tanaka, R. Tanioka, B. B. Tannenwald, S. Tapia Araya, S. Tapprogge, S. Tarem, G. F. Tartarelli, P. Tas, M. Tasevsky, T. Tashiro, E. Tassi, A. Tavares Delgado, Y. Tayalati, A. C. Taylor, G. N. Taylor, P. T. E. Taylor, W. Taylor, F. A. Teischinger, P. Teixeira-Dias, K. K. Temming, D. Temple, H. Ten Kate, P. K. Teng, J. J. Teoh, F. Tepel, S. Terada, K. Terashi, J. Terron, S. Terzo, M. Testa, R. J. Teuscher, T. Theveneaux-Pelzer, J. P. Thomas, J. Thomas-Wilsker, P. D. Thompson, A. S. Thompson, L. A. Thomsen, E. Thomson, M. J. Tibbetts, R. E. Ticse Torres, V. O. Tikhomirov, Yu. A. Tikhonov, S. Timoshenko, P. Tipton, S. Tisserant, K. Todome, T. Todorov, S. Todorova-Nova, J. Tojo, S. Tokár, K. Tokushuku, E. Tolley, L. Tomlinson, M. Tomoto, L. Tompkins, K. Toms, B. Tong, P. Tornambe, E. Torrence, H. Torres, E. Torró Pastor, J. Toth, F. Touchard, D. R. Tovey, T. Trefzger, A. Tricoli, I. M. Trigger, S. Trincaz-Duvoid, M. F. Tripiana, W. Trischuk, B. Trocmé, A. Trofymov, C. Troncon, M. Trottier-McDonald, M. Trovatelli, L. Truong, M. Trzebinski, A. Trzupek, J. C.-L. Tseng, P. V. Tsiareshka, G. Tsipolitis, N. Tsirintanis, S. Tsiskaridze, V. Tsiskaridze, E. G. Tskhadadze, K. M. Tsui, I. I. Tsukerman, V. Tsulaia, S. Tsuno, D. Tsybychev, Y. Tu, A. Tudorache, V. Tudorache, T. T. Tulbure, A. N. Tuna, S. A. Tupputi, S. Turchikhin, D. Turgeman, I. Turk Cakir, R. Turra, P. M. Tuts, G. Ucchielli, I. Ueda, M. Ughetto, F. Ukegawa, G. Unal, A. Undrus, G. Unel, F. C. Ungaro, Y. Unno, C. Unverdorben, J. Urban, P. Urquijo, P. Urrejola, G. Usai, J. Usui, L. Vacavant, V. Vacek, B. Vachon, C. Valderanis, E. Valdes Santurio, N. Valencic, S. Valentinetti, A. Valero, L. Valery, S. Valkar, J. A. Valls Ferrer, W. Van Den Wollenberg, P. C. Van Der Deijl, H. van der Graaf, N. van Eldik, P. van Gemmeren, J. Van Nieuwkoop, I. van Vulpen, M. C. van Woerden, M. Vanadia, W. Vandelli, R. Vanguri, A. Vaniachine, P. Vankov, G. Vardanyan, R. Vari, E. W. Varnes, T. Varol, D. Varouchas, A. Vartapetian, K. E. Varvell, J. G. Vasquez, G. A. Vasquez, F. Vazeille, T. Vazquez Schroeder, J. Veatch, V. Veeraraghavan, L. M. Veloce, F. Veloso, S. Veneziano, A. Ventura, M. Venturi, N. Venturi, A. Venturini, V. Vercesi, M. Verducci, W. Verkerke, J. C. Vermeulen, A. Vest, M. C. Vetterli, O. Viazlo, I. Vichou, T. Vickey, O. E. Vickey Boeriu, G. H. A. Viehhauser, S. Viel, L. Vigani, M. Villa, M. Villaplana Perez, E. Vilucchi, M. G. Vincter, V. B. Vinogradov, A. Vishwakarma, C. Vittori, I. Vivarelli, S. Vlachos, M. Vlasak, M. Vogel, P. Vokac, G. Volpi, M. Volpi, H. von der Schmitt, E. von Toerne, V. Vorobel, K. Vorobev, M. Vos, R. Voss, J. H. Vossebeld, N. Vranjes, M. Vranjes Milosavljevic, V. Vrba, M. Vreeswijk, R. Vuillermet, I. Vukotic, P. Wagner, W. Wagner, H. Wahlberg, S. Wahrmund, J. Wakabayashi, J. Walder, R. Walker, W. Walkowiak, V. Wallangen, C. Wang, C. Wang, F. Wang, H. Wang, H. Wang, J. Wang, J. Wang, K. Wang, Q. Wang, R. Wang, S. M. Wang, T. Wang, W. Wang, C. Wanotayaroj, A. Warburton, C. P. Ward, D. R. Wardrope, A. Washbrook, P. M. Watkins, A. T. Watson, M. F. Watson, G. Watts, S. Watts, B. M. Waugh, S. Webb, M. S. Weber, S. W. Weber, S. A. Weber, J. S. Webster, A. R. Weidberg, B. Weinert, J. Weingarten, C. Weiser, H. Weits, P. S. Wells, T. Wenaus, T. Wengler, S. Wenig, N. Wermes, M. D. Werner, P. Werner, M. Wessels, J. Wetter, K. Whalen, N. L. Whallon, A. M. Wharton, A. White, M. J. White, R. White, D. Whiteson, F. J. Wickens, W. Wiedenmann, M. Wielers, C. Wiglesworth, L. A. M. Wiik-Fuchs, A. Wildauer, F. Wilk, H. G. Wilkens, H. H. Williams, S. Williams, C. Willis, S. Willocq, J. A. Wilson, I. Wingerter-Seez, F. Winklmeier, O. J. Winston, B. T. Winter, M. Wittgen, M. Wobisch, T. M. H. Wolf, R. Wolff, M. W. Wolter, H. Wolters, S. D. Worm, B. K. Wosiek, J. Wotschack, M. J. Woudstra, K. W. Wozniak, M. Wu, M. Wu, S. L. Wu, X. Wu, Y. Wu, T. R. Wyatt, B. M. Wynne, S. Xella, Z. Xi, D. Xu, L. Xu, B. Yabsley, S. Yacoob, D. Yamaguchi, Y. Yamaguchi, A. Yamamoto, S. Yamamoto, T. Yamanaka, K. Yamauchi, Y. Yamazaki, Z. Yan, H. Yang, H. Yang, Y. Yang, Z. Yang, W.-M. Yao, Y. C. Yap, Y. Yasu, E. Yatsenko, K. H. Yau Wong, J. Ye, S. Ye, I. Yeletskikh, E. Yildirim, K. Yorita, R. Yoshida, K. Yoshihara, C. Young, C. J. S. Young, S. Youssef, D. R. Yu, J. Yu, J. M. Yu, J. Yu, L. Yuan, S. P. Y. Yuen, I. Yusuff, B. Zabinski, G. Zacharis, R. Zaidan, A. M. Zaitsev, N. Zakharchuk, J. Zalieckas, A. Zaman, S. Zambito, D. Zanzi, C. Zeitnitz, M. Zeman, A. Zemla, J. C. Zeng, Q. Zeng, O. Zenin, T. Ženiš, D. Zerwas, D. Zhang, F. Zhang, G. Zhang, H. Zhang, J. Zhang, L. Zhang, L. Zhang, M. Zhang, R. Zhang, R. Zhang, X. Zhang, Y. Zhang, Z. Zhang, X. Zhao, Y. Zhao, Z. Zhao, A. Zhemchugov, J. Zhong, B. Zhou, C. Zhou, L. Zhou, L. Zhou, M. Zhou, M. Zhou, N. Zhou, C. G. Zhu, H. Zhu, J. Zhu, Y. Zhu, X. Zhuang, K. Zhukov, A. Zibell, D. Zieminska, N. I. Zimine, C. Zimmermann, S. Zimmermann, Z. Zinonos, M. Zinser, M. Ziolkowski, L. Živković, G. Zobernig, A. Zoccoli, M. zur Nedden, L. Zwalinski, ATLAS Collaboration

**Affiliations:** 1 Department of Physics, University of Adelaide, Adelaide, Australia; 2 Physics Department, SUNY Albany, Albany, NY USA; 3 Department of Physics, University of Alberta, Edmonton, AB Canada; 4 Department of Physics, Ankara University, Ankara, Turkey; 5 Istanbul Aydin University, Istanbul, Turkey; 6 Division of Physics, TOBB University of Economics and Technology, Ankara, Turkey; 7 LAPP, CNRS/IN2P3 and Université Savoie Mont Blanc, Annecy-le-Vieux, France; 8 High Energy Physics Division, Argonne National Laboratory, Argonne, IL USA; 9 Department of Physics, University of Arizona, Tucson, AZ USA; 10 Department of Physics, The University of Texas at Arlington, Arlington, TX USA; 11 Physics Department, National and Kapodistrian University of Athens, Athens, Greece; 12 Physics Department, National Technical University of Athens, Zografou, Greece; 13 Department of Physics, The University of Texas at Austin, Austin, TX USA; 14 Institute of Physics, Azerbaijan Academy of Sciences, Baku, Azerbaijan; 15 Institut de Física d’Altes Energies (IFAE), The Barcelona Institute of Science and Technology, Barcelona, Spain; 16 Institute of Physics, University of Belgrade, Belgrade, Serbia; 17 Department for Physics and Technology, University of Bergen, Bergen, Norway; 18 Physics Division, Lawrence Berkeley National Laboratory and University of California, Berkeley, CA USA; 19 Department of Physics, Humboldt University, Berlin, Germany; 20 Albert Einstein Center for Fundamental Physics and Laboratory for High Energy Physics, University of Bern, Bern, Switzerland; 21 School of Physics and Astronomy, University of Birmingham, Birmingham, UK; 22 Department of Physics, Bogazici University, Istanbul, Turkey; 23 Department of Physics Engineering, Gaziantep University, Gaziantep, Turkey; 24 Faculty of Engineering and Natural Sciences, Istanbul Bilgi University, Istanbul, Turkey; 25 Faculty of Engineering and Natural Sciences, Bahcesehir University, Istanbul, Turkey; 26 Centro de Investigaciones, Universidad Antonio Narino, Bogotá, Colombia; 27 INFN Sezione di Bologna, Bologna, Italy; 28 Dipartimento di Fisica e Astronomia, Università di Bologna, Bologna, Italy; 29 Physikalisches Institut, University of Bonn, Bonn, Germany; 30 Department of Physics, Boston University, Boston, MA USA; 31 Department of Physics, Brandeis University, Waltham, MA USA; 32 Universidade Federal do Rio De Janeiro COPPE/EE/IF, Rio de Janeiro, Brazil; 33 Electrical Circuits Department, Federal University of Juiz de Fora (UFJF), Juiz de Fora, Brazil; 34 Federal University of Sao Joao del Rei (UFSJ), Sao Joao del Rei, Brazil; 35 Instituto de Fisica, Universidade de Sao Paulo, Sao Paulo, Brazil; 36 Physics Department, Brookhaven National Laboratory, Upton, NY USA; 37 Transilvania University of Brasov, Brasov, Romania; 38 Horia Hulubei National Institute of Physics and Nuclear Engineering, Bucharest, Romania; 39 Physics Department, National Institute for Research and Development of Isotopic and Molecular Technologies, Cluj-Napoca, Romania; 40 University Politehnica Bucharest, Bucharest, Romania; 41 West University in Timisoara, Timisoara, Romania; 42 Departamento de Física, Universidad de Buenos Aires, Buenos Aires, Argentina; 43 Cavendish Laboratory, University of Cambridge, Cambridge, UK; 44 Department of Physics, Carleton University, Ottawa, ON Canada; 45 CERN, Geneva, Switzerland; 46 Enrico Fermi Institute, University of Chicago, Chicago, IL USA; 47 Departamento de Física, Pontificia Universidad Católica de Chile, Santiago, Chile; 48 Departamento de Física, Universidad Técnica Federico Santa María, Valparaiso, Chile; 49 Institute of High Energy Physics, Chinese Academy of Sciences, Beijing, China; 50 Department of Physics, Nanjing University, Nanjing, Jiangsu China; 51 Physics Department, Tsinghua University, Beijing, 100084 China; 52 Department of Modern Physics, University of Science and Technology of China, Anhui, China; 53 School of Physics, Shandong University, Jinan, Shandong China; 54 Department of Physics and Astronomy, Key Laboratory for Particle Physics, Astrophysics and Cosmology, Ministry of Education; Shanghai Key Laboratory for Particle Physics and Cosmology (SKLPPC), Shanghai Jiao Tong University, Shanghai, China; 55 Laboratoire de Physique Corpusculaire, Université Clermont Auvergne, Université Blaise Pascal, CNRS/IN2P3, Clermont-Ferrand, France; 56 Nevis Laboratory, Columbia University, Irvington, NY USA; 57 Niels Bohr Institute, University of Copenhagen, Copenhagen, Denmark; 58 INFN Gruppo Collegato di Cosenza, Laboratori Nazionali di Frascati, Frascati, Italy; 59 Dipartimento di Fisica, Università della Calabria, Rende, Italy; 60 Faculty of Physics and Applied Computer Science, AGH University of Science and Technology, Kraków, Poland; 61 Marian Smoluchowski Institute of Physics, Jagiellonian University, Kraków, Poland; 62 Institute of Nuclear Physics Polish Academy of Sciences, Kraków, Poland; 63 Physics Department, Southern Methodist University, Dallas, TX USA; 64 Physics Department, University of Texas at Dallas, Richardson, TX USA; 65 DESY, Hamburg and Zeuthen, Germany; 66 Lehrstuhl für Experimentelle Physik IV, Technische Universität Dortmund, Dortmund, Germany; 67 Institut für Kern- und Teilchenphysik, Technische Universität Dresden, Dresden, Germany; 68 Department of Physics, Duke University, Durham, NC USA; 69 SUPA-School of Physics and Astronomy, University of Edinburgh, Edinburgh, UK; 70 INFN Laboratori Nazionali di Frascati, Frascati, Italy; 71 Fakultät für Mathematik und Physik, Albert-Ludwigs-Universität, Freiburg, Germany; 72 Departement de Physique Nucleaire et Corpusculaire, Université de Genève, Geneva, Switzerland; 73 INFN Sezione di Genova, Genoa, Italy; 74 Dipartimento di Fisica, Università di Genova, Genoa, Italy; 75 E. Andronikashvili Institute of Physics, Iv. Javakhishvili Tbilisi State University, Tbilisi, Georgia; 76 High Energy Physics Institute, Tbilisi State University, Tbilisi, Georgia; 77 II Physikalisches Institut, Justus-Liebig-Universität Giessen, Giessen, Germany; 78 SUPA-School of Physics and Astronomy, University of Glasgow, Glasgow, UK; 79 II Physikalisches Institut, Georg-August-Universität, Göttingen, Germany; 80 Laboratoire de Physique Subatomique et de Cosmologie, Université Grenoble-Alpes, CNRS/IN2P3, Grenoble, France; 81 Laboratory for Particle Physics and Cosmology, Harvard University, Cambridge, MA USA; 82 Kirchhoff-Institut für Physik, Ruprecht-Karls-Universität Heidelberg, Heidelberg, Germany; 83 Physikalisches Institut, Ruprecht-Karls-Universität Heidelberg, Heidelberg, Germany; 84 ZITI Institut für technische Informatik, Ruprecht-Karls-Universität Heidelberg, Mannheim, Germany; 85 Faculty of Applied Information Science, Hiroshima Institute of Technology, Hiroshima, Japan; 86 Department of Physics, The Chinese University of Hong Kong, Shatin, N.T. Hong Kong; 87 Department of Physics, The University of Hong Kong, Hong Kong, China; 88 Department of Physics and Institute for Advanced Study, The Hong Kong University of Science and Technology, Clear Water Bay, Kowloon, Hong Kong, China; 89 Department of Physics, National Tsing Hua University, Hsinchu, Taiwan; 90 Department of Physics, Indiana University, Bloomington, IN USA; 91 Institut für Astro- und Teilchenphysik, Leopold-Franzens-Universität, Innsbruck, Austria; 92 University of Iowa, Iowa City, IA USA; 93 Department of Physics and Astronomy, Iowa State University, Ames, IA USA; 94 Joint Institute for Nuclear Research, JINR Dubna, Dubna, Russia; 95 KEK, High Energy Accelerator Research Organization, Tsukuba, Japan; 96 Graduate School of Science, Kobe University, Kobe, Japan; 97 Faculty of Science, Kyoto University, Kyoto, Japan; 98 Kyoto University of Education, Kyoto, Japan; 99 Department of Physics, Kyushu University, Fukuoka, Japan; 100 Instituto de Física La Plata, Universidad Nacional de La Plata and CONICET, La Plata, Argentina; 101 Physics Department, Lancaster University, Lancaster, UK; 102 INFN Sezione di Lecce, Lecce, Italy; 103 Dipartimento di Matematica e Fisica, Università del Salento, Lecce, Italy; 104 Oliver Lodge Laboratory, University of Liverpool, Liverpool, UK; 105 Department of Experimental Particle Physics, Jožef Stefan Institute and Department of Physics, University of Ljubljana, Ljubljana, Slovenia; 106 School of Physics and Astronomy, Queen Mary University of London, London, UK; 107 Department of Physics, Royal Holloway University of London, Surrey, UK; 108 Department of Physics and Astronomy, University College London, London, UK; 109 Louisiana Tech University, Ruston, LA USA; 110 Laboratoire de Physique Nucléaire et de Hautes Energies, UPMC and Université Paris-Diderot and CNRS/IN2P3, Paris, France; 111 Fysiska institutionen, Lunds universitet, Lund, Sweden; 112 Departamento de Fisica Teorica C-15, Universidad Autonoma de Madrid, Madrid, Spain; 113 Institut für Physik, Universität Mainz, Mainz, Germany; 114 School of Physics and Astronomy, University of Manchester, Manchester, UK; 115 CPPM, Aix-Marseille Université and CNRS/IN2P3, Marseille, France; 116 Department of Physics, University of Massachusetts, Amherst, MA USA; 117 Department of Physics, McGill University, Montreal, QC Canada; 118 School of Physics, University of Melbourne, Melbourne, VIC Australia; 119 Department of Physics, The University of Michigan, Ann Arbor, MI USA; 120 Department of Physics and Astronomy, Michigan State University, East Lansing, MI USA; 121 INFN Sezione di Milano, Milan, Italy; 122 Dipartimento di Fisica, Università di Milano, Milan, Italy; 123 B.I. Stepanov Institute of Physics, National Academy of Sciences of Belarus, Minsk, Republic of Belarus; 124 Research Institute for Nuclear Problems of Byelorussian State University, Minsk, Republic of Belarus; 125 Group of Particle Physics, University of Montreal, Montreal, QC Canada; 126 P.N. Lebedev Physical Institute of the Russian Academy of Sciences, Moscow, Russia; 127 Institute for Theoretical and Experimental Physics (ITEP), Moscow, Russia; 128 National Research Nuclear University MEPhI, Moscow, Russia; 129 D.V. Skobeltsyn Institute of Nuclear Physics, M.V. Lomonosov Moscow State University, Moscow, Russia; 130 Fakultät für Physik, Ludwig-Maximilians-Universität München, Munich, Germany; 131 Max-Planck-Institut für Physik (Werner-Heisenberg-Institut), Munich, Germany; 132 Nagasaki Institute of Applied Science, Nagasaki, Japan; 133 Graduate School of Science and Kobayashi-Maskawa Institute, Nagoya University, Nagoya, Japan; 134 INFN Sezione di Napoli, Napoli, Italy; 135 Dipartimento di Fisica, Università di Napoli, Napoli, Italy; 136 Department of Physics and Astronomy, University of New Mexico, Albuquerque, NM USA; 137 Institute for Mathematics, Astrophysics and Particle Physics, Radboud University Nijmegen/Nikhef, Nijmegen, The Netherlands; 138 Nikhef National Institute for Subatomic Physics and University of Amsterdam, Amsterdam, Netherlands; 139 Department of Physics, Northern Illinois University, DeKalb, IL USA; 140 Budker Institute of Nuclear Physics, SB RAS, Novosibirsk, Russia; 141 Department of Physics, New York University, New York, NY USA; 142 Ohio State University, Columbus, OH USA; 143 Faculty of Science, Okayama University, Okayama, Japan; 144 Homer L. Dodge Department of Physics and Astronomy, University of Oklahoma, Norman, OK USA; 145 Department of Physics, Oklahoma State University, Stillwater, OK USA; 146 Palacký University, RCPTM, Olomouc, Czech Republic; 147 Center for High Energy Physics, University of Oregon, Eugene, OR USA; 148 LAL, Univ. Paris-Sud, CNRS/IN2P3, Université Paris-Saclay, Orsay, France; 149 Graduate School of Science, Osaka University, Osaka, Japan; 150 Department of Physics, University of Oslo, Oslo, Norway; 151 Department of Physics, Oxford University, Oxford, UK; 152 INFN Sezione di Pavia, Pavia, Italy; 153 Dipartimento di Fisica, Università di Pavia, Pavia, Italy; 154 Department of Physics, University of Pennsylvania, Philadelphia, PA USA; 155 National Research Centre “Kurchatov Institute” B.P. Konstantinov Petersburg Nuclear Physics Institute, St. Petersburg, Russia; 156 INFN Sezione di Pisa, Pisa, Italy; 157 Dipartimento di Fisica E. Fermi, Università di Pisa, Pisa, Italy; 158 Department of Physics and Astronomy, University of Pittsburgh, Pittsburgh, PA USA; 159 Laboratório de Instrumentação e Física Experimental de Partículas-LIP, Lisbon, Portugal; 160 Faculdade de Ciências, Universidade de Lisboa, Lisbon, Portugal; 161 Department of Physics, University of Coimbra, Coimbra, Portugal; 162 Centro de Física Nuclear da Universidade de Lisboa, Lisbon, Portugal; 163 Departamento de Fisica, Universidade do Minho, Braga, Portugal; 164 Departamento de Fisica Teorica y del Cosmos and CAFPE, Universidad de Granada, Granada, Spain; 165 Dep Fisica and CEFITEC of Faculdade de Ciencias e Tecnologia, Universidade Nova de Lisboa, Caparica, Portugal; 166 Institute of Physics, Academy of Sciences of the Czech Republic, Praha, Czech Republic; 167 Czech Technical University in Prague, Praha, Czech Republic; 168 Charles University, Faculty of Mathematics and Physics, Prague, Czech Republic; 169 State Research Center Institute for High Energy Physics (Protvino), NRC KI, Protvino, Russia; 170 Particle Physics Department, Rutherford Appleton Laboratory, Didcot, UK; 171 INFN Sezione di Roma, Rome, Italy; 172 Dipartimento di Fisica, Sapienza Università di Roma, Rome, Italy; 173 INFN Sezione di Roma Tor Vergata, Rome, Italy; 174 Dipartimento di Fisica, Università di Roma Tor Vergata, Rome, Italy; 175 INFN Sezione di Roma Tre, Rome, Italy; 176 Dipartimento di Matematica e Fisica, Università Roma Tre, Rome, Italy; 177 Faculté des Sciences Ain Chock, Réseau Universitaire de Physique des Hautes Energies-Université Hassan II, Casablanca, Morocco; 178 Centre National de l’Energie des Sciences Techniques Nucleaires, Rabat, Morocco; 179 Faculté des Sciences Semlalia, Université Cadi Ayyad, LPHEA-Marrakech, Marrakech, Morocco; 180 Faculté des Sciences, Université Mohamed Premier and LPTPM, Oujda, Morocco; 181 Faculté des Sciences, Université Mohammed V, Rabat, Morocco; 182 DSM/IRFU (Institut de Recherches sur les Lois Fondamentales de l’Univers), CEA Saclay (Commissariat à l’Energie Atomique et aux Energies Alternatives), Gif-sur-Yvette, France; 183 Santa Cruz Institute for Particle Physics, University of California Santa Cruz, Santa Cruz, CA USA; 184 Department of Physics, University of Washington, Seattle, WA USA; 185 Department of Physics and Astronomy, University of Sheffield, Sheffield, UK; 186 Department of Physics, Shinshu University, Nagano, Japan; 187 Fachbereich Physik, Universität Siegen, Siegen, Germany; 188 Department of Physics, Simon Fraser University, Burnaby, BC Canada; 189 SLAC National Accelerator Laboratory, Stanford, CA USA; 190 Faculty of Mathematics, Physics and Informatics, Comenius University, Bratislava, Slovak Republic; 191 Department of Subnuclear Physics, Institute of Experimental Physics of the Slovak Academy of Sciences, Kosice, Slovak Republic; 192 Department of Physics, University of Cape Town, Cape Town, South Africa; 193 Department of Physics, University of Johannesburg, Johannesburg, South Africa; 194 School of Physics, University of the Witwatersrand, Johannesburg, South Africa; 195 Department of Physics, Stockholm University, Stockholm, Sweden; 196 The Oskar Klein Centre, Stockholm, Sweden; 197 Physics Department, Royal Institute of Technology, Stockholm, Sweden; 198 Departments of Physics and Astronomy and Chemistry, Stony Brook University, Stony Brook, NY USA; 199 Department of Physics and Astronomy, University of Sussex, Brighton, UK; 200 School of Physics, University of Sydney, Sydney, Australia; 201 Institute of Physics, Academia Sinica, Taipei, Taiwan; 202 Department of Physics, Technion: Israel Institute of Technology, Haifa, Israel; 203 Raymond and Beverly Sackler School of Physics and Astronomy, Tel Aviv University, Tel Aviv, Israel; 204 Department of Physics, Aristotle University of Thessaloniki, Thessaloníki, Greece; 205 International Center for Elementary Particle Physics and Department of Physics, The University of Tokyo, Tokyo, Japan; 206 Graduate School of Science and Technology, Tokyo Metropolitan University, Tokyo, Japan; 207 Department of Physics, Tokyo Institute of Technology, Tokyo, Japan; 208 Tomsk State University, Tomsk, Russia; 209 Department of Physics, University of Toronto, Toronto, ON Canada; 210 INFN-TIFPA, Trento, Italy; 211 University of Trento, Trento, Italy; 212 TRIUMF, Vancouver, BC Canada; 213 Department of Physics and Astronomy, York University, Toronto, ON Canada; 214 Faculty of Pure and Applied Sciences, and Center for Integrated Research in Fundamental Science and Engineering, University of Tsukuba, Tsukuba, Japan; 215 Department of Physics and Astronomy, Tufts University, Medford, MA USA; 216 Department of Physics and Astronomy, University of California Irvine, Irvine, CA USA; 217 INFN Gruppo Collegato di Udine, Sezione di Trieste, Udine, Italy; 218 ICTP, Trieste, Italy; 219 Dipartimento di Chimica, Fisica e Ambiente, Università di Udine, Udine, Italy; 220 Department of Physics and Astronomy, University of Uppsala, Uppsala, Sweden; 221 Department of Physics, University of Illinois, Urbana, IL USA; 222 Instituto de Fisica Corpuscular (IFIC) and Departamento de Fisica Atomica, Molecular y Nuclear and Departamento de Ingeniería Electrónica and Instituto de Microelectrónica de Barcelona (IMB-CNM), University of Valencia and CSIC, Valencia, Spain; 223 Department of Physics, University of British Columbia, Vancouver, BC Canada; 224 Department of Physics and Astronomy, University of Victoria, Victoria, BC Canada; 225 Department of Physics, University of Warwick, Coventry, UK; 226 Waseda University, Tokyo, Japan; 227 Department of Particle Physics, The Weizmann Institute of Science, Rehovot, Israel; 228 Department of Physics, University of Wisconsin, Madison, WI USA; 229 Fakultät für Physik und Astronomie, Julius-Maximilians-Universität, Würzburg, Germany; 230 Fakultät für Mathematik und Naturwissenschaften, Fachgruppe Physik, Bergische Universität Wuppertal, Wuppertal, Germany; 231 Department of Physics, Yale University, New Haven, CT USA; 232 Yerevan Physics Institute, Yerevan, Armenia; 233 Centre de Calcul de l’Institut National de Physique Nucléaire et de Physique des Particules (IN2P3), Villeurbanne, France; 234 CERN, 1211 Geneva 23, Switzerland

## Abstract

High-precision measurements by the ATLAS Collaboration are presented of inclusive $W^+ \rightarrow \ell ^+\nu$, $W^- \rightarrow \ell ^-\bar{\nu }$ and $Z/\gamma ^* \rightarrow \ell \ell$ ($\ell =e,\mu$) Drell–Yan production cross sections at the LHC. The data were collected in proton–proton collisions at $\sqrt{s} = 7\,\text {TeV}$ with an integrated luminosity of $4.6\,\mathrm {fb}^{-1}$. Differential $W^+$ and $W^-$ cross sections are measured in a lepton pseudorapidity range $|\eta _{\ell }|<2.5$. Differential $Z/\gamma ^*$ cross sections are measured as a function of the absolute dilepton rapidity, for $|y_{\ell \ell }| < 3.6$, for three intervals of dilepton mass, $m_{\ell \ell }$, extending from 46 to $150\,\,\text {GeV}$. The integrated and differential electron- and muon-channel cross sections are combined and compared to theoretical predictions using recent sets of parton distribution functions. The data, together with the final inclusive $e^{\pm }p$ scattering cross-section data from H1 and ZEUS, are interpreted in a next-to-next-to-leading-order QCD analysis, and a new set of parton distribution functions, ATLAS-epWZ16, is obtained. The ratio of strange-to-light sea-quark densities in the proton is determined more accurately than in previous determinations based on collider data only, and is established to be close to unity in the sensitivity range of the data. A new measurement of the CKM matrix element $\vert V_{cs} \vert$ is also provided.

## Introduction

The precise measurement of inclusive $W^+$, $W^-$ and $Z/\gamma ^*$ production in *pp* scattering at the LHC constitutes a sensitive test of perturbative quantum chromodynamics (QCD). The rapidity dependence of boson production in the Drell–Yan process provides constraints on the parton distribution functions (PDFs) of the proton, as the boson rapidity is strongly correlated with the proton momentum fractions $x_1$, $x_2$ carried by the partons participating in the hard scattering subprocess. The weak and electromagnetic components of the neutral current (NC) process, $Z/\gamma ^* \rightarrow \ell \ell$, combined with the weak charged current (CC) reactions, $W^+ \rightarrow \ell ^+\nu$ and $W^- \rightarrow \ell ^-\bar{\nu }$, probe the quark flavours of the proton in a way that complements the information from deep inelastic lepton–hadron scattering (DIS).

The previous differential $W,\,Z$ cross-section measurement of ATLAS [1] at a centre-of-mass energy of $\sqrt{s}=7\,\text {TeV}$ was based on a data sample taken in 2010 with an integrated luminosity of $36\,\mathrm {pb}^{-1}$, determined with an uncertainty of 3.5%. The precision of that measurement – not including the luminosity uncertainty – reached about 2–3%. The new $W^{\pm },~Z$ cross-section measurement presented here uses the data taken at $\sqrt{s}=7\,\text {TeV}$ by ATLAS in 2011. This data sample has a hundred times more integrated luminosity, $4.6\,\mathrm {fb}^{-1}$, measured with an improved precision of 1.8% [2]. A deeper understanding of detector performance and refined analysis techniques are crucial to reach a measurement precision at the sub-percent level, apart from the luminosity uncertainty.

Compared to the previous analysis [1], in this article the NC measurement range is extended to values of dilepton mass, $m_{\ell \ell }$ , significantly below and above the *Z* peak, covering the range $46< m_{\ell \ell }< 150\,\text {GeV}$. ATLAS NC data have also been presented at even lower [3] ($12< m_{\ell \ell }< 66\,\text {GeV}$) and higher dilepton masses [4, 5] ($116< m_{\ell \ell }< 1500\,\text {GeV}$). Precise NC measurements at $\sqrt{s}=8\,\text {TeV}$ over a range of dilepton masses of $12<m_{\ell \ell }<150\,\,\text {GeV}$ focused on boson transverse momentum distributions have been provided in Ref. [6]. Recently, first integrated cross-section results on inclusive $W^{\pm }$ and *Z* production at $\sqrt{s}=13\,\text {TeV}$ were published by ATLAS [7].

Weak boson cross-section measurements at forward rapidity were presented by LHCb [8–15] in the muon and electron channels. The CMS Collaboration has measured NC cross sections as a function of boson mass and rapidity [16, 17], of boson transverse momentum and rapidity [18], as well as differential $W^\pm$ charge asymmetries [19–21], and integrated *W* and *Z* cross sections [22, 23].

The precision of the present measurement of the $W^\pm$ and $Z/\gamma ^*$ cross sections exceeds that of the previous related measurements. The analysis is performed in both the electron channels, $W^{\pm } \rightarrow e \nu$ and $Z/\gamma ^* \rightarrow e^+e^-$, and the muon channels, $W^{\pm } \rightarrow \mu \nu$ and $Z/\gamma ^* \rightarrow \mu ^+\mu ^-$, in a common fiducial phase space. These measurements provide a new sensitive test of electron–muon universality in the weak interaction sector. The electron and muon data are combined, accounting for all correlations of systematic uncertainties.

Cross-section calculations of the Drell–Yan process are available at up to next-to-next-to-leading order in the strong coupling constant $\alpha _{\text {S}}$ (NNLO QCD) and up to next-to-leading order for electroweak effects (NLO electroweak). The NNLO QCD predictions are calculated with kinematic requirements applied to match the detector acceptance using the DYNNLO [24, 25] and FEWZ [26–28] programs. The NLO electroweak corrections are an important ingredient at this level of precision and can be evaluated with FEWZ for the NC processes and with the SANC programs [29] for both NC and CC processes. The measured integrated and differential cross sections are compared to calculations using various recent PDF sets: ABM12 [30], CT14 [31], HERAPDF2.0 [32], JR14 [33], MMHT14 [34], and NNPDF3.0 [35]. A quantitative analysis within a profiling procedure [36, 37] is presented to test the compatibility of the new $W,\, Z$ cross-section data with theoretical predictions using these PDF sets, and to illustrate the impact of the data on PDF determinations.

The previous ATLAS $W,\,Z$ cross-section measurement [1] and its QCD interpretation [38] suggested that the light quark sea ($u,\, d,\, s$) is flavour symmetric, i.e. the ratio of the strange-to-anti-down quark densities, $r_s=(s+\bar{s})/2\bar{d}$, was found to be close to unity at $x \simeq 0.023$ within an experimental uncertainty of about 20%. This is re-examined here in a new QCD fit analysis using the present ATLAS measurement together with the final, combined NC and CC DIS cross-section data from the H1 and ZEUS experiments at the HERA collider [32]. The analysis provides a new NNLO PDF set, ATLAS-epWZ16, superseding the ATLAS-epWZ12 set [38]. It also allows the magnitude of the CKM matrix element $\vert V_{cs} \vert$ to be determined, without assuming unitarity of the CKM matrix, with a precision comparable to the determinations from charm hadron decays [39].

The paper is organized as follows. Section 2 presents the detector, data and simulated event samples and cross-section as well as kinematic definitions. The measurements, of both the $W^{\pm }$ and the $Z/\gamma ^*$ reactions, are performed independently for the electron and muon decay channels as described in Sects. 3 and 4. The cross-section results are presented in Sect. 5, which contains the analysis method, a test of electron–muon universality, and a description of the procedure for, and results of, combining the electron and the muon data. In Sect. 6 the integrated and differential cross sections are compared with theoretical calculations using recent NNLO PDF sets. Measurements are also presented of the $W^{\pm }$ charge asymmetry and various other cross-section ratios. This section concludes with the results of the PDF profiling analysis. Finally, Sect. 7 presents an NNLO QCD fit analysis of the present ATLAS data and the final HERA NC and CC DIS cross-section data, resulting in an improved determination of the strange-quark distribution in the proton and a measurement of $\vert V_{cs} \vert$. A summary of the paper is presented in Sect. 8.

## Detector, simulation and definitions

### Detector and data samples

The ATLAS detector [40] comprises a superconducting solenoid surrounding the inner detector (ID) and a large superconducting toroid magnet system with muon detectors enclosing the calorimeters. The ID system is immersed in a 2 T axial magnetic field and provides tracking information for charged particles in a pseudorapidity range matched by the precision measurements of the electromagnetic calorimeter. The inner silicon pixel and strip tracking detectors cover the pseudorapidity range $|\eta |< 2.5$.^1^ The transition radiation tracker, surrounding the silicon detectors, contributes to the tracking and electron identification for $|\eta | < 2.0$.

The liquid argon (LAr) electromagnetic (EM) calorimeter is divided into one barrel ($|\eta | < 1.475$) and two end-cap components ($1.375< |\eta | < 3.2$). It uses lead absorbers and has an accordion geometry to ensure a fast and uniform response and fine segmentation for optimal reconstruction and identification of electrons and photons. The hadronic steel/scintillator-tile calorimeter consists of a barrel covering the region $|\eta | < 1.0$, and two extended barrels in the range $0.8< |\eta | < 1.7$. The copper/LAr hadronic end-cap calorimeter ($1.5<|\eta |<3.2$) is located behind the electromagnetic end-cap calorimeter. The forward calorimeter (FCAL) covers the range $3.2< |\eta | < 4.9$ and also uses LAr as the active material and copper or tungsten absorbers for the EM and hadronic sections, respectively.

The muon spectrometer (MS) is based on three large superconducting toroids with coils arranged in an eight-fold symmetry around the calorimeters, covering a range of $|\eta |<2.7$. Over most of the $\eta$ range, precision measurements of the track coordinates in the principal bending direction of the magnetic field are provided by monitored drift tubes. At large pseudorapidities ($2.0< |\eta | < 2.7$), cathode strip chambers with higher granularity are used in the layer closest to the IP. The muon trigger detectors consist of resistive plate chambers in the barrel ($|\eta |<1.05$) and thin gap chambers in the end-cap regions ($1.05< |\eta | < 2.4$), with a small overlap around $|\eta | \simeq 1.05$.

In 2011, the ATLAS detector had a three-level trigger system consisting of Level-1 (L1), Level-2 (L2) and the Event Filter (EF). The L1 trigger rate was approximately 75 kHz. The L2 and EF triggers reduced the event rate to approximately 300 Hz before data transfer to mass storage.

The data for this analysis were collected by the ATLAS Collaboration during 2011, the final year of operation at $\sqrt{s}=7\,\text {TeV}$. The analysis uses a total luminosity of $4.6\,\mathrm {fb}^{-1}$ with an estimated uncertainty of $1.8\%$ [2], where the main components of the apparatus were operational. Data and simulated event samples were processed with common reconstruction software.

### Simulated event samples

Simulated and reconstructed Monte Carlo (MC) samples are used to model the properties of signals and background processes and to calculate acceptance and efficiency corrections for the extraction of cross sections. Dedicated efficiency and calibration studies with data are used to derive correction factors to account for the small differences between experiment and simulation, as is subsequently described.

The main signal event samples for $W^{\pm } \rightarrow \ell \nu$ and $Z/\gamma ^* \rightarrow \ell \ell$ production are generated using the Powheg [41–44] event generator, with the simulation of parton showers, hadronization and underlying events provided by Pythia6 [45]. Systematic uncertainties in the measurements due to imperfect modelling of the signals are estimated with alternative event samples generated with Powheg interfaced instead to the Herwig [46] and Jimmy [47] programs (referred to later as the Powheg+Herwig sample) as well as MC@NLO [48], also interfaced to the Herwig and Jimmy programs (referred to later as the MC@NLO+Herwig sample). For the MC@NLO and Powheg matrix element calculations the CT10 NLO PDF [49] set is used, whereas showering is performed with CTEQ6L1 [50]. Samples of $W \rightarrow \tau \nu$ and $Z/\gamma ^* \rightarrow \tau ^+\tau ^-$ events are generated with the Alpgen generator [51] interfaced to Herwig and Jimmy and using the CTEQ6L1 PDF set, and also Powheg interfaced to Pythia8 [52].

All simulated samples of $W^{\pm } \rightarrow \ell \nu$ and $Z/\gamma ^* \rightarrow \ell \ell$ production are normalized to the NNLO cross sections calculated by the FEWZ program with the MSTW2008 NNLO PDF set [53]. When employing these samples for background subtraction, an uncertainty in the total cross section of 5% is assigned to account for any uncertainties arising from the PDFs as well as factorization-scale and renormalization-scale uncertainties. As the simulated transverse momentum spectrum of the $W^{\pm }$ and $Z/\gamma ^*$ bosons does not describe the one observed in data well, all samples are reweighted by default to the Powheg+Pythia8 AZNLO prediction [54], which describes the $Z \rightarrow \ell \ell$ data well at low and medium dilepton transverse momentum $p_{\mathrm {T},\ell \ell } < 50\,\,\text {GeV}$.

Top-quark pair ($t\bar{t}$) and single top-quark production are simulated with MC@NLO interfaced to Herwig and Jimmy. The $t\bar{t}$ cross section is calculated at a top quark mass of $172.5\,\,\text {GeV}$ at NNLO in QCD including resummation of next-to-next-to-leading logarithmic soft-gluon terms (NNLL) with top++2.0 [55–60]. The total theoretical uncertainty of the $t\bar{t}$ production cross section is calculated using the PDF4LHC prescription [61] using the MSTW2008 NNLO [53], CT10 NNLO [62] and NNPDF2.3 5f FFN [63] PDF sets and adding in quadrature the scale and $\alpha _{\text {S}}$ uncertainties. The single-top-quark cross sections are calculated at approximate NNLO+NNLL accuracy [64–67].

Inclusive production of dibosons *WW*, *WZ* and *ZZ* is simulated with Herwig. The samples are normalized to their respective NLO QCD cross sections [68] with 6% uncertainty.

While most studies of the multijet background are performed using control samples from data, some studies in the muon channels are carried out with Pythia6 samples, where inclusive, heavy-flavour dijet production ($c\bar{c}$ and $b\bar{b}$) is simulated and the samples are filtered for high-$p_{\text {T}}$ muons from charm or bottom hadron decays.

All generators are interfaced to Photos [69] to simulate the effect of final-state QED radiation (QED FSR). The decays of $\tau$ leptons in Herwig and Pythia6 samples are handled by Tauola [70]. The passage of particles through the ATLAS detector is modelled [71] using GEANT4 [72]. The effect of multiple *pp* interactions per bunch crossing (“pile-up”) is modelled by overlaying the hard-scattering event with additional simulated inelastic collision events following the distribution observed in the data with about nine simultaneous inelastic interactions on average. These events are simulated using Pythia6 with the AMBT2 tune [73]. While the simulation of pile-up events reproduces the observed width of the luminous region along the beam direction, a reweighting is applied to match the longitudinal distribution of the hard-scatter vertex to that observed in the data. This is needed to accurately control acceptance and detector effects, which depend on the details of the detector geometry.

### Cross-section definition and fiducial regions

The measurements reported here correspond to inclusive Drell–Yan cross sections with a direct decay of the intermediate boson, $Z/\gamma ^* \rightarrow \ell \ell$ or $W \rightarrow \ell \nu$, where $\ell =e$ or $\mu$. Other processes that may lead to a pair of leptons, $\ell \ell$ or $\ell \nu$, in the final state are subtracted as background. These are $t\bar{t}$ pair and single top-quark production, cascade decays $Z/\gamma ^* \rightarrow \tau ^+\tau ^-\rightarrow \ell ^+\ell ^- X$ and $W \rightarrow \tau \nu \rightarrow \ell \nu X$, photon-induced lepton-pair production $\gamma \gamma \rightarrow \ell \ell$, and gauge boson pair production, with both boson masses exceeding $20\,\,\text {GeV}$. Experimental contaminations of signals through other channels, such as $Z/\gamma ^* \rightarrow \ell \ell$ contributing as background to $W^{\pm }$ or the small, opposite-sign $W^{\mp }$ fraction in the $W^{\pm }$ selections, are corrected for as well.

Each channel of the measurement covers somewhat different regions of phase space. For electrons this corresponds to a restriction to $|\eta _{\ell }|<2.47$ for central electrons, and further the exclusion of the regions $1.37<|\eta _{\ell }|<1.52$ and $3.16<|\eta _{\ell }|<3.35$. For muons the acceptance is restricted to $|\eta _{\ell }|<2.4$.

The combined $e-\mu$ cross sections are reported in common fiducial regions close to the initial experimental selections so as to involve only minimal extrapolations. The kinematic requirements applied for the cross-section measurements are as follows:

$$\begin{aligned} \text{ Central }\ Z/\gamma ^* \rightarrow \ell \ell :&p_{\mathrm {T}, \ell }> 20\,\,\text {GeV},\ |\eta _{\ell }|<2.5,\ 46< m_{\ell \ell }< 150\,\,\text {GeV}\\ \text{ Forward }\ Z/\gamma ^* \rightarrow \ell \ell :&p_{\mathrm {T}, \ell }> 20\,\,\text {GeV},\ \text{ one } \text{ lepton }\ |\eta _{\ell }|<2.5,\ \text{ other } \text{ lepton }\ 2.5<|\eta _{\ell }|<4.9,\\&\ 66< m_{\ell \ell }< 150\,\,\text {GeV}\\ W^{\pm } \rightarrow \ell \nu \ :&p_{\mathrm {T}, \ell }> 25\,\,\text {GeV},\ |\eta _{\ell }|<2.5,\ p_{\mathrm {T}, \nu }>25\,\,\text {GeV},\ m_\mathrm {T}> 40\,\,\text {GeV}. \end{aligned}$$

Here the charged-lepton transverse momentum and pseudorapidity are denoted by $p_{\mathrm {T}, \ell }$ and $\eta _{\ell }$, respectively. The transverse momentum of the neutrino is given by $p_{\mathrm {T}, \nu }$ and the *W*-boson transverse mass is calculated as $m_\mathrm {T}^2 = 2\,p_{\mathrm {T}, \ell }\,p_{\mathrm {T}, \nu }\,[1-\cos (\Delta \phi _{\ell ,\nu })]$, where $\Delta \phi _{\ell ,\nu }$ is the azimuthal angle between the charged lepton and the neutrino directions. The lepton kinematics used in the definition of the cross sections corresponds to the Born level for QED final-state radiation effects. These fiducial regions differ slightly from those used in Ref. [1] such that the corresponding cross-section results cannot be compared directly.

The integrated charged-current fiducial cross sections are presented separately for $W^+$, $W^-$ and their sum. Integrated neutral-current fiducial cross sections are presented for the *Z*-peak region, corresponding to $66< m_{\ell \ell }< 116\,\text {GeV}$, where they are most precise.

The differential $W^{\pm } \rightarrow \ell \nu$ cross sections are measured as a function of the absolute values of the charged-lepton pseudorapidity, $\eta _{\ell }$, in bins with boundaries given by

1 $$\begin{aligned}&|\eta _{\ell }| = [0.00,\,0.21,\,0.42,\,0.63,\,0.84,\,1.05,\,1.37, \,1.52,\nonumber \\&\qquad \qquad 1.74,\,1.95,\,2.18,\,2.50]. \end{aligned}$$

The differential $Z/\gamma ^*$ cross sections are presented as a function of dilepton rapidity, $y_{\ell \ell }$, in three intervals of dilepton mass, $m_{\ell \ell }$, with bin edges

2 $$\begin{aligned} m_{\ell \ell }= [46,\,66,\,116,\,150]\,\,\text {GeV}. \end{aligned}$$

In the *Z*-peak region, the boundaries of the bins in dilepton rapidity $y_{\ell \ell }$ are chosen to be

3 $$\begin{aligned}&|y_{\ell \ell }| = [0.0,\,0.2,\,0.4,\,0.6,\,0.8,\,1.0,\,1.2,\,1.4,\,1.6,\nonumber \\&\qquad \qquad \, 1.8,\,2.0,\,2.2,\,2.4], \end{aligned}$$

while in the adjacent mass intervals, below and above the *Z* peak, the binning is twice as coarse and ranges also from $|y_{\ell \ell }|=0$ to 2.4.

A dedicated $Z/\gamma ^* \rightarrow \ell \ell$ analysis in the electron channel extends into the forward region of $y_{\ell \ell }$, covering the range from $|y_{\ell \ell }| =1.2$ to 3.6. This analysis is only performed in the two higher mass intervals, with the boundaries $m_{\ell \ell }= [66,\,116,\,150]\,\text {GeV}$, as the region below $m_{\ell \ell }<66\,\text {GeV}$ cannot be measured with good precision with the current lepton $p_{\text {T}}$ acceptance in this channel. In the *Z*-peak region of the forward $Z/\gamma ^*$ analysis the boundaries of the bins in dilepton rapidity $y_{\ell \ell }$ are chosen as

4 $$\begin{aligned} |y_{\ell \ell }| = [1.2,\,1.4,\,1.6,\,1.8,\,2.0,\,2.2,\,2.4, \,2.8,\,3.2,\,3.6], \end{aligned}$$

while for the higher mass interval the same range is divided into six bins of equal size.

## Electron channel measurements

### Event selection

Events are required to have at least one primary vertex formed by at least three tracks of $p_{\text {T}} >500\,\text {MeV}$. If multiple vertices are reconstructed, the one with the highest sum of squared transverse momenta of associated tracks, $\sum p_{\text {T}}^{2}$, is selected as the primary vertex.

Central electron candidates are reconstructed from an ID track matched to an energy deposit in the EM calorimeter [74]. They are required to be within the coverage of the ID and the precision region of the EM calorimeter, $|\eta |< 2.47$. The transition region between the barrel and end-cap calorimeters, $1.37<|\eta |<1.52$, is excluded, as the reconstruction quality is significantly reduced compared to the rest of the pseudorapidity range. The electron momentum vector is calculated by combining the calorimeter measurement of the energy and the tracker information on the direction. The electron is required to satisfy “tight” identification criteria [74] based on the shower shapes of the cluster of energy in the calorimeter, the track properties, and the track-to-cluster matching. The combined efficiency for electrons from *W* and *Z* decays to be reconstructed and to meet these “tight” identification criteria depends strongly on both $\eta$ and $p_{\text {T}}$. In the most central region of the detector, at $|\eta |<0.8$, this efficiency is about 65% at $p_{\text {T}} =20\,\,\text {GeV}$ and increases to about 80% at $p_{\text {T}} =50\,\,\text {GeV}$. In the more forward region, $2.0<|\eta |<2.47$, the corresponding efficiencies are in the range 50–75% for transverse momenta $p_{\text {T}} = 20$–$50\,\,\text {GeV}$.

The same “tight” requirements are imposed for all central electron candidates to enable a coherent treatment across all $W^\pm$ and $Z/\gamma ^*$ analyses, even though the background rejection is less crucial for the $Z/\gamma ^*$ analysis with two central electrons. To improve the rejection of background from non-isolated electrons, converted photons, or hadrons misidentified as electrons, isolation criteria are imposed on the electron candidates in the $W \rightarrow e \nu$ and forward $Z/\gamma ^* \rightarrow e^+e^-$ analyses. The isolation of central electron candidates in these channels is implemented by setting an upper limit on both the energy measured in the calorimeter in a cone of size $\Delta R = 0.2$ around the electron cluster and the sum of transverse momenta of all tracks in a cone of size $\Delta R = 0.4$ around the trajectory of the electron candidate. The contribution from the electron candidate itself is excluded in both cases. The specific criteria are optimized as a function of electron $\eta$ and $p_{\text {T}}$ to have a combined efficiency of about 95% in the simulation for isolated electrons from the decay of a *W* or *Z* boson.

Forward electron candidates are reconstructed in the region $2.5< |\eta | < 4.9$, excluding the transition region between the end-cap and the FCAL calorimeter, 3.16$<|\eta |<$3.35, and are required to satisfy “forward tight” identification requirements with a typical efficiency in the range of 65–85% [74]. As the forward region is not covered by the ID, the electron identification has to rely on calorimeter cluster shapes only. The forward electron momentum is determined from the calorimeter cluster energy and position.

In an inclusive $W \rightarrow \ell \nu$ analysis, signal events can be considered to consist of three contributions: the isolated charged lepton, the undetected neutrino, and any further particles produced in the hadronization of quarks and gluons produced in association with the *W* boson. This last contribution is referred to as the hadronic recoil [75]. The missing transverse momentum, $E_{\text {T}}^{\text {miss}}$, is given by the negative vectorial sum of the transverse momentum components of the charged lepton and the hadronic recoil and identified with the undetected neutrino. The $E_{\text {T}}^{\text {miss}}$ is reconstructed from energy deposits in the calorimeters and muons reconstructed in the MS [76, 77]. Calorimeter energy deposits associated to an electron candidate meeting the “medium” identification criteria [74] and exceeding $p_{\text {T}} >10\,\,\text {GeV}$ are calibrated to the electron scale. Alternatively, if calorimeter energy deposits can be associated to a jet reconstructed with the anti-$k_t$ algorithm with radius parameter $R=0.6$ and $p_{\text {T}} >20 \,\text {GeV}$, the calibrated jet is used [78]. Finally, identified combined and isolated muons, as described in Sect. 4, with $p_{\text {T}} >10\,\,\text {GeV}$, are used in the $E_{\text {T}}^{\text {miss}}$ reconstruction, removing the energy deposits of such muons in the calorimeter. Any remaining energy deposits in the calorimeters are added to the $E_{\text {T}}^{\text {miss}}$ after calibration with the local hadronic calibration [78].

During data collection, events with one central electron were selected with a single-electron trigger with “medium” identification criteria and a $p_{\text {T}}$ threshold of 20 or $22\,\text {GeV}$ [79]. The rise in threshold was enforced by the increasing instantaneous luminosity delivered by the LHC during 2011. Events with two central electrons are furthermore selected online by a dielectron trigger in which two electrons are required to satisfy the “medium” identification criteria and a lower $p_{\text {T}}$ threshold of $12\,\text {GeV}$.

To select *W*-boson events in the electron channel, exactly one central identified and isolated electron is required with a transverse momentum $p_{\text {T}} > 25\,\text {GeV}$. This electron is also required to have passed the single-electron trigger. Events with at least one additional central electron meeting the “medium” identification criteria [74] and $p_{\text {T}} > 20\,\,\text {GeV}$ are rejected to reduce background from $Z/\gamma ^* \rightarrow e^+e^-$ events. The missing transverse momentum is required to exceed $E_{\text {T}}^{\text {miss}} = 25\,\text {GeV}$ and the transverse mass of the electron–$E_{\text {T}}^{\text {miss}}$ system, $m_\mathrm {T}$, has to be larger than $40\,\text {GeV}$.

The selection for the central $Z/\gamma ^* \rightarrow e^+e^-$ analysis requires exactly two identified electrons with $p_{\text {T}} >20\,\,\text {GeV}$. These two electrons must have passed the dielectron trigger selection. No requirement is made on the charge of the two electron candidates. The analysis examines the invariant mass $m_{ee}$ interval from 46 to $150\,\text {GeV}$.

For the selection of forward $Z/\gamma ^* \rightarrow e^+e^-$ events over an extended range of rapidity, a central identified and isolated electron is required as in the $W \rightarrow e \nu$ channel, but lowering the transverse momentum threshold to the minimum $p_{\text {T}} = 23\,\text {GeV}$ accessible with the single-electron trigger. A second electron candidate with $p_{\text {T}} > 20\,\text {GeV}$ has to be reconstructed in the forward region. The invariant mass of the selected pair is required to be between 66 and $150\,\text {GeV}$.

### Calibration and efficiencies

Comprehensive evaluations of the reconstruction of electrons are described in Refs. [74, 80]. The energy of the electron is calibrated using a multivariate algorithm trained on simulated samples of single electrons to achieve an optimal response and resolution. Residual corrections to the energy scale and resolution are determined from data as a function of $\eta$ in the central and forward regions by comparing the measured $Z \rightarrow e^+e^-$ line shape to the one predicted by the simulation [80]. The energy-scale corrections applied to the data are typically within a range of ${\pm }$2% and the systematic uncertainty of the energy scale is typically $0.1\%$. Resolution corrections of around $(1.0 \pm 0.3)\%$ are applied to the simulation to match the data, where the quoted uncertainty corresponds to the precision of the correction.

The electron efficiencies are controlled in several steps corresponding to the reconstruction and identification of electron candidates as well as the isolation and trigger requirements described above. All central electron efficiencies are measured as a function of the electron pseudorapidity and electron transverse momentum, while in the forward region $2.5<|\eta |<4.9$ the corrections are binned in electron pseudorapidity only. All uncertainties in the electron efficiency measurements are classified as being of statistical or systematic origin, where the latter has components correlated and uncorrelated across $\eta$ and $p_{\text {T}}$  [74]. This classification allows the corresponding systematics to be propagated correctly to the final measurement as described in Sect. 5.4.

The efficiencies for electrons from *W* or *Z* decays in the central region to satisfy the “tight” identification requirements are measured using two different tag-and-probe methods performed with *W* and *Z* data samples [74]. The data-to-simulation ratios of the efficiencies measured in these two samples are combined. They are typically within $\pm 0.05$ of unity with significant variations as a function of pseudorapidity. The total uncertainty in these factors is 0.5–1.0%.

The central electron trigger, reconstruction and isolation efficiencies as well as the forward electron identification efficiencies are determined using the *Z* tag-and-probe method only. Corresponding correction factors are derived in all cases and applied to the simulation. The efficiencies for the reconstruction of central electrons are measured with a precision of mostly better than 0.5% and are found to be described by the simulation within typically ${\pm } 1\%$. The efficiency of the electron isolation requirement employed in the $W \rightarrow e \nu$ and forward $Z/\gamma ^* \rightarrow e^+e^-$ analysis is well described by the simulation within ${\pm } 1\%$ variations and the corresponding correction factors have typically ${<}0.3\%$ uncertainty. The electron trigger efficiencies are measured separately for the single-electron and dielectron triggers and for various different configurations employed during the data-taking. Most data-to-simulation correction factors for the trigger selection are within ${\pm } 1\%$ of unity and determined with a precision of better than 1%.

The forward electron reconstruction efficiency has been found to be nearly 100% in the simulation. The identification efficiencies are found to be lower in data than in the simulation by about 10% and are measured with a precision of 3–8%.

The distinction between $W^+$ and $W^-$ events relies on the measurement of the charge of the decay electron. The charge misidentification probability as a function of $\eta$ is determined in both data and simulation from the fraction of $Z \rightarrow e^+e^-$ events where both electrons are reconstructed with the same sign. It depends on the identification criteria and in general increases at large $|\eta |$ [74]. A correction is applied to the simulation to match the rate observed in the data. In the $Z/\gamma ^* \rightarrow e^+e^-$ analysis, the majority of dielectron events reconstructed with same charge, with an invariant mass close to the *Z*-boson mass and satisfying the identification requirements, are indeed signal events. The efficiency loss of an opposite-charge selection through charge misidentification of either electron incurs a non-negligible systematic uncertainty, which is avoided by not applying the opposite-charge selection in the $Z/\gamma ^* \rightarrow e^+e^-$ analysis.

Uncertainties in the $E_{\text {T}}^{\text {miss}}$ scale and resolution are determined by the corresponding uncertainties for the electrons [80], muons [81], and jets [78] used in the reconstruction. The uncertainties in the remaining “soft” part are evaluated by reconstructing the hadronic recoil in $Z \rightarrow \ell \ell$ events and comparing the recoil response to the dilepton system in both data and simulation [77].

### Backgrounds

The backgrounds contributing in the $W \rightarrow e \nu$ channel can be divided into two categories: (1) electroweak background processes and top-quark production, which are estimated using MC prediction, and (2) background from multijet production determined with data-driven methods.

The largest electroweak background in the $W \rightarrow e \nu$ channel is due to the $W \rightarrow \tau \nu$ production where isolated electrons are produced in the decay $\tau \rightarrow e\bar{\nu }\nu$. Relative to the number of all $W^\pm$ candidate events, this contribution is estimated to be between 1.6 and 1.9% for the different bins of the pseudorapidity with a similar fraction in $W^+$ and $W^-$ events. The contamination of the $W \rightarrow e \nu$ sample by $Z/\gamma ^* \rightarrow e^+e^-$ is determined to be between 0.7 and 1.3%. Further contributions, at the 0.1–0.5% level, arise from $t\bar{t}$, $Z/\gamma ^* \rightarrow \tau ^+\tau ^-$, single top-quark and diboson production. The sum of electroweak and top-quark backgrounds is between 3.3 and 3.9% in the $W^-$ channel and between 2.8 and 3.5% in the $W^+$ channel. In contrast to the $W \rightarrow \tau \nu$ background, the other electroweak and top-quark background yields are of similar absolute size in $W^+$ and $W^-$ channels.

Multijet production from QCD processes is a significant source of background in the $W \rightarrow e \nu$ channel when non-isolated electrons, converted photons or hadrons are misidentified as isolated electrons and neutrinos from hadron decays or resolution effects cause a significant measurement of missing transverse momentum in the event. This background is estimated from the data using a template fit of the $E_{\text {T}}^{\text {miss}}$ distribution in a normalization region that differs from the signal region by relaxed the $E_{\text {T}}^{\text {miss}}$ and $m_\mathrm {T}$ requirements. A template to represent the multijet background contribution is selected from data using the same kinematic requirements as for signal electrons, but inverting a subset of the electron identification criteria and requiring the electron candidate not to be isolated. The isolation is estimated from the energy deposited in the calorimeter in a cone of size $\Delta R = 0.3$ around the electron candidate, denoted by $E_\mathrm {T}^\mathrm {cone30}$, and the condition $E_\mathrm {T}^\mathrm {cone30}/p_{\text {T}} >0.20$ is imposed. A second template that combines the $W \rightarrow e \nu$ signal and electroweak and top-quark contributions is taken from the simulation.

**Fig. 1:**
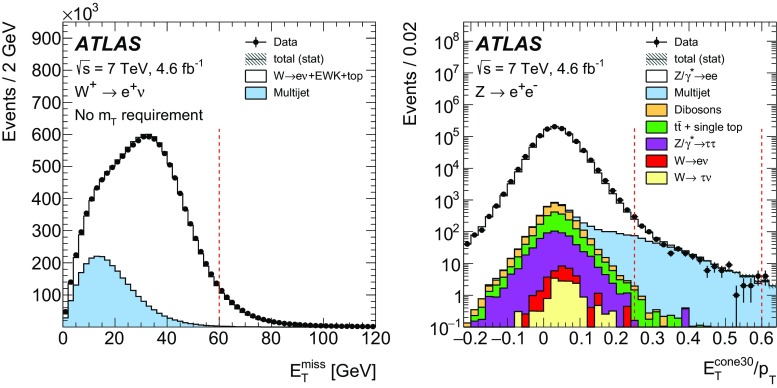
Distributions used for the estimation of the multijet background in the $W^+ \rightarrow e^+ \nu$ channel (*left*) and $Z \rightarrow e^+e^-$ channel (*right*). For the $W^+ \rightarrow e^+ \nu$ channel, the result of the template fit in a multijet-enhanced region using the $E_{\text {T}}^{\text {miss}}$ distribution is shown. The *vertical line* indicates the upper boundary ($E_{\text {T}}^{\text {miss}} = 60\,\,\text {GeV}$) of the region used in the fit. The label “EWK+top” refers to the electroweak and top-quark background contributions estimated from MC simulation, which are here treated in a common template together with the $W \rightarrow e \nu$ signal. In the $Z \rightarrow e^+e^-$ channel, the region of large isolation $E_\mathrm {T}^\mathrm {cone30}/p_{\text {T}}$, between the *two vertical lines*, is used to normalize the multijet template from data. The shown distribution is taken from the central $Z \rightarrow e^+e^-$ analysis in the region $66<m_{ee}<116\,\,\text {GeV}$. The sum of all expected background and signal contributions is shown as a *solid line* with a *hashed band* detailing the statistical uncertainty and labelled “total (stat)”

The relative fraction of the two components is determined by a fit to the data in the normalization region. The normalization region contains the signal region to constrain the signal contribution, relaxes the lower $E_{\text {T}}^{\text {miss}}$ and $m_\mathrm {T}$ requirements to increase the multijet fraction and furthermore imposes $E_{\text {T}}^{\text {miss}} <60\,\,\text {GeV}$ to avoid a mismodelling of the high $E_{\text {T}}^{\text {miss}}$ region, which was established in a study of the $Z \rightarrow e^+e^-$ sample. No prior knowledge of either template’s normalization is assumed, and the fit is performed separately for the $W^+$ and $W^-$ channels and also in each bin of electron pseudorapidity to obtain the background for the differential analysis. The resulting $E_{\text {T}}^{\text {miss}}$ distribution for the case of the inclusive $W^+$ selection is shown in the left panel of Fig. 1. The background in the signal region $E_{\text {T}}^{\text {miss}} > 25\,\,\text {GeV}$ and $m_\mathrm {T}>40\,\,\text {GeV}$ is then obtained by multiplying the multijet yield determined in the fit by the fraction of events in the template sample that satisfy the signal region and normalization region $E_{\text {T}}^{\text {miss}}$ and $m_\mathrm {T}$ requirements, respectively. This multijet estimate is found to change in a systematic way when the $E_{\text {T}}^{\text {miss}}$ and $m_\mathrm {T}$ requirements imposed for the normalization region are progressively tightened to resemble more the $E_{\text {T}}^{\text {miss}}$ and $m_\mathrm {T}$ requirements of the signal region. This dependence is measured and linearly extrapolated to the point where the normalization region has the same $E_{\text {T}}^{\text {miss}}$ and $m_\mathrm {T}$ thresholds as the signal region. A corresponding correction of typically 10% is applied to obtain an improved multijet estimate, while the full size of this correction is assigned as a systematic uncertainty. Further systematic uncertainties are derived from variations of the background and signal template shapes. Background shape uncertainties are obtained from varied template selection criteria by changing the $E_\mathrm {T}^\mathrm {cone30}/p_{\text {T}}$ selection, requiring the electron-candidate track to have a hit in the innermost layer of the ID, or changing the subset of identification criteria that the electron is allowed to not satisfy from the “tight” to the “medium” identification level. The shape uncertainties on the signal template from the detector systematic uncertainties discussed in Sect. 3.2 and using the alternative signal MC simulation samples discussed in Sect. 2.2 are considered as well.

The multijet background in the signal region ranges from 2.1% in the most central pseudorapidity bin to 6.9% in the most forward bin of the measurement for the $W^+$ and from 2.8 to 11% for the $W^-$ channel respectively. The total systematic uncertainty is at the level of 15–25% and the statistical uncertainty is typically a factor of ten smaller. While this background is determined separately for $W^+$ and $W^-$ samples, the resulting background yields for the two charges are found to be compatible within their statistical uncertainties. An alternative method for the determination of the multijet fractions, following Ref. [7], gives an estimate well within the systematic uncertainty assigned to the baseline determination described above.

In the central $Z/\gamma ^* \rightarrow e^+e^-$ analysis, the relative background contributions due to electroweak processes with two isolated electrons, from $Z/\gamma ^* \rightarrow \tau ^+\tau ^-$, $t\bar{t}$, single top-quark, and diboson production are estimated using the corresponding MC samples. That background is dominated by the $Z/\gamma ^* \rightarrow \tau ^+\tau ^-$ process below the *Z* peak and the $t\bar{t}$ process above the *Z* peak, while it is very small in the *Z*-peak region $m_{ee}=66$–$116\,\,\text {GeV}$. The background from electroweak and top-quark processes ranges from 6.2 to 8.8% for $m_{ee}=46$–$66\,\,\text {GeV}$, 0.23–0.46% for $m_{ee}=66$–$116\,\,\text {GeV}$ and 2.0–8.5% for $m_{ee}=116$–$150\,\,\text {GeV}$, where a larger background contamination is typically found at central rapidity.

To separate the central $Z/\gamma ^* \rightarrow e^+e^-$ signal from the multijet background, the analysis relies on the same $E_\mathrm {T}^\mathrm {cone30}$ quantity as described for the $W \rightarrow e \nu$ case. The minimum of the value $E_\mathrm {T}^\mathrm {cone30}/p_{\text {T}}$ of the two electron candidates is chosen to represent each event, as it was found to provide optimal discrimination. The multijet fraction is then estimated from data by fitting this distribution using a template method similar to the $W \rightarrow e \nu$ analysis. The background template is selected with inverted electron identification requirements and the signal $Z/\gamma ^* \rightarrow e^+e^-$, electroweak and $t\bar{t}$ templates are taken from simulation. The non-isolated sample where the minimum of $E_\mathrm {T}^\mathrm {cone30}/p_{\text {T}}$ of both electrons exceeds a certain value is found to be dominated by multijet background and is used to adjust the normalization of the background template, taking into account the small signal contamination. The right panel of Fig. 1 shows the isolation distribution used to obtain the multijet background in the *Z*-peak region. This procedure yields a fraction of multijet background decreasing towards larger rapidity with a typical size between 1.9 and 5.0% in the low dielectron mass bin, between 0.14 and 1.6% at high dielectron mass and between 0.02 and 0.15% near the *Z* peak. Uncertainties are dominated by the statistical uncertainty of the sample containing non-isolated electron candidates and by the sensitivity of the procedure to the threshold applied to the minimum of $E_\mathrm {T}^\mathrm {cone30}/p_{\text {T}}$ to select the non-isolated region and amount to typically 20% at and above the *Z* peak ($66<m_{\ell \ell }< 150\,\,\text {GeV}$) and 10% below ($46<m_{\ell \ell }< 66\,\,\text {GeV}$).

In the forward $Z/\gamma ^* \rightarrow e^+e^-$ analysis, the multijet background is estimated with the same technique as described for the central $Z \rightarrow e^+e^-$ analysis, although only the isolation distribution of the central electron is used. In total the multijet background is estimated to be 1.4–2.4% in the *Z*-peak region and 18–26% in the high-mass region. The total relative uncertainties in these estimates are at the level of 10%.

Furthermore, there is a significant contamination from $W(\rightarrow e\nu )+$jets events in the forward $Z/\gamma ^* \rightarrow e^+e^-$ channel, where the electron from the *W* decay is detected in the central region and an associated jet mimics the signature of an electron in the forward region. As the associated jet production and fake-electron rates may be poorly modelled by the simulation, the $W \rightarrow e \nu$ background component is determined by a data-driven procedure. A control region is constructed starting from the nominal forward $Z/\gamma ^* \rightarrow e^+e^-$ event selection, but removing the *Z*-peak region $m_{ee}= 80$–$100\,\text {GeV}$ and requiring $E_{\text {T}}^{\text {miss}}$ and $m_\mathrm {T}$ selections similar to the $W \rightarrow e \nu$ signal analysis. It is found that the Powheg+Pythia6 $W \rightarrow e \nu$ samples describe well all relevant kinematic variables such as the invariant mass $m_{ee}$ or dielectron rapidity $y_{ee}$ in the control region after applying an additional normalization factor of $1.6 \pm 0.2$. This factor is then also applied to the Powheg+Pythia6 $W \rightarrow e \nu$ samples in the forward $Z/\gamma ^* \rightarrow e^+e^-$ signal region. The assigned uncertainty of this scale factor covers systematic uncertainties induced by the extrapolation and is estimated using variations of the control region with different $E_{\text {T}}^{\text {miss}}$ or $m_\mathrm {T}$ selections. Other, smaller electroweak contributions from $t\bar{t}$ and diboson production are estimated using the corresponding MC samples. The total $W \rightarrow e \nu$ and other electroweak backgrounds to the forward $Z/\gamma ^* \rightarrow e^+e^-$ channel is about 1.9% at the *Z* peak and up to 22% in the high-mass region. While the multijet background fraction is found to be essentially independent of the dielectron rapidity $y_{ee}$, the $W \rightarrow e \nu$ and other electroweak backgrounds decrease towards larger $y_{ee}$.

## Muon channel measurements

### Event selection

The same requirement for a primary vertex is imposed as for the electron channels. The analysis uses muon candidates that are defined as “combined muons” in Ref. [81]. For combined muons an independent track reconstruction is performed in the ID and the MS, and a combined track is formed using a $\chi ^2$ minimization procedure. In order to reject cosmic-ray background, the *z* position of the muon track extrapolated to the beam line has to match the *z* coordinate of the primary vertex within $\pm 1$ cm. The ID track is required to satisfy the track-hit requirements described in Ref. [81]; in addition, the ID track must include a position measurement from the innermost layer of the pixel detector. To reduce background from non-isolated muons produced in the decay of hadrons within jets, muons are required to be isolated. This is achieved with a track-based isolation variable defined as the sum of transverse momenta of ID tracks with $p_{\text {T}} > 1\,\text {GeV}$ within a cone $\Delta R = 0.4$ around the muon direction and excluding the muon track, denoted as $p_\mathrm {T}^\mathrm {cone40}$. The value of $p_\mathrm {T}^\mathrm {cone40}$ is required to be less than 10% of the muon $p_{\text {T}}$. The efficiency of this isolation requirement is about 92% for signal muons with $p_{\text {T}} =20\,\,\text {GeV}$ and increases to about 99% for $p_{\text {T}} >40\,\,\text {GeV}$.

Events in the muon channels were selected during data-taking with a trigger demanding the presence of a single muon with $p_{\text {T}} > 18\,\,\text {GeV}$. The selection of *W* events demands one muon with $p_{\text {T}} > 25\,\text {GeV}$ and $|\eta | < 2.4$, while a veto on any further muon with $p_{\text {T}} >20\,\,\text {GeV}$ is imposed to reduce contamination from the $Z/\gamma ^* \rightarrow \mu ^+\mu ^-$ process. The same missing transverse momentum $E_{\text {T}}^{\text {miss}} >25\,\,\text {GeV}$ and transverse mass $m_\mathrm {T}>40\,\,\text {GeV}$ requirements are imposed as in the $W \rightarrow e \nu$ analysis. Events for the $Z/\gamma ^* \rightarrow \mu ^+\mu ^-$ analysis are selected by requiring exactly two muons with $p_{\text {T}} > 20\,\text {GeV}$ and $|\eta | < 2.4$. The two muons are required to be of opposite charge, and the invariant mass of the $\mu ^+\mu ^-$ pair, $m_{\mu \mu }$, is required to be between 46 and 150 GeV.

### Calibration and efficiencies

Muon transverse momentum corrections and trigger and reconstruction efficiencies are measured using the same methods as applied in Ref. [1] and documented in Refs.  [81, 82]. Muon transverse momentum resolution corrections are determined comparing data and MC events as a function of $\eta$ in the barrel and end-cap regions [81]. They are derived by fitting the $Z \rightarrow \mu ^+\mu ^-$ invariant mass spectrum and the distributions of $1/p_{\text {T}} ^\mathrm {ID} - 1/p_{\text {T}} ^\mathrm {MS}$ for both $\mu ^+$ and $\mu ^-$, where $p_{\text {T}} ^\mathrm {ID}$ and $p_{\text {T}} ^\mathrm {MS}$ are the muon transverse momenta in $Z \rightarrow \mu ^+\mu ^-$ and $W \rightarrow \mu \nu$ events, measured in only the ID and the muon spectrometer, respectively. Muon transverse momentum scale corrections are measured by comparing the peak positions in the data and MC $Z \rightarrow \mu ^+\mu ^-$ invariant mass distributions. Further charge-dependent corrections are derived by comparing the muon transverse momentum distributions in $Z \rightarrow \mu ^+\mu ^-$ events for positive and negative muons [81, 83]. The momentum scale in the simulation is found to be higher than in the data by about 0.1–0.2% in the central region and 0.3–0.4% in the forward region. An additional, momentum-dependent correction is applied to account for charge-dependent biases. For a transverse momentum of $40\,\text {GeV}$ this correction is less than 0.1% in the central region and extends to 0.5% in the forward region. The muon momentum resolution is found to be 2–5% worse in the data than in the simulation. All scale and resolution corrections are applied to the simulated event samples to match the characteristics of the data.

Muon trigger and reconstruction efficiencies are measured with a tag-and-probe method in a sample of $Z \rightarrow \mu ^+\mu ^-$ events. Imposing tighter selections on the invariant mass and on the angular correlation between the two muons reduces the background contamination and allows one of the muons to be selected with looser requirements to measure the efficiencies [81]. The reconstruction efficiencies are measured using a factorized approach: the efficiency of the combined reconstruction is derived with respect to the ID tracks, and the efficiency of reconstructing a muon in the inner tracker is measured relative to the MS tracks. The isolation selection efficiency is estimated relative to combined tracks. Finally, the trigger efficiency is measured relative to isolated combined muons.

The measured data-to-simulation ratios of efficiencies are applied as corrections to the simulation. In general, these factors are close to unity, indicating that the simulation reproduces detector effects very well. The corrections for the combined reconstruction efficiency are 1–2%, except for a small region around $|\eta | \simeq 1.0$ where a larger correction of 6–7% is applied to account for muon chambers simulated but not installed. These correction factors are parameterized in $\eta$ and $\phi$ and they are determined with a 0.1–0.3% relative uncertainty. The efficiency of the isolation requirement is also modelled well in the simulation. The correction is derived as a function of the transverse momentum and is about 1% for $p_{\text {T}} = 20\,\text {GeV}$ and decreases as $p_{\text {T}}$ increases to reach about 0.2% for $p_{\text {T}} > 40\,\,\text {GeV}$. The relative uncertainty of the isolation efficiency correction is about 0.1–0.3%. A larger correction is needed to account for the mismodelling of the trigger efficiency in simulation, ranging from 5–10%. This is parameterized as a function of $\eta$ and $p_{\text {T}}$ and known with a 0.1–0.8% relative uncertainty.

### Backgrounds

The electroweak background in the $W \rightarrow \mu \nu$ channel is dominated by $W \rightarrow \tau \nu$ and $Z/\gamma ^* \rightarrow \mu ^+\mu ^-$ events and is estimated with the simulation. Relative to the number of all $W^\pm$ candidate events, the $W \rightarrow \tau \nu$ contribution is determined to be between 1.9 and 2.1% for the different bins of pseudorapidity and is a similar fraction of $W^+$ and $W^-$ events. The $Z/\gamma ^* \rightarrow \mu ^+\mu ^-$ contribution is estimated to be between 1.1 and 5.7%. Further contributions at the 0.1–0.8% level arise from $t\bar{t}$, $Z/\gamma ^* \rightarrow \tau ^+\tau ^-$, single top-quark and diboson production. The sum of electroweak and top-quark backgrounds ranges from 4.5 to 9.6% in the $W^-$ channel and from 4.0 to 7.0% in the $W^+$ channel. In contrast to $W \rightarrow \tau \nu$ background, the other electroweak and top-quark background yields are of similar absolute size in $W^+$ and $W^-$ events.

**Fig. 2:**
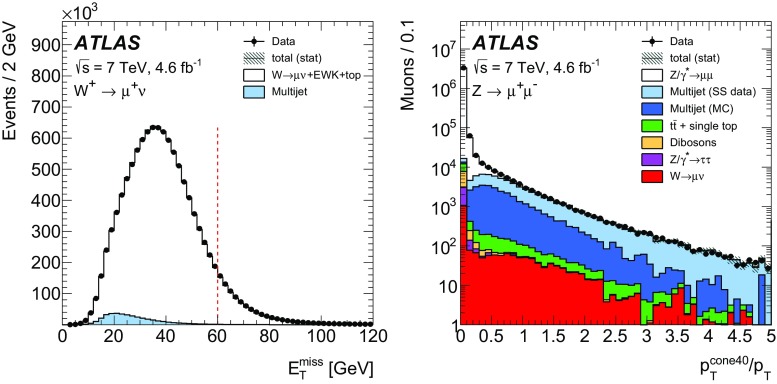
Distributions used for the estimation of the multijet background in the $W \rightarrow \mu \nu$ channel (*left*) and $Z/\gamma ^* \rightarrow \mu ^+\mu ^-$ channel (*right*). For the $W \rightarrow \mu \nu$ channel, the result of the template fit using the $E_{\text {T}}^{\text {miss}}$ distribution is shown. The *vertical line* indicates the upper boundary ($E_{\text {T}}^{\text {miss}} = 60\,\,\text {GeV}$) of the region used in the fit. The label “EWK+top” refers to the electroweak and top-quark background contributions estimated from MC simulation, which are here treated in a common template together with the $W \rightarrow \mu \nu$ signal. In the $Z/\gamma ^* \rightarrow \mu ^+\mu ^-$ channel, the full $p_\mathrm {T}^\mathrm {cone40}/p_{\text {T}}$ distribution is used to normalize the multijet template from data. The sum of all expected background and signal contributions is shown as a *solid line* with a *hashed band* detailing the statistical uncertainty and labelled “total (stat)”

The multijet background in the $W \rightarrow \mu \nu$ channel originates primarily from heavy-quark decays, with smaller contributions from pion and kaon decays in flight and fake muons from hadrons that punch through the calorimeter. Given the uncertainty in the dijet cross-section prediction and the difficulty of properly simulating non-prompt muons, the multijet background is derived from data. The number of background events is determined from a binned maximum-likelihood template fit to the $E_{\text {T}}^{\text {miss}}$ distribution, as shown in the left panel of Fig. 2. The fit is used to determine the normalization of two components, one for the signal and electroweak plus top-quark backgrounds, taken from simulation, and a second for the multijet background, derived from data. No prior knowledge of the normalization of the two components is assumed. The multijet template is derived from a control sample defined by reversing the isolation requirement imposed to select the signal and without applying any requirement on $E_{\text {T}}^{\text {miss}}$. The fits are done separately for $W^+$ and $W^-$ events and in each $\eta$ region of the differential cross-section measurement.

This analysis yields a fraction of multijet background events between 2.7% in the most central pseudorapidity bin and 1.3% in the most forward bin of the measurement for the $W^+$ channel and between 3.5 and 2.6% for the $W^-$ channel, respectively. The systematic uncertainty, dominated by the uncertainty in the $E_{\text {T}}^{\text {miss}}$ modelling for signal events in simulation, is estimated to be about 0.4–0.8% relative to the number of background events. While this background is determined separately for $W^+$ and $W^-$ samples, the resulting background yields are found to be compatible between both charges within the statistical uncertainty. As in the electron channel, the multijet background was also determined with an alternative method following Ref. [7], which gives an estimate well within the systematic uncertainty assigned to the baseline determination described above.

The background contributions in the $Z/\gamma ^* \rightarrow \mu ^+\mu ^-$ channel due to isolated muons from $t\bar{t}$, $Z/\gamma ^* \rightarrow \tau ^+\tau ^-$, and diboson production behave similarly to those in the electron channel. In the *Z*-peak region, $m_{\mu \mu }=66$–$116\,\,\text {GeV}$, these are estimated to be 0.1, 0.07, and 0.1%, respectively. The total background from electroweak and top-quark processes outside the *Z*-peak region is around 6% for $m_{\mu \mu }=46$–$66\,\,\text {GeV}$ and around 4% for $m_{\mu \mu }=116$–$150\,\,\text {GeV}$.

The multijet background in the $Z/\gamma ^* \rightarrow \mu ^+\mu ^-$ channel is estimated from data using various methods. The first class of methods is based on binned maximum-likelihood template fits using different discriminating distributions: the isolation, transverse impact parameter and $p_{\text {T}}$ of the muon, and the dimuon invariant mass. The templates for the multijet background are derived in most cases from data control samples obtained by inverting the requirements on muon isolation or the opposite-charge requirement on the muon pair, depending on the quantity fitted. Alternative templates are also derived from simulation of inclusive heavy-flavour production with semileptonic decays of charm or bottom hadrons to muons. The right panel of Fig. 2 shows the result of the template fit in the muon isolation distribution to determine the absolute scale of the multijet background, which is then extrapolated to the isolated region. For this particular method, the multijet template is modelled by a combination of same-charge data events, used to represent the background from light-quark production, and a contribution from simulated heavy-flavour production, where the small same-charge fraction is subtracted from the dominant opposite-charge dimuon contribution.

In addition to the template fits, a method extrapolating from control regions defined by inverting the isolation, opposite charge, or both requirements is employed. All methods, apart from the template fit in $m_{\mu \mu }$, are performed separately in the three mass regions of the differential $Z/\gamma ^* \rightarrow \mu ^+\mu ^-$ cross-section measurements. The fraction of background events is calculated as the weighted average of these measurements and found to be $0.09\%$ in the $m_{\mu \mu }=66$–$116\,\text {GeV}$ mass region. The relative statistical uncertainty is $50\%$. A relative systematic uncertainty of $80\%$ is assigned based on the spread of the weighted measurements. In the $m_{\mu \mu }= 46$–66 (116–150) $\,\text {GeV}$ mass region the fraction of multijet background events is estimated to be 0.5 (0.2)% with relative statistical and systematic uncertainties of $15\%$ ($14\%$) and $80\%$ ($60\%$), respectively.

The shape of the multijet background as a function of $y_{\mu \mu }$ is derived from a simulated sample of multijet events selected with a looser muon isolation requirement to increase the statistical precision. Systematic uncertainties in the shape of the multijet background as a function of $y_{\mu \mu }$ are assessed by comparing the shape in simulation obtained with the looser and nominal muon selections as well as comparing the shape predicted by the simulation to the shape in a data control region, where at least one muon fails either the isolation or transverse impact parameter requirements. An additional relative uncertainty of 22% is obtained, treated as uncorrelated in rapidity and mass bins.

Cosmic-ray muons overlapping in time with a collision event are another potential source of background. From a study of non-colliding bunches, this background contribution is found to be negligible.

## Cross-section results

### Analysis procedure

The integrated and differential $W^+ \rightarrow \ell ^+\nu$, $W^- \rightarrow \ell ^-\bar{\nu }$, and $Z/\gamma ^* \rightarrow \ell \ell$ production cross sections times the branching ratio for decays into a single lepton flavour ($\ell =e$ or $\mu$) are measured in fiducial volumes as defined in Sect. 2.3. Integrated fiducial cross sections in the electron (muon) channel are computed following the equation

5 $$\begin{aligned} \sigma ^\mathrm {fid,e(\mu )}_{W \rightarrow e(\mu )\nu [Z\rightarrow ee(\mu \mu )]} = \frac{N_{W[Z]} - B_{W[Z]}}{{C_{W[Z]}}\cdot L_\mathrm {int}} , \end{aligned}$$

where $N_{W[Z]}$ is the number of observed signal candidates in data and $B_{W[Z]}$ is the number of background events expected in the selected sample. The integrated luminosity of the sample is $L_\mathrm {int}=(4.58\pm 0.08)\,\mathrm {fb}^{-1}$ for all channels except the $W \rightarrow e \nu$ analysis, where it is $L_\mathrm {int}=(4.51\pm 0.08)\,\mathrm {fb}^{-1}$. A correction for the event detection efficiency is applied with the factor $C_{W[Z]}$ , which is obtained from the simulation as

6 $$\begin{aligned} {C_{W[Z]}}= \frac{N^\mathrm {MC, rec}_{W[Z]}}{N^\mathrm {MC, gen, fid}_{W[Z]}}. \end{aligned}$$

Here, $N^\mathrm {MC, rec}_{W[Z]}$ is the sum of event weights after simulation, reconstruction and selection, adjusted for the observed data-to-simulation differences such as in reconstruction, identification, and trigger efficiencies. The denominator $N^\mathrm {MC, gen, fid}_{W[Z]}$ is computed with generator-level information after fiducial requirements. To correct the measurements for QED FSR effects, the fiducial requirements at generator level are implemented using the lepton momenta before photon radiation. The lepton pairs ($\ell ^+\ell ^-$, $\ell ^+\nu$ or $\ell ^-\bar{\nu }$) are required to originate directly from the decay of the $Z/\gamma ^*$ or $W^\pm$ bosons. The $C_{W[Z]}$ correction is affected mostly by experimental uncertainties, which are described in Sects. 3 and 4.

The following uncertainties in $C_{W[Z]}$ of theoretical origin are considered. PDF-induced uncertainties are determined by reweighting the signal samples [84] to the 26 eigenvectors of the CT10 set and scaling the resulting uncertainty to 68% confidence level (CL). The effect of an imperfect description of the boson transverse momentum spectra is estimated by an additional reweighting of the $W^\pm$ and $Z/\gamma ^*$ samples, beyond that discussed in Sect. 2.2, by the data-to-simulation ratio observed in the *Z*-peak region. Uncertainties related to the implementation of the NLO QCD matrix element and its matching to the parton shower are estimated from the difference between the $C_{W[Z]}$ correction factors obtained from the Powheg+Herwig and MC@NLO+Herwig signal samples. A similar systematic uncertainty related to the signal modelling is estimated by changing the parton showering, hadronization, and underlying event by comparing analysis results using Powheg+Pythia6 and Powheg+Herwig samples. When changing the signal generator, the $C_{W[Z]}$ correction factors vary by small amounts due to differences in the simulated charged-lepton and neutrino kinematics, the detector response to the hadronic recoil, and the electron and muon identification and isolation efficiencies. The full data-driven estimate of multijet background in the $W \rightarrow \ell \nu$ channels is repeated when changing the signal samples, as the reconstructed $E_{\text {T}}^{\text {miss}}$ and $m_\mathrm {T}$ shapes have a significant impact in the fit.

For the measurement of charge-separated $W^+$ and $W^-$ cross sections, the $C_W$ factor is modified to incorporate a correction for event migration between the $W^+$ and $W^-$ samples as

7 $$\begin{aligned} C_{W^+} = \frac{N^\mathrm {MC, rec+}_{W}}{N^\mathrm {MC, gen+, fid}_{W}} \,\,\,\,\,\,\text{ and }\,\,\,\,\,\, C_{W^-} = \frac{N^\mathrm {MC, rec-}_{W}}{N^\mathrm {MC, gen-, fid}_{W}}, \end{aligned}$$

where $N^\mathrm {MC, rec+}_{W}$ and $N^\mathrm {MC, rec-}_{W}$ are sums of event weights reconstructed as $W^+$ or $W^-$, respectively, regardless of the generated charge; similarly $N^\mathrm {MC, gen+, fid}_{W}$ and $N^\mathrm {MC, gen-, fid}_{W}$ are sums of events generated as $W^+$ and $W^-$, respectively, regardless of the reconstructed lepton charge. This charge misidentification effect is only relevant for the electron channels and negligible in the muon channels.

The correction of the differential distributions follows a similar methodology, but it is performed using the Bayesian Iterative method [85, 86], as implemented in the RooUnfold package [87] using three iterations. The differential distributions considered in this paper are constructed to have bin purities of typically more than 90%, where the bin purity is defined as the ratio of events generated and reconstructed in a certain bin to all events reconstructed in that bin. Slightly lower purities of 80–90% are observed in the $Z/\gamma ^*$ analyses below the *Z*-peak region ($m_{\ell \ell }=46$–$66\,\,\text {GeV}$) due to QED FSR effects and in the forward $Z \rightarrow e^+e^-$ analysis due to worse experimental resolution. Because of the very high bin purities, the unfolding is to a large extent reduced to an efficiency correction. Residual prior uncertainties are covered by the variations of theoretical origin as discussed for the $C_{W[Z]}$ factors above.

Fiducial cross sections in the electron and muon channels, as reported in Sects. 5.2.1 and 5.2.2, are then extrapolated to the common fiducial volume by applying a small correction $E^{e(\mu )}_{W[Z]}$ as mentioned in Sect. 2.3:

8 $$\begin{aligned} \sigma ^\mathrm {fid}_{W \rightarrow \ell \nu [Z\rightarrow \ell \ell ]} = \frac{\sigma ^\mathrm {fid, e(\mu )}_{W \rightarrow e(\mu )\nu [Z\rightarrow ee(\mu \mu )]}}{E^{e(\mu )}_{W[Z]}}. \end{aligned}$$

These $E^{e(\mu )}_{W[Z]}$ corrections account for the different $\eta$ acceptances for electrons and muons in both the CC and NC analyses and are calculated from the nominal signal samples generated with Powheg+Pythia6. These correction factors are typically in the range of 0.90–0.95, but are as low as 0.65 in a few bins at high lepton pseudorapidity or dilepton rapidity. Uncertainties in these extrapolation factors account for PDF uncertainties as well as further signal modelling uncertainties obtained by comparing samples generated with Powheg+Herwig and MC@NLO. These uncertainties are found to be small, ${\sim } 0.1\%$, and are always well below the experimental precision of the measurements.

The total $W^{\pm } \rightarrow \ell \nu$ and $Z/\gamma ^* \rightarrow \ell \ell$ cross sections, times leptonic branching ratio, are calculated using the relation

9 $$\begin{aligned} \sigma ^\mathrm {tot}_{W \rightarrow \ell \nu [Z \rightarrow \ell \ell ]} = \frac{\sigma ^\mathrm {fid}_{W \rightarrow \ell \nu [Z \rightarrow \ell \ell ]}}{{A_{W[Z]}}}, \end{aligned}$$

where the acceptance $A_{W[Z]}$ extrapolates the cross section for the $W^+$, $W^-$ and the $Z/\gamma ^*$ channels, measured in the fiducial volume, $\sigma ^\mathrm {fid}_{W \rightarrow \ell \nu [Z \rightarrow \ell \ell ]}$, to the full kinematic region. It is given by

10 $$\begin{aligned} {A_{W[Z]}}= \frac{N^\mathrm {MC, gen, fid}_{W[Z]}}{N^\mathrm {MC, gen, tot}_{W[Z]}}, \end{aligned}$$

where $N^\mathrm {MC, gen, tot}_{W[Z]}$ is the total sum of weights of all generated MC events. Uncertainties in the acceptance from the theoretical uncertainties in the process modelling and in the PDFs are estimated as indicated above and amount to typically ±(1.5–2.0)%. This therefore significantly increases the uncertainty in the total cross sections with respect to the fiducial cross sections.

### Cross-section measurements

#### Electron channels

**Fig. 3:**
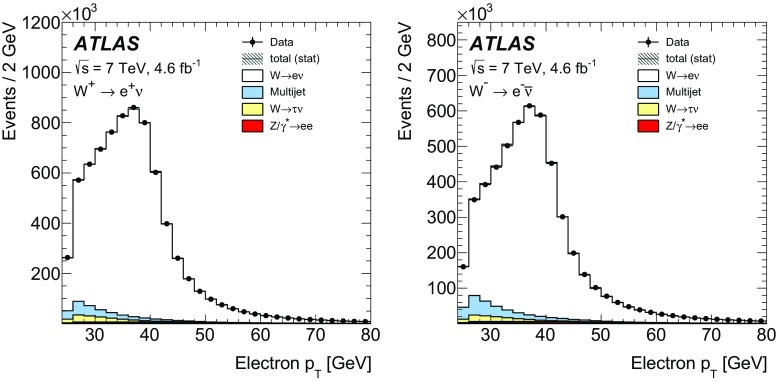
The transverse momentum distribution of electrons for $W^+ \rightarrow e^+ \nu$ candidates (*left*) and $W^- \rightarrow e^- \bar{\nu }$ candidates (*right*). The simulated samples are normalized to the data luminosity. The multijet background shape is taken from a data control sample and normalized to the estimated yield of multijet events. The sum of all expected background and signal contributions is shown as a* solid line with a hashed band* detailing the statistical uncertainty and labelled “total (stat)”. The legend lists only background sources with a visible contribution

**Fig. 4:**
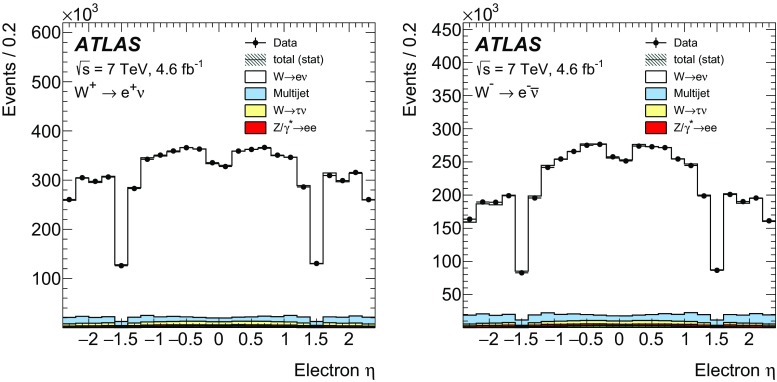
The pseudorapidity distribution of electrons for $W^+ \rightarrow e^+ \nu$ candidates (*left*) and $W^- \rightarrow e^- \bar{\nu }$ candidates (*right*). The simulated samples are normalized to the data luminosity. The multijet background shape is taken from a data control sample and normalized to the estimated yield of multijet events. The sum of all expected background and signal contributions is shown as a* solid line with a hashed band* detailing the statistical uncertainty and labelled “total (stat)”. The legend lists only background sources with a visible contribution

**Fig. 5:**
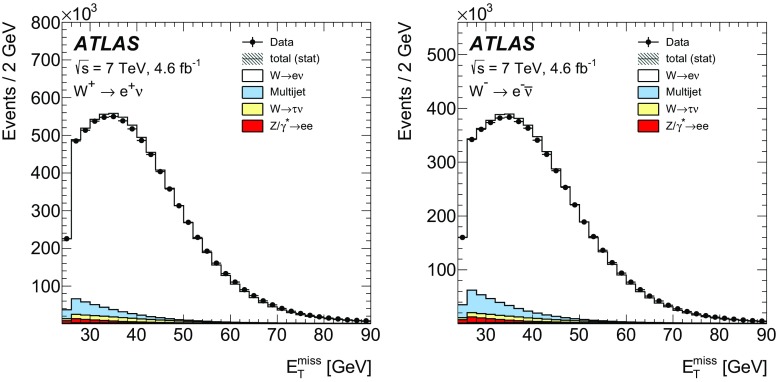
The missing transverse momentum distribution for $W^+ \rightarrow e^+ \nu$ candidates (*left*) and $W^- \rightarrow e^- \bar{\nu }$ candidates (*right*). The simulated samples are normalized to the data luminosity. The multijet background shape is taken from a data control sample and normalized to the estimated yield of multijet events. The sum of all expected background and signal contributions is shown as a* solid line with a hashed band* detailing the statistical uncertainty and labelled “total (stat)”. The legend lists only background sources with a visible contribution

**Fig. 6:**
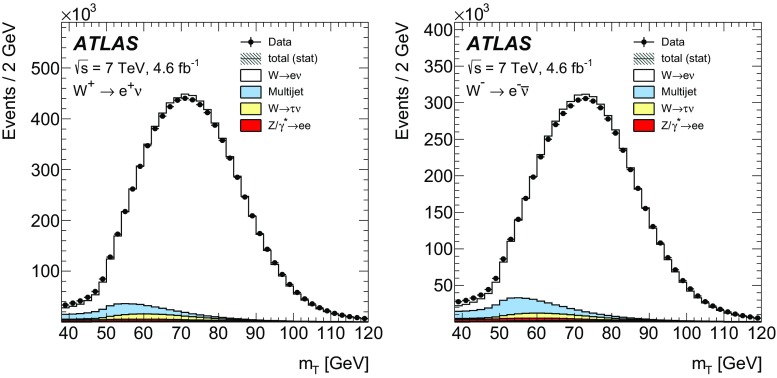
The transverse mass distribution for $W^+ \rightarrow e^+ \nu$ candidates (*left*) and $W^- \rightarrow e^- \bar{\nu }$ candidates (*right*). The simulated samples are normalized to the data luminosity. The multijet background shape is taken from a data control sample and normalized to the estimated yield of multijet events. The sum of all expected background and signal contributions is shown as a* solid line with a hashed band* detailing the statistical uncertainty and labelled “total (stat)”. The legend lists only background sources with a visible contribution

**Fig. 7:**
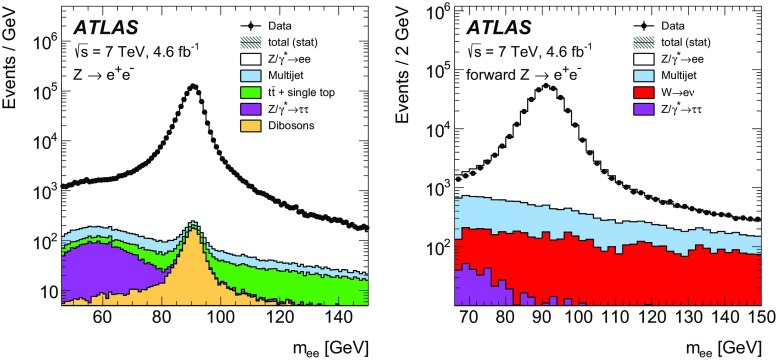
The dilepton invariant mass distributions for $Z/\gamma ^* \rightarrow e^+e^-$ candidates with two central electrons (*left*) and one central and one forward electron (*right*). The simulated samples are normalized to the data luminosity. The multijet background shape is taken from a data control sample and normalized to the estimated yield of multijet events. The sum of all expected background and signal contributions is shown as a* solid line with a hashed band* detailing the statistical uncertainty and labelled “total (stat)”. The legend lists only background sources with a visible contribution

**Fig. 8:**
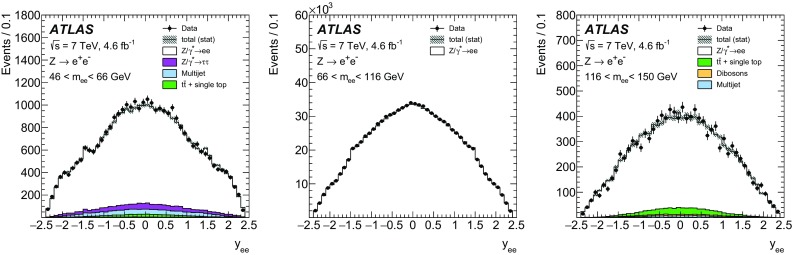
The dilepton rapidity distributions for $Z/\gamma ^* \rightarrow e^+e^-$ candidates with two central electrons in the mass regions $46< m_{ee}< 66\,\text {GeV}$ (*left*), $66< m_{ee}< 116\,\text {GeV}$ (*middle*) and $116< m_{ee}< 150\,\text {GeV}$ (*right*). The simulated samples are normalized to the data luminosity. The multijet background shape is taken from a data control sample and normalized to the estimated yield of multijet events. The sum of all expected background and signal contributions is shown as a* solid line with a hashed band* detailing the statistical uncertainty and labelled “total (stat)”. The legend lists only background sources with a visible contribution

**Fig. 9:**
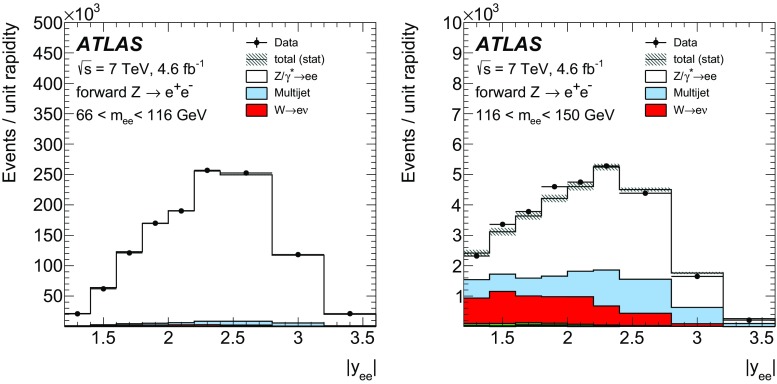
The dilepton rapidity distributions for $Z/\gamma ^* \rightarrow e^+e^-$ candidates with one central and one forward electron in the mass region $66< m_{ee}< 116\,\text {GeV}$ (*left*) and $116\,\text {GeV}< m_{ee}< 150\,\text {GeV}$ (*right*). The simulated samples are normalized to the data luminosity. The multijet background shape is taken from a data control sample and normalized to the estimated yield of multijet events. The sum of all expected background and signal contributions is shown as a* solid line with a hashed band* detailing the statistical uncertainty and labelled “total (stat)”. The legend lists only background sources with a visible contribution

To ensure an adequate description of important kinematic variables in the electron channels, Figs. 3, 4, 5, 6, 7, 8 and 9 compare several distributions of the data to the signal simulation and estimated backgrounds. The signal and electroweak background distributions are taken from the simulation and normalized to the corresponding data luminosity. The distributions of the background from multijet production are obtained from data and normalized as described in Sect. 3.3. Figures 3, 4, 5 and 6 show the distributions of the electron transverse momentum, the electron pseudorapidity, the missing transverse momentum, and the transverse mass of candidate *W* events, respectively. The invariant mass distribution of electron pairs, selected by the $Z/\gamma ^* \rightarrow e^+e^-$ analyses, and the dilepton rapidity distributions are shown in Figs. 7, 8 and 9, respectively. Good agreement between data and the predictions is observed in general for all kinematic distributions. Small disagreements in the shapes of the $E_{\text {T}}^{\text {miss}}$ and $m_\mathrm {T}$ distributions of *W*-boson candidates are visible at the level of 2–10%. These deviations are covered by uncertainties on the multijet background and on the signal modelling, for the latter specifically the variations related to the hadronic recoil response and W-boson $p_{\text {T}}$ spectrum. In the forward $Z/\gamma ^* \rightarrow e^+e^-$ distributions, small disagreements at low $m_{ee}$ and localised in particular $y_{ee}$ bins of the high mass region $m_{ee}=116$–$150\,\text {GeV}$ are covered by the systematic uncertainties on the electron energy scale and resolution, and background yields, respectively.

Table 1 summarizes the number of selected candidates, estimated background events and the $C_{W[Z]}$ correction factors used for the four different integrated electron channel measurements: $W^+$, $W^-$, central $Z/\gamma ^*$, and forward $Z/\gamma ^*$ analyses, both $Z/\gamma ^*$ analyses in the *Z*-peak region of $66<m_{ee}<116\,\,\text {GeV}$. The corresponding four integrated cross sections in the fiducial phase space specific to the electron channels are reported in Table 2 with their uncertainties due to data statistics, luminosity, and further experimental systematic uncertainties.

The systematic uncertainties split into their different components are shown in Table 3. Apart from the luminosity contribution of $1.8$%, the $W \rightarrow e \nu$ cross section is measured with an experimental uncertainty of 0.9% for the $W^+$ channel and 1.1% for the $W^-$ channel. The central $Z/\gamma ^* \rightarrow e^+e^-$ cross section in the *Z*-peak region is measured with an uncertainty of 0.35%. The extended forward rapidity $Z/\gamma ^* \rightarrow e^+e^-$ cross section is measured with an uncertainty of 2.3%.

The uncertainties of the data-driven determinations of the electron and hadronic recoil responses, discussed in Sect. 3.2, are propagated to the measurements. These comprise uncertainties in the electron detection efficiencies, separated into contributions from the trigger, reconstruction, identification, and isolation, which are relatively small for the $W \rightarrow e \nu$ channel, about 0.2% in total, but constitute the dominant systematic uncertainties in the central *Z* data and amount to 0.25%. In the forward *Z* analysis the dominant systematic uncertainty, of about 1.5%, comes from the forward electron identification. The effects from charge misidentification only affect the $W^{\pm } \rightarrow e \nu$ cross sections and are very small, ${<}0.1\%$. Both the central and forward electron $p_{\text {T}}$ resolution and scale uncertainties are in general subdominant, amounting to about 0.2%. The $W \rightarrow e \nu$ analyses are also affected by uncertainties in the hadronic recoil response, decomposed into soft $E_{\text {T}}^{\text {miss}}$ and jet energy scale and resolution uncertainties, which add up to a total contribution of about 0.2%.

Signal modelling variations using different event generators, as discussed in Sect. 5.1, contribute significant uncertainties of 0.6–0.7% to the $W \rightarrow e \nu$ analysis and 1.1% to the forward *Z* analysis, while the effect on the central *Z* analysis is smaller with 0.2%. This source of uncertainty comprises effects from the lepton efficiencies and, for the $W \rightarrow e \nu$ analysis, effects from the multijet background determination, which relies on $E_{\text {T}}^{\text {miss}}$ and $m_\mathrm {T}$ shapes, and the hadronic recoil response. Other theoretical modelling uncertainties, due to PDFs and boson $p_{\text {T}}$ effects, are at the level of 0.1–0.2%.

Uncertainties in the background subtraction are discussed in Sect. 3.3. The contribution from the electroweak and top-quark backgrounds is small and ${<}0.2\%$ for all channels. The multijet background to the $W \rightarrow e \nu$ channel, however, represents one of the dominant uncertainties with 0.6–0.7%.

**Table 1:** Number of observed event candidates *N*, of estimated background events *B*, and the correction factors *C* for the $W^+$, $W^-$, central and forward $Z/\gamma ^*$ ($66< m_{ee}<116\,\text {GeV}$) electron channels. The correction factors *C* were defined in Eq. (6). The charge asymmetry in the background to the $W^{\pm }$ channels stems from the $W \rightarrow \tau \nu$ contribution, which is proportional to the signal yield. The given uncertainties are the sum in quadrature of statistical and systematic components. The statistical uncertainties in *C* are negligible

	*N*	*B*	*C*
$W^+ \rightarrow e^+ \nu$	7,552,884	$515{,}000\pm 48{,}000$	$0.572\pm 0.004$
$W^- \rightarrow e^- \bar{\nu }$	5,286,997	$468{,}000\pm 40{,}000$	$0.586\pm 0.005$
Central $Z/\gamma ^* \rightarrow e^+e^-$	1,011,940	$4750\pm 350$	$0.500\pm 0.002$
Forward $Z/\gamma ^* \rightarrow e^+e^-$	321,575	$9170\pm 460$	$0.425\pm 0.010$

**Table 2:** Fiducial cross sections times branching ratios for $W^+$, $W^-$, central and forward $Z/\gamma ^*$ ($66<m_{ee}<116\,\text {GeV}$) production in the electron decay channels. The fiducial regions used for the measurement are those defined for the combined fiducial regions in Sect. 2.3, except that the central electron pseudorapidity is restricted to be $|\eta |<2.47$ and excludes $1.37<|\eta |<1.52$, and the forward electron pseudorapidity excludes the region $3.16<|\eta |<3.35$. The uncertainties denote the statistical (stat), the systematic (syst) and the luminosity (lumi) uncertainties

	$$\sigma ^\mathrm {fid,e}_{W \rightarrow e\nu }$$ (pb)
$W^+ \rightarrow e^+ \nu$	$~2726 \pm 1 \,\mathrm {(stat)} \pm 28 \,\mathrm {(syst)} \pm 49\,\mathrm {(lumi)}$
$W^- \rightarrow e^- \bar{\nu }$	$~1823 \pm 1 \,\mathrm {(stat)} \pm 21 \,\mathrm {(syst)} \pm 33\,\mathrm {(lumi)}$

	$$\sigma ^\mathrm {fid,e}_{Z/\gamma ^* \rightarrow ee}$$ (pb)
Central $Z/\gamma ^* \rightarrow e^+e^-$	$439.5 \pm 0.4 \,\mathrm {(stat)} \pm 1.5 \,\mathrm {(syst)} \pm 7.9 \,\mathrm {(lumi)}$
Forward $Z/\gamma ^* \rightarrow e^+e^-$	$160.2 \pm 0.3 \,\mathrm {(stat)} \pm 3.7 \,\mathrm {(syst)} \pm 2.9 \,\mathrm {(lumi)}$

**Table 3:** Relative uncertainties $\delta \sigma$ in the measured integrated fiducial cross sections times branching ratios of $W^+$, $W^-$, central and forward $Z/\gamma ^*$ ($66< m_{ee}<116\,\text {GeV}$) in the electron channels

	$\delta \sigma _{W+}$ (%)	$\delta \sigma _{W-}$ (%)	$\delta \sigma _{Z}$ (%)	$\delta \sigma _{\mathrm {forward}\,Z}$ (%)
Trigger efficiency	0.03	0.03	0.05	0.05
Reconstruction efficiency	0.12	0.12	0.20	0.13
Identification efficiency	0.09	0.09	0.16	0.12
Forward identification efficiency	$\mathrm {-}$	$\mathrm {-}$	$\mathrm {-}$	1.51
Isolation efficiency	0.03	0.03	$\mathrm {-}$	0.04
Charge misidentification	0.04	0.06	$\mathrm {-}$	$\mathrm {-}$
Electron $p_{\text {T}}$ resolution	0.02	0.03	0.01	0.01
Electron $p_{\text {T}}$ scale	0.22	0.18	0.08	0.12
Forward electron $p_{\text {T}}$ scale + resolution	$\mathrm {-}$	$\mathrm {-}$	$\mathrm {-}$	0.18
$E_{\text {T}}^{\text {miss}}$ soft term scale	0.14	0.13	$\mathrm {-}$	$\mathrm {-}$
$E_{\text {T}}^{\text {miss}}$ soft term resolution	0.06	0.04	$\mathrm {-}$	$\mathrm {-}$
Jet energy scale	0.04	0.02	$\mathrm {-}$	$\mathrm {-}$
Jet energy resolution	0.11	0.15	$\mathrm {-}$	$\mathrm {-}$
Signal modelling (matrix-element generator)	0.57	0.64	0.03	1.12
Signal modelling (parton shower and hadronization)	0.24	0.25	0.18	1.25
PDF	0.10	0.12	0.09	0.06
Boson $p_{\text {T}}$	0.22	0.19	0.01	0.04
Multijet background	0.55	0.72	0.03	0.05
Electroweak+top background	0.17	0.19	0.02	0.14
Background statistical uncertainty	0.02	0.03	<0.01	0.04
Unfolding statistical uncertainty	0.03	0.04	0.04	0.13
Data statistical uncertainty	0.04	0.05	0.10	0.18
Total experimental uncertainty	0.94	1.08	0.35	2.29
Luminosity	1.8	1.8	1.8	1.8

The differential cross-section measurements as a function of the $W^{\pm }$ electron pseudorapidity and the dielectron rapidity and mass for the $Z/\gamma ^*$ channel are summarized in the Appendix in the Tables 23, 24, 25 and 26. The statistical uncertainties in the $W \rightarrow e \nu$ differential cross sections are about 0.1–0.2%, and the total uncertainties are in the range of 0.9–2.2%, excluding the luminosity uncertainty.

The differential $Z/\gamma ^* \rightarrow e^+e^-$ cross sections in the central region are measured in the $m_{ee}=66$–$116\,\text {GeV}$ invariant mass region with a statistical uncertainty of about 0.3–0.5% up to $|y_{\ell \ell }|=2.0$ and of 0.9% for $|y_{\ell \ell }| = 2.0$–2.4. The total uncertainty, excluding the luminosity uncertainty, is 0.5–0.7% up to $|y_{\ell \ell }|=2.0$ and $1.4\%$ for $|y_{\ell \ell }|= 2.0$–2.4. The statistical uncertainties of the differential $Z/\gamma ^* \rightarrow e^+e^-$ cross sections measured in the regions $m_{ee}=46$–$66\,\,\text {GeV}$ and 116–$150\,\text {GeV}$ are in the range 1.5–5%, dominating the total uncertainties of 2–6%.

The uncertainties in the forward $Z/\gamma ^* \rightarrow e^+e^-$ differential cross sections are dominated by systematic uncertainties. At the *Z* peak, the total uncertainty is 3–8%, while in the high-mass region it is about 10–20%.

#### Muon channels

**Fig. 10:**
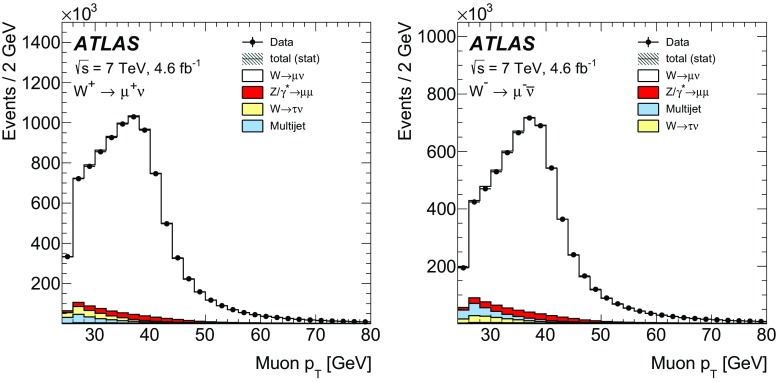
The transverse momentum distribution of muons for $W^+ \rightarrow \mu ^+ \nu$ candidates (*left*) and $W^- \rightarrow \mu ^- \bar{\nu }$ candidates (*right*). The simulated samples are normalized to the data luminosity. The multijet background shape is taken from a data control sample and normalized to the estimated yield of multijet events. The sum of all expected background and signal contributions is shown as a* solid line with a hashed band* detailing the statistical uncertainty and labelled “total (stat)”. The legend lists only background sources with a visible contribution

**Fig. 11:**
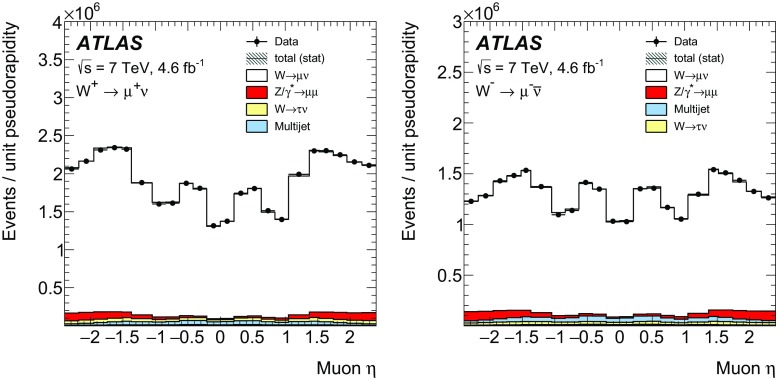
The pseudorapidity distribution of muons for $W^+ \rightarrow \mu ^+ \nu$ candidates (*left*) and $W^- \rightarrow \mu ^- \bar{\nu }$ candidates (*right*). The simulated samples are normalized to the data luminosity. The multijet background shape is taken from a data control sample and normalized to the estimated yield of multijet events. The sum of all expected background and signal contributions is shown as a* solid line with a hashed band* detailing the statistical uncertainty and labelled “total (stat)”. The legend lists only background sources with a visible contribution

**Fig. 12:**
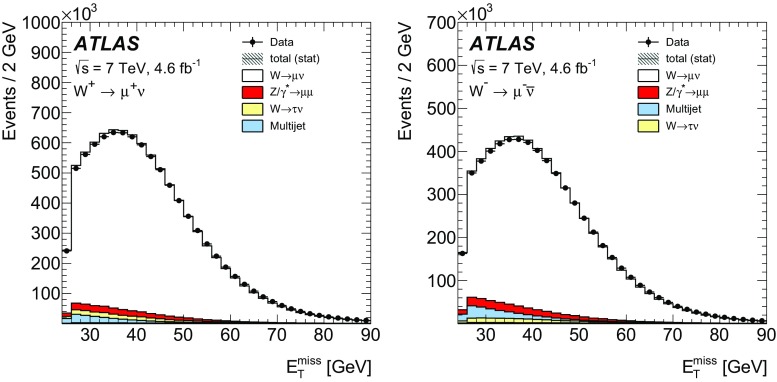
The missing transverse momentum distribution for $W^+ \rightarrow \mu ^+ \nu$ candidates (*left*) and $W^- \rightarrow \mu ^- \bar{\nu }$ candidates (*right*). The simulated samples are normalized to the data luminosity. The multijet background shape is taken from a data control sample and normalized to the estimated yield of multijet events. The sum of all expected background and signal contributions is shown as a* solid line with a hashed band* detailing the statistical uncertainty and labelled “total (stat)”. The legend lists only background sources with a visible contribution

**Fig. 13:**
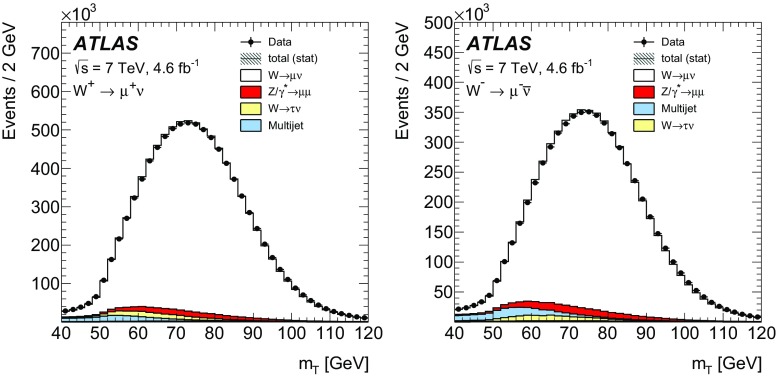
The transverse mass distribution for $W^+ \rightarrow \mu ^+ \nu$ candidates (*left*) and $W^- \rightarrow \mu ^- \bar{\nu }$ candidates (*right*). The simulated samples are normalized to the data luminosity. The multijet background shape is taken from a data control sample and normalized to the estimated yield of multijet events. The sum of all expected background and signal contributions is shown as a* solid line with a hashed band* detailing the statistical uncertainty and labelled “total (stat)”. The legend lists only background sources with a visible contribution

**Fig. 14:**
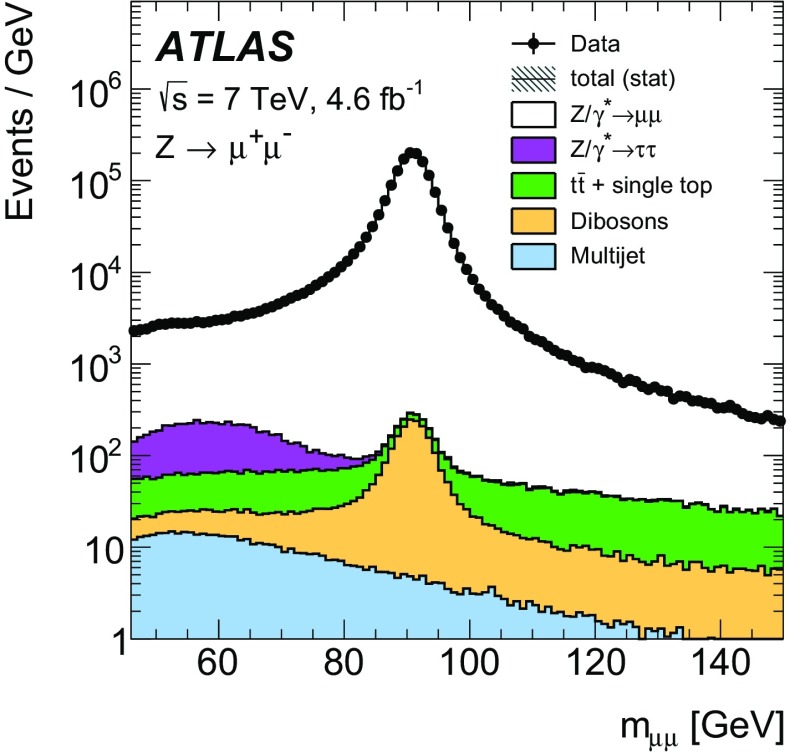
The dilepton invariant mass distributions for $Z/\gamma ^* \rightarrow \mu ^+\mu ^-$ candidates. The simulated samples are normalized to the data luminosity. The multijet background shape is taken from a data control sample and normalized to the estimated yield of multijet events. The sum of all expected background and signal contributions is shown as a* solid line with a hashed band* detailing the statistical uncertainty and labelled “total (stat)”. The legend lists only background sources with a visible contribution

**Fig. 15:**
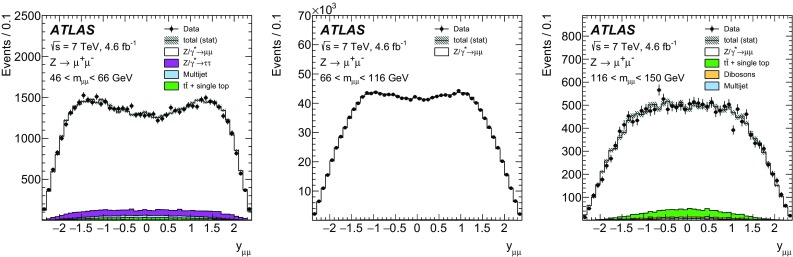
The dilepton rapidity distributions for $Z/\gamma ^* \rightarrow \mu ^+\mu ^-$ candidates in the mass regions $46< m_{\mu \mu }< 66\,\text {GeV}$ (*left*), $66< m_{\mu \mu }< 116\,\text {GeV}$ (*middle*) and $116< m_{\mu \mu }< 150\,\text {GeV}$ (*right*). The simulated samples are normalized to the data luminosity. The multijet background shape is taken from a data control sample and normalized to the estimated yield of multijet events. The sum of all expected background and signal contributions is shown as a* solid line with a hashed band* detailing the statistical uncertainty and labelled “total (stat)”. The legend lists only background sources with a visible contribution

The description of important kinematic variables in the muon-channel data by the signal simulation and the estimated backgrounds is illustrated in Figs. 10, 11, 12, 13, 14 and 15. The signal and electroweak background distributions are taken from MC simulation and normalized to the corresponding data luminosity. The distributions for the background from multijet production are obtained from data and normalized as described in Sect. 4.3. Figures 10, 11 and 12 show the distributions of muon transverse momentum, muon pseudorapidity and the missing transverse momentum of candidate *W* events for positive and negative charges. The transverse mass distributions are shown in Fig. 13. The dimuon mass distribution of muon pairs selected by the $Z/\gamma ^* \rightarrow \mu ^+\mu ^-$ analysis are shown in Fig. 14, while Fig. 15 shows the dimuon rapidity distributions for the three invariant mass regions. The level of agreement between data and simulation is good in all cases. Small disagreements in the shapes of the $E_{\text {T}}^{\text {miss}}$ and $m_\mathrm {T}$ distributions of W-boson candidates are visible in a similar way as in the electron channel and are covered by the systematic uncertainties.

Table 4 reports the number of candidates, the estimated background events and the $C_{W[Z]}$ correction factors used for the three different integrated muon channel measurements of the $W^+$, $W^-$, and $Z/\gamma ^*$ cross sections, the latter in the *Z*-peak region of $66<m_{\mu \mu }<116\,\,\text {GeV}$. The corresponding three integrated cross sections in the fiducial phase space specific to the muon channels are reported in Table 5 with their uncertainties due to data statistics, luminosity, and further experimental systematic uncertainties.

**Table 4:** Number of observed candidates *N*, of expected background events *B*, and the correction factors *C* for the $W^+$, $W^-$, and $Z/\gamma ^*$ ($66<m_{\mu \mu }<116\,\,\text {GeV}$) muon channels. The correction factors *C* were defined in Eq. (6). The charge asymmetry in the background to the $W^{\pm }$ channels stems from the $W \rightarrow \tau \nu$ contributions, which is proportional to the signal yield. The uncertainties are the quadratic sum of statistical and systematic components. The statistical uncertainties in *C* are negligible

	*N*	*B*	*C*
$W^+ \rightarrow \mu ^+ \nu$	9,225,887	$683{,}000 \pm 32{,}000$	$0.656 \pm 0.003$
$W^- \rightarrow \mu ^- \bar{\nu }$	6,260,198	$598{,}000 \pm 20{,}000$	$0.649 \pm 0.003$
$Z/\gamma ^* \rightarrow \mu ^+\mu ^-$	1,612,440	$6600 \pm 1200$	$0.734 \pm 0.003$

**Table 5:** Fiducial cross sections times branching ratios for $W^+$, $W^-$, and $Z/\gamma ^*$ ($66<m_{\mu \mu }<116\,\,\text {GeV}$) production in the muon decay channel. The fiducial regions used for the measurement are those defined for the combined fiducial regions in Sect. 2.3, except that the muon pseudorapidity is restricted to be within $|\eta |<2.4$. The uncertainties denote the statistical (stat), the systematic (syst), and the luminosity (lumi) uncertainties

	$$\sigma ^\mathrm {fid,\mu }_{W \rightarrow \mu \nu }$$ (pb)
$W^+ \rightarrow \mu ^+ \nu$	$~ 2839 \pm 1\,\mathrm {(stat)} \pm 17\,\mathrm {(syst)} \pm 51\,\mathrm {(lumi)}$
$W^- \rightarrow \mu ^- \bar{\nu }$	$~ 1901 \pm 1\,\mathrm {(stat)} \pm 11\,\mathrm {(syst)} \pm 34\,\mathrm {(lumi)}$

	$$\sigma ^\mathrm {fid,\mu }_{Z/\gamma ^* \rightarrow \mu \mu }$$ (pb)
$Z/\gamma ^* \rightarrow \mu ^+\mu ^-$	$~ 477.8 \pm 0.4\,\mathrm {(stat)} \pm 2.0\,\mathrm {(syst)} \pm 8.6\,\mathrm {(lumi)}$

The breakdown of the systematic uncertainty in all channels is shown in Table 6. Apart from the luminosity contribution of $1.8$%, the $W \rightarrow \mu \nu$ cross sections are measured with an experimental uncertainty of 0.6% and the $Z \rightarrow \mu ^+\mu ^-$ cross section is measured with an experimental uncertainty of 0.4%.

The uncertainties of the data-driven determinations of muon and hadronic recoil responses, discussed in Sect. 4.2, are propagated to the measurements. This comprises the uncertainties in the muon detection efficiencies, separated into contributions from the trigger, reconstruction, and isolation, which are relatively small for the $W \rightarrow \mu \nu$ channels and about 0.2% in total, but constitute the dominant systematic uncertainties in the $Z \rightarrow \mu ^+\mu ^-$ case with 0.34%. The muon $p_{\text {T}}$ resolution and scale uncertainties are very small for *Z* and subdominant for the $W \rightarrow \mu \nu$ channels at about 0.2%. The $W \rightarrow \mu \nu$ analyses are furthermore affected by uncertainties in the hadronic recoil response, decomposed into soft $E_{\text {T}}^{\text {miss}}$ and jet energy scale and resolution uncertainties, which add up to a total uncertainty contribution of about 0.2%.

Signal modelling variations with different event generators as discussed in Sect. 5.1 contribute uncertainties of about 0.1% to both the $W \rightarrow \mu \nu$ and $Z \rightarrow \mu ^+\mu ^-$ analyses. The high precision is achieved after a dedicated re-evaluation of the data-to-simulation correction factor for the muon isolation using alternative signal samples, which is especially relevant for the $Z \rightarrow \mu ^+\mu ^-$ peak data analysis, where the overlap of the samples used for efficiency calibration and cross-section analysis is very large. For the $W \rightarrow \mu \nu$ analysis, smaller effects from the multijet background determination and the hadronic recoil response remain. Other theoretical modelling uncertainties from PDFs and boson $p_{\text {T}}$ sources are also at the level of 0.1–0.2%.

The determination of uncertainties in the background subtraction follows the discussion in Sect. 4.3. The contribution of electroweak and top-quark backgrounds is about 0.2% for the $W \rightarrow \mu \nu$ analyses and much smaller for the *Z* analysis. With a contribution of about 0.3% the multijet background dominates the systematic uncertainty for the $W^+ \rightarrow \mu ^+ \nu$ and $W^- \rightarrow \mu ^- \bar{\nu }$ channels.

**Table 6:** Relative uncertainties $\delta \sigma$ in the measured integrated fiducial cross sections times branching ratios in the muon channels. The efficiency uncertainties are partially correlated between the trigger, reconstruction and isolation terms. This is taken into account in the computation of the total uncertainty quoted in the table

	$\delta \sigma _{W+}$ (%)	$\delta \sigma _{W-}$ (%)	$\delta \sigma _Z$ (%)
Trigger efficiency	0.08	0.07	0.05
Reconstruction efficiency	0.19	0.17	0.30
Isolation efficiency	0.10	0.09	0.15
Muon $p_{\text {T}}$ resolution	0.01	0.01	<0.01
Muon $p_{\text {T}}$ scale	0.18	0.17	0.03
$E_{\text {T}}^{\text {miss}}$ soft term scale	0.19	0.19	−
$E_{\text {T}}^{\text {miss}}$ soft term resolution	0.10	0.09	−
Jet energy scale	0.09	0.12	−
Jet energy resolution	0.11	0.16	−
Signal modelling (matrix-element generator)	0.12	0.06	0.04
Signal modelling (parton shower and hadronization)	0.14	0.17	0.22
PDF	0.09	0.12	0.07
Boson $p_{\text {T}}$	0.18	0.14	0.04
Multijet background	0.33	0.27	0.07
Electroweak+top background	0.19	0.24	0.02
Background statistical uncertainty	0.03	0.04	0.01
Unfolding statistical uncertainty	0.03	0.03	0.02
Data statistical uncertainty	0.04	0.04	0.08
Total experimental uncertainty	0.61	0.59	0.43
Luminosity	1.8	1.8	1.8

The differential cross-section measurements, as a function of the $W^+$ and $W^-$ muon pseudorapidity and of the dimuon rapidity and mass for the $Z/\gamma ^*$ channel, are summarized in Appendix in the Tables 27, 28 and 29. The statistical uncertainties in the $W \rightarrow \mu \nu$ differential cross sections are about 0.1–0.2%, and the total uncertainties are 0.6–0.9%, excluding the luminosity uncertainty.

The differential $Z/\gamma ^* \rightarrow \mu ^+\mu ^-$ cross sections are measured in the $m_{\mu \mu }=66$–$116\,\text {GeV}$ invariant mass region with a statistical uncertainty of about 0.3% up to $|y_{\ell \ell }|<2.0$ and of 0.8% for larger $|y_{\ell \ell }| < 2.4$. The total uncertainty, excluding the luminosity uncertainty, is 0.5% up to $|y_{\ell \ell }|<2.0$ and 1.0% for $|y_{\ell \ell }|=2.4$. The statistical uncertainties of the differential $Z/\gamma ^* \rightarrow \mu ^+\mu ^-$ cross sections measured in the $m_{\mu \mu }=46$–$66\,\,\text {GeV}$ and 116–$150\,\text {GeV}$ invariant mass regions are 1.3–4%, and the total uncertainties amount to 2–5%.

### Test of electron–muon universality

Ratios of the measured *W* and *Z* production cross sections in the electron and muon decay channels are evaluated from the corresponding measurements minimally extrapolated to the common fiducial phase space according to Eq. (8). These $e/\mu$ cross-section ratios represent direct measurements of the corresponding relative branching fractions, which are predicted to be unity in the SM given that lepton mass effects are negligible. Considering the case of the *W* boson, the ratio $R_W$ is obtained from the sum of $W^+$ and $W^-$ cross sections as:

$$\begin{aligned} R_{W}= & {} \frac{\sigma ^\mathrm {fid,e}_{W \rightarrow e\nu } /E_W^\mathrm {e}}{\sigma ^\mathrm {fid,\mu }_{W \rightarrow \mu \nu }/E_W^\mathrm {\mu }} = \frac{\sigma ^\mathrm {fid}_{W \rightarrow e\nu }}{\sigma ^\mathrm {fid}_{W \rightarrow \mu \nu }} =\frac{BR(W \rightarrow e\nu )}{BR(W \rightarrow \mu \nu )} \\= & {} 0.9967 \pm 0.0004\,\mathrm {(stat)} \pm 0.0101\,\mathrm {(syst)}\\= & {} 0.997 \pm 0.010. \end{aligned}$$

This measurement is more precise than the combination of LEP results from $e^+e^-\rightarrow W^+W^-$ data of $1.007 \pm 0.019$ [88]. It also significantly improves on the previous ATLAS measurements of $1.006 \pm 0.024$ with the 2010 data [1] and of $1.036 \pm 0.029$ with the 2015 data [7]. Related measurements were published by the CDF Collaboration with $R_W = 1.018 \pm 0.025$ [89] and recently by the LHCb Collaboration with $R_W = 1.020 \pm 0.019$ [14].

Similarly, the $e/\mu$ ratio of the *Z*-boson cross sections is extracted:

$$\begin{aligned} R_{Z}= & {} \frac{\sigma ^\mathrm {fid, e}_{Z \rightarrow ee}/E_Z^\mathrm {e}}{\sigma ^\mathrm {fid, \mu }_{Z \rightarrow \mu \mu }/E_Z^\mathrm {\mu }} = \frac{\sigma ^\mathrm {fid}_{Z \rightarrow ee}}{\sigma ^\mathrm {fid}_{Z \rightarrow \mu \mu }} = \frac{BR(Z \rightarrow ee)}{BR(Z \rightarrow \mu \mu )} \\= & {} 1.0026 \pm 0.0013\,\mathrm {(stat)} \pm 0.0048\,\mathrm {(syst)}\\= & {} 1.0026 \pm 0.0050. \end{aligned}$$

The result agrees well with the value obtained from the combination of $e^+e^-\rightarrow Z$ LEP and SLC data of $0.9991 \pm 0.0028$ [90]. It is significantly more precise than the previous ATLAS measurements: $1.018 \pm 0.031$ with the 2010 data [1] and $1.005 \pm 0.017$ with the 2015 data [7].

**Fig. 16:**
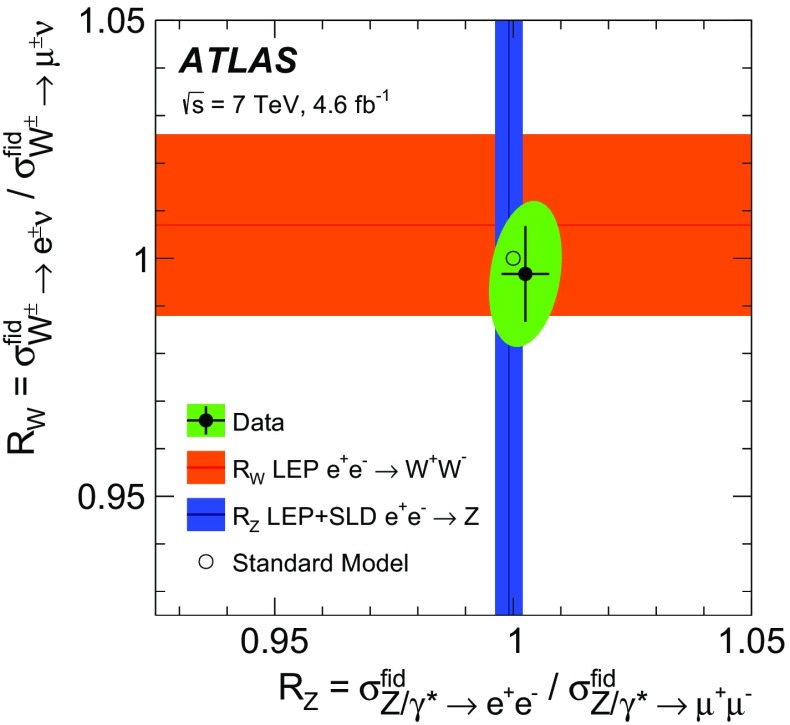
Measurement of the electron-to-muon cross-section ratios for the *W* and *Z* production, $R_W$ and $R_Z$. The *orange* and *blue*, *shaded bands* represent the combination of the ratios of electron and muon branching fractions for on-shell *W* and *Z* production as obtained at the $e^+e^-$ colliders LEP and SLC [88, 90]. The *green shaded ellipse* represents the 68% CL for the correlated measurement of $R_{W}$ and $R_{Z}$, while the black error bars give the one-dimensional standard deviation. The SM expectation of $R_W=R_Z=1$ is indicated with an *open circle*

The $R_W$ and $R_Z$ measurements therefore confirm lepton (*e*–$\mu$) universality in the weak vector-boson decays. The result, taking into account the correlations between the *W* and *Z* measurements, is illustrated in Fig. 16 as an ellipse. For comparison, bands are shown representing the above cited combined measurements from $e^+e^-$ colliders.

For the leptonic *W* branching fraction, $BR(W \rightarrow \ell \nu )$, precise constraints are also derived from off-shell *W* bosons in $\tau$-lepton, *K*-meson, and $\pi$-meson decays. For $\tau$ decays the HFAG group [91] obtains $R_W= (g_e/g_\mu )^2 = 0.9964 \pm 0.0028$, where $g_e$ and $g_\mu$ are the couplings of the *W* boson to *e* and $\mu$, respectively. The K$\,\text {TeV}$ measurement of $K\rightarrow \pi ^\pm \ell ^\mp \nu$ decays results in $R_W = 1.0031 \pm 0.0048$ [92]. The measurement of $K^\pm \rightarrow \ell ^\pm \nu$ decays by NA62 corresponds to an equivalent of $R_W = 1.0044 \pm 0.0040$ [93]. Finally, measurements of $\pi ^\pm \rightarrow \ell ^\pm \nu$ decays may be translated to a value of $R_W = 0.9992 \pm 0.0024$ [94].

### Combination of cross sections

#### Combination procedure

The $W^{\pm } \rightarrow \ell \nu$ and $Z/\gamma ^* \rightarrow \ell \ell$ cross-section measurements are performed in both the electron and muon decay channels. Assuming lepton universality, this provides a cross-check of experimental consistency and, as described later in this section, a means to improve the measurements when accounting for correlated and uncorrelated experimental uncertainties in the combination of the *e* and $\mu$ channel measurements. Correlations arise from the use of electrons, muons, or $E_{\text {T}}^{\text {miss}}$ reconstructed in the same way for different channels, but also due to similar or identical analysis techniques, e.g. in the background estimation. The method used to combine the cross-section data was also applied in the previous inclusive *W*,  *Z* cross-section measurement [1]. It was introduced for the combination of HERA cross-section measurements in Refs. [95, 96].

The combination procedure minimizes the deviation of the combined measurement $\sigma _{\mathrm {comb}}^i$ in a kinematic interval *i* from the input measurements $\sigma ^i_k$, where $k=1,2$ denotes the electron and muon measurements. This is achieved by allowing the contributions $b_j$ of the correlated uncertainty sources *j* to shift, where $b_j$ is expressed in units of standard deviations. The procedure requires as input a list of $\gamma _{j,k}^i$ values that specify the influence of the correlated uncertainty source *j* on the measurement *i* in the data set *k*. The relative data statistical and uncorrelated systematic uncertainties are given by $\delta ^i_{\mathrm {sta},k}$ and $\delta ^i_{\mathrm {unc},k}$, respectively. The resulting $\chi ^2$ function

11 $$\begin{aligned} \chi ^2(\vec {\sigma }_{\mathrm {comb}},\vec {b}) = \sum _{k,i} \frac{\left[ \sigma ^i_k - \sigma _{\mathrm {comb}}^i (1 - \sum _j \gamma ^i_{j,k}b_j) \right] ^2}{(\Delta ^i_k)^2} + \sum _{j}b_j^2 \end{aligned}$$

with

12 $$\begin{aligned} (\Delta ^i_k)^2=(\delta ^i_{\mathrm {sta},k})^2 \sigma ^i_k \sigma _{\mathrm {comb}}^i +(\delta ^i_{\mathrm {unc},k} \sigma _{\mathrm {comb}}^{i} )^{2} \end{aligned}$$

includes a penalty term for the systematic shifts $b_j$. The definition of $\Delta ^i_k$ ensures the minimization of biases due to statistical fluctuations, affecting the estimate of the statistical uncertainty, and treats systematic uncertainties in a multiplicative way [96]. Given the size of the statistical and systematic uncertainties for the data considered here, the differences between $\Delta ^i_k$ as used here and the simpler form without scaling are very small.

The uncertainties due to electron and muon momentum scales and resolutions are treated as fully correlated between the $W^{\pm } \rightarrow \ell \nu$ and $Z/\gamma ^* \rightarrow \ell \ell$ channels of a specific decay channel. Uncertainties in the hadronic recoil response, separated into jet and soft $E_{\text {T}}^{\text {miss}}$ scales and resolutions, only affect the $W^\pm$ channels and are treated in a correlated way between the $W^+$ and $W^-$ measurements and the *e* and $\mu$ channels.

The accurate determination of lepton selection efficiencies for online selection, reconstruction, identification, and isolation is an important input to the analysis. The efficiencies are measured in data and applied as correction factors to the simulation. These correction factors have statistical and procedural uncertainties, which are propagated to the measurements using pseudo-experiments for all channels in a consistent way. A covariance matrix is constructed from typically 1000 pseudo-experiments and then decomposed into a list of fully correlated uncertainty sources $\gamma$ and bin-to-bin uncorrelated uncertainties in the measurements.

The following theoretical uncertainties are largely correlated between all channels: (1) uncertainties in the measurements due to signal modelling, such as the boson transverse momentum spectrum; (2) theoretical uncertainties in signal modelling and hadronic recoil simulation, estimated with alternative signal samples, and (3) extrapolations applied to the measurements to account for the small differences in experimental fiducial phase spaces.

The uncertainties due to background estimation from simulated MC samples are treated as fully correlated between all channels, but separately for each background source. Data-driven background estimates are uncorrelated between channels and often contain significant statistical components, especially in the low-background $Z/\gamma ^* \rightarrow \ell \ell$ analyses. There is, however, a significant correlated part between $W^+$ and $W^-$ of a given lepton decay channel as the employed procedures are the same.

#### Integrated cross sections

The combination of fiducial integrated $Z/\gamma ^* \rightarrow \ell \ell$, $W^+ \rightarrow \ell ^+\nu$, and $W^- \rightarrow \ell ^-\bar{\nu }$ cross sections, including the full information contained in 66 correlated sources of uncertainty, gives a $\chi ^2$ per number of degrees of freedom ($\chi ^2/\mathrm {n.d.f.}$) of 0.5 / 3, indicating that the measurements are compatible. Table 7 summarizes the separate electron and muon channel measurements in the common fiducial volume and gives the final integrated fiducial cross-section results. Apart from the luminosity uncertainty of $1.8$%, a fiducial cross-section measurement precision of $0.32\%$ is reached for the NC channel and of $0.5\%~(0.6)\%$ for the $W^+$ ($W^-$) channels. The new *Z* (*W*) fiducial cross-section measurements are 10 (3.5) times more precise than the previous ATLAS measurements [1] when considering the statistical and systematic uncertainties added in quadrature.

**Table 7:** Integrated fiducial cross sections times leptonic branching ratios in the electron and muon channels and their combination with statistical and systematic uncertainties, for $W^+$, $W^-$, their sum and the $Z/\gamma ^*$ process measured at $\sqrt{s}=7\,\text {TeV}$. The $Z/\gamma ^*$ cross section is defined for the dilepton mass window $66<m_{\ell \ell }<116\,\,\text {GeV}$. The common fiducial regions are defined in Sect. 2.3. The uncertainties denote the statistical (stat), the experimental systematic (syst), and the luminosity (lumi) contributions

	$$\sigma ^\mathrm {fid}_{W \rightarrow \ell \nu }$$ (pb)
$W^+ \rightarrow e^+ \nu$	$2939 \pm 1 \,\mathrm {(stat)} \pm 28 \,\mathrm {(syst)} \pm 53 \,\mathrm {(lumi)}$
$W^+ \rightarrow \mu ^+ \nu$	$2948 \pm 1 \,\mathrm {(stat)} \pm 21 \,\mathrm {(syst)} \pm 53 \,\mathrm {(lumi)}$
$W^+ \rightarrow \ell ^+\nu$	$2947 \pm 1 \,\mathrm {(stat)} \pm 15 \,\mathrm {(syst)} \pm 53 \,\mathrm {(lumi)}$
$W^- \rightarrow e^- \bar{\nu }$	$1957 \pm 1 \,\mathrm {(stat)} \pm 21 \,\mathrm {(syst)} \pm 35 \,\mathrm {(lumi)}$
$W^- \rightarrow \mu ^- \bar{\nu }$	$1964 \pm 1 \,\mathrm {(stat)} \pm 13 \,\mathrm {(syst)} \pm 35 \,\mathrm {(lumi)}$
$W^- \rightarrow \ell ^-\bar{\nu }$	$1964 \pm 1 \,\mathrm {(stat)} \pm 11 \,\mathrm {(syst)} \pm 35 \,\mathrm {(lumi)}$
$W \rightarrow e \nu$	$4896 \pm 2\,\mathrm {(stat)} \pm 49 \,\mathrm {(syst)} \pm 88 \,\mathrm {(lumi)}$
$W \rightarrow \mu \nu$	$4912 \pm 1\,\mathrm {(stat)} \pm 32 \,\mathrm {(syst)} \pm 88 \,\mathrm {(lumi)}$
$W \rightarrow \ell \nu$	$4911 \pm 1\,\mathrm {(stat)} \pm 26 \,\mathrm {(syst)} \pm 88 \,\mathrm {(lumi)}$

	$$\sigma ^\mathrm {fid}_{Z/\gamma ^* \rightarrow \ell \ell }$$ (pb)
$Z/\gamma ^* \rightarrow e^+e^-$	$502.7 \pm 0.5 \,\mathrm {(stat)} \pm 2.0 \,\mathrm {(syst)} \pm 9.0 \,\mathrm {(lumi)}$
$Z/\gamma ^* \rightarrow \mu ^+\mu ^-$	$501.4 \pm 0.4 \,\mathrm {(stat)} \pm 2.3 \,\mathrm {(syst)} \pm 9.0\,\mathrm {(lumi)}$
$Z/\gamma ^* \rightarrow \ell \ell$	$502.2 \pm 0.3 \,\mathrm {(stat)} \pm 1.7 \,\mathrm {(syst)} \pm 9.0 \,\mathrm {(lumi)}$

Excluding the common luminosity uncertainty, the correlation coefficients of the $W^+$ and *Z*, $W^-$ and *Z*, and $W^+$ and $W^-$ fiducial cross-section measurements are 0.349,  0.314,  and 0.890, respectively. Including the luminosity, all three measurements are highly correlated, with coefficients of 0.964,  0.958 and 0.991, respectively. Table 8 presents four ratios that may be obtained from these fiducial integrated $Z/\gamma ^*$ and $W^\pm$ cross sections, where the luminosity uncertainty as well as other correlated uncertainties are eliminated or strongly reduced. The precision of these ratio measurements is very high with a total experimental uncertainty of $0.4\%$ for the $W^{+}/W^{-}$ ratio and $0.5\%$ for the $W^{\pm }/Z$ ratio.

**Table 8:** Ratios of integrated fiducial CC and NC cross sections obtained from the combination of electron and muon channels with statistical (stat) and systematic (syst) uncertainties. The common fiducial regions are defined in Sect. 2.3

$R^\mathrm {fid}_{W^{+}/W^{-}}$	$1.5006 \pm 0.0008\,\mathrm {(stat)} \pm 0.0037\,\mathrm {(syst)}$
$R^\mathrm {fid}_{W/Z}$	$9.780 \pm 0.006\,\mathrm {(stat)} \pm 0.049\,\mathrm {(syst)}$
$R^\mathrm {fid}_{W^{+}/Z}$	$5.869 \pm 0.004\,\mathrm {(stat)} \pm 0.029\,\mathrm {(syst)}$
$R^\mathrm {fid}_{W^{-}/Z}$	$3.911 \pm 0.003\,\mathrm {(stat)} \pm 0.021\,\mathrm {(syst)}$

In order to obtain the total cross sections, the combined integrated fiducial cross sections are also extrapolated to the full phase space with the procedure discussed in Sect. 5.1. Results are provided in Table 9. The uncertainties in these total cross sections receive significant contributions from PDF and signal modelling uncertainties, which are similar in size to the luminosity uncertainty. Ratios of these total cross sections are provided in Table 10. While for these ratios the luminosity uncertainty and a large part of the signal modelling uncertainties in the extrapolation are found to cancel, a significant uncertainty remains from PDF uncertainties.

**Table 9:** Total cross sections times leptonic branching ratios obtained from the combination of electron and muon channels with statistical and systematic uncertainties, for $W^+$, $W^-$, their sum and the $Z/\gamma ^*$ process measured at $\sqrt{s}=7\,\text {TeV}$. The $Z/\gamma ^*$ cross section is defined for the dilepton mass window $66<m_{\ell \ell }<116\,\,\text {GeV}$. The uncertainties denote the statistical (stat), the experimental systematic (syst), the luminosity (lumi), and acceptance extrapolation (acc) contributions

	$$\sigma ^\mathrm {tot}_{W \rightarrow \ell \nu }$$ (pb)
$W^+ \rightarrow \ell ^+\nu$	$6350 \pm 2\,\mathrm {(stat)} \pm 30\,\mathrm {(syst)} \pm 110\,\mathrm {(lumi)} \pm 100\,\mathrm {(acc)}$
$W^- \rightarrow \ell ^-\bar{\nu }$	$4376 \pm 2\,\mathrm {(stat)} \pm 25\,\mathrm {(syst)} \pm 79\,\mathrm {(lumi)} \pm 90\,\mathrm {(acc)}$
$W \rightarrow \ell \nu$	$10720 \pm 3\,\mathrm {(stat)} \pm 60\,\mathrm {(syst)} \pm 190\,\mathrm {(lumi)} \pm 130\,\mathrm {(acc)}$

	$$\sigma ^\mathrm {tot}_{Z/\gamma ^* \rightarrow \ell \ell }$$ (pb)
$Z/\gamma ^* \rightarrow \ell \ell$	$990 \pm 1\,\mathrm {(stat)} \pm 3\,\mathrm {(syst)} \pm 18\,\mathrm {(lumi)} \pm 15\,\mathrm {(acc)}$

**Table 10:** Ratios of total CC and NC cross sections obtained from the combination of electron and muon channels with statistical and systematic uncertainties. The $Z/\gamma ^*$ cross section is defined for the dilepton mass window $66<m_{\ell \ell }<116\,\,\text {GeV}$. The uncertainties denote the statistical (stat), the experimental systematic (syst), the luminosity (lumi), and acceptance extrapolation (acc) contributions

$R^\mathrm {tot}_{W^{+}/W^{-}}$	$1.450 \pm 0.001\,\mathrm {(stat)} \pm 0.004\,\mathrm {(syst)} \pm 0.029\,\mathrm {(acc)}$
$R^\mathrm {tot}_{W/Z}$	$10.83 \pm 0.01\,\mathrm {(stat)} \pm 0.05\,\mathrm {(syst)} \pm 0.09\,\mathrm {(acc)}$
$R^\mathrm {tot}_{W^{+}/Z}$	$6.407 \pm 0.004\,\mathrm {(stat)} \pm 0.032\,\mathrm {(syst)} \pm 0.062\,\mathrm {(acc)}$
$R^\mathrm {tot}_{W^{-}/Z}$	$4.419 \pm 0.003\,\mathrm {(stat)} \pm 0.024\,\mathrm {(syst)} \pm 0.082\,\mathrm {(acc)}$

#### Differential cross sections

For the combination of the rapidity-dependent differential cross sections, a simultaneous averaging of 105 data points, characterized by more than one hundred correlated sources from all channels, is performed leading to 61 combined measurement points. As the phase space regions of the central and forward $Z/\gamma ^* \rightarrow \ell \ell$ analyses are disjoint, and there is no $Z \rightarrow \mu ^+\mu ^-$ analysis in the forward region, the combination in this region is based solely on the $Z \rightarrow e^+e^-$ analysis. The forward $Z \rightarrow e^+e^-$ analysis is nevertheless included in the *e*–$\mu$ averaging to account for possible shifts and reductions of correlated uncertainties in a consistent way. Similarly, $W^\pm$ measurements in the bin $|\eta _{\ell }| \in [1.37, 1.52]$ are covered only by the muon channel.

**Fig. 17:**
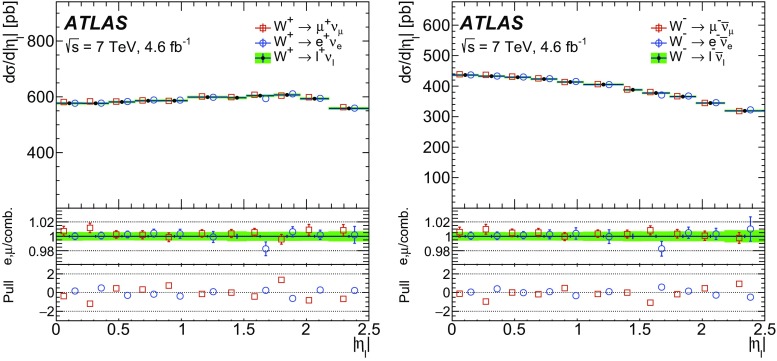
Differential $\mathrm {d}\sigma /\mathrm {d}|\eta _{\ell }|$ cross-section measurements for $W^+$ (*left*) and $W^-$ (*right*), for the electron channel (*open circles*), the muon channel (*open squares*) and their combination with uncorrelated uncertainties (*crosses*) and the total uncertainty, apart from the luminosity error (*green band*). Also shown are the ratios of the *e* and $\mu$ measurements to the combination and the pulls of the individual measurements in terms of their uncorrelated uncertainties, see text

**Fig. 18:**
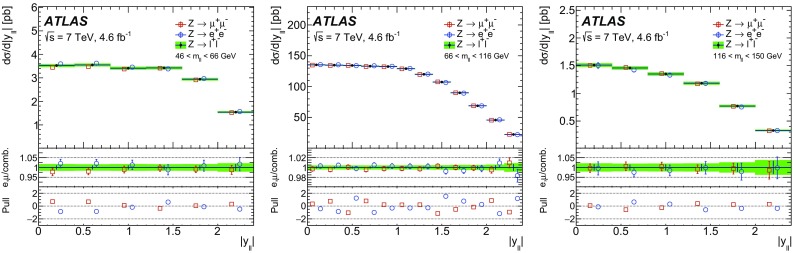
Differential $\mathrm {d}\sigma /\mathrm {d}|y_{\ell \ell }|$ cross-section measurements for $Z/\gamma ^{*} \rightarrow \ell \ell$ in the three $m_{\ell \ell }$ regions, for the electron channel (*open circles*), the muon channel (*open squares*) and their combination with uncorrelated uncertainties (*crosses*) and the total uncertainty, apart from the luminosity error (*green band*). Also shown are the ratios of the *e* and $\mu$ measurements to the combination and the pulls of the individual measurements in terms of their uncorrelated uncertainties, see text

The combination of the differential cross sections measured in the electron and muon channels is illustrated in Figs. 17 and 18 for the $W^{\pm } \rightarrow \ell \nu$ and $Z/\gamma ^* \rightarrow \ell \ell$ channels. The top panels show the measured muon and electron cross sections together with their combination. The central panel illustrates the $e/\mu$ ratio. The lowest panel shows the *pulls*, which are the deviations of the input measurements from the combination in terms of their uncorrelated uncertainties when fixing the systematic shifts $b_j$ at the values leading to the total $\chi ^2$ minimum.

The measurements in the electron and muon decay channels are compatible. This can be quantified with the total combination $\chi ^2/\mathrm {n.d.f.}$ of 47.2 / 44 and be inferred from the pulls displayed with Figs. 17 and 18. The partial $\chi ^2$ values are listed in Table 11 as well as the contribution of the penalty term constraining the shifts of correlated uncertainties .

**Table 11:** Partial and total $\chi ^2/\mathrm {n.d.f.}$ for the combination of the differential $\mathrm {d}\sigma /\mathrm {d}|\eta _{\ell }|$ and $\mathrm {d}\sigma /\mathrm {d}|y_{\ell \ell }|$ cross sections. The contribution of the penalty term constraining the shifts of correlated uncertainties is listed separately in the row labelled “Correlated”, see Eq. (11)

Channel	$\chi ^2/\mathrm {n.d.f.}$
$W^+ \rightarrow \ell ^+\nu$	6.7 / 10
$W^- \rightarrow \ell ^-\bar{\nu }$	4.5 / 10
$Z/\gamma ^* \rightarrow \ell \ell \ (46<m_{\ell \ell }<66\,\,\text {GeV})$	3.3 / 6
$Z/\gamma ^* \rightarrow \ell \ell \ (66<m_{\ell \ell }<116\,\,\text {GeV})$	15.2 / 12
$Z/\gamma ^* \rightarrow \ell \ell \ (116<m_{\ell \ell }<150\,\,\text {GeV})$	1.8 / 6
Correlated	15.7
Total	47.2 / 44

Apart from the common luminosity uncertainty of $1.8$%, the precision of the combined differential cross sections reaches 0.4–0.6% for the $W^+$ and $W^-$ as well as the central *Z* peak measurements. Off-peak and forward measurements have significantly larger uncertainties of typically a few percent but reaching as high as $20\%$. The differential combined measurement results are summarized in Tables 12, 13 and 14. The full measurement information is provided in HEPDATA. The measurements presented here supersede the results published in Ref. [1] because of their significantly higher precision and extended kinematic coverage.

**Table 12:** Differential cross section for the $W^+ \rightarrow \ell ^+\nu$ (top) and $W^- \rightarrow \ell ^-\bar{\nu }$ (bottom) processes, extrapolated to the common fiducial region. The relative statistical ($\delta _\mathrm {sta}$), uncorrelated systematic ($\delta _\mathrm {unc}$), correlated systematic ($\delta _\mathrm {cor}$), and total ($\delta _\mathrm {tot}$) uncertainties are given in percent of the cross-section values. The overall $1.8$% luminosity uncertainty is not included

$|\eta _{\ell }|^\mathrm {min}$	$|\eta _{\ell }|^\mathrm {max}$	$\mathrm {d}\sigma /\mathrm {d}|\eta _{\ell }|$ (pb)	$\delta _\mathrm {sta}$ (%)	$\delta _\mathrm {unc}$ (%)	$\delta _\mathrm {cor}$ (%)	$\delta _\mathrm {tot}$ (%)
$$W^+ \rightarrow \ell ^+\nu $$						
0.0	0.21	577.15	0.11	0.13	0.52	0.55
0.21	0.42	576.87	0.11	0.15	0.49	0.52
0.42	0.63	581.75	0.09	0.12	0.49	0.51
0.63	0.84	586.07	0.10	0.11	0.50	0.52
0.84	1.05	586.33	0.10	0.14	0.50	0.53
1.05	1.37	599.07	0.08	0.13	0.51	0.53
1.37	1.52	596.75	0.13	0.33	0.52	0.63
1.52	1.74	604.17	0.11	0.13	0.55	0.57
1.74	1.95	606.93	0.12	0.18	0.54	0.58
1.95	2.18	593.40	0.11	0.14	0.53	0.56
2.18	2.5	558.46	0.12	0.14	0.62	0.64
$$W^- \rightarrow \ell ^-\bar{\nu }$$						
0.0	0.21	436.45	0.12	0.14	0.52	0.55
0.21	0.42	432.78	0.12	0.16	0.48	0.52
0.42	0.63	429.29	0.11	0.13	0.49	0.52
0.63	0.84	423.38	0.12	0.13	0.50	0.53
0.84	1.05	413.64	0.11	0.15	0.50	0.54
1.05	1.37	405.26	0.10	0.14	0.56	0.59
1.37	1.52	388.02	0.17	0.34	0.52	0.64
1.52	1.74	377.51	0.14	0.16	0.58	0.62
1.74	1.95	365.82	0.12	0.20	0.58	0.63
1.95	2.18	344.70	0.13	0.17	0.59	0.63
2.18	2.5	319.04	0.14	0.19	0.75	0.79

**Table 13:** Differential cross section for the $Z/\gamma ^* \rightarrow \ell \ell$ process in the central region in three dilepton invariant mass regions, extrapolated to the common fiducial region. The relative statistical ($\delta _\mathrm {sta}$), uncorrelated systematic ($\delta _\mathrm {unc}$), correlated systematic ($\delta _\mathrm {cor}$), and total ($\delta _\mathrm {tot}$) uncertainties are given in percent of the cross-section values. The overall $1.8$% luminosity uncertainty is not included

$|y_{\ell \ell }|^\mathrm {min}$	$|y_{\ell \ell }|^\mathrm {max}$	$\mathrm {d}\sigma /\mathrm {d}|y_{\ell \ell }|$ (pb)	$\delta _\mathrm {sta}$ (%)	$\delta _\mathrm {unc}$ (%)	$\delta _\mathrm {cor}$ (%)	$\delta _\mathrm {tot}$ (%)
Central $Z/\gamma ^* \rightarrow \ell \ell$, $46<m_{\ell \ell }<66\,\,\text {GeV}$						
0.0	0.4	3.524	0.97	0.52	1.14	1.58
0.4	0.8	3.549	0.95	0.47	1.05	1.49
0.8	1.2	3.411	0.97	0.48	1.13	1.57
1.2	1.6	3.423	1.00	0.48	1.03	1.52
1.6	2.0	2.942	1.09	0.47	1.02	1.57
2.0	2.4	1.541	1.64	0.60	1.02	2.03
Central $Z/\gamma ^* \rightarrow \ell \ell$, $66<m_{\ell \ell }<116\,\,\text {GeV}$						
0.0	0.2	135.22	0.19	0.10	0.29	0.36
0.2	0.4	134.74	0.19	0.10	0.28	0.35
0.4	0.6	134.24	0.19	0.09	0.28	0.35
0.6	0.8	133.08	0.20	0.09	0.28	0.36
0.8	1.0	132.48	0.20	0.10	0.28	0.36
1.0	1.2	129.06	0.20	0.11	0.28	0.36
1.2	1.4	119.92	0.21	0.09	0.29	0.37
1.4	1.6	107.32	0.23	0.12	0.29	0.39
1.6	1.8	89.87	0.25	0.11	0.36	0.45
1.8	2.0	68.80	0.29	0.15	0.32	0.46
2.0	2.2	45.62	0.36	0.22	0.31	0.52
2.2	2.4	22.23	0.59	0.37	0.41	0.81
Central $Z/\gamma ^* \rightarrow \ell \ell$, $116<m_{\ell \ell }<150\,\,\text {GeV}$						
0.0	0.4	1.510	1.41	0.90	1.03	1.97
0.4	0.8	1.458	1.37	0.61	1.03	1.82
0.8	1.2	1.350	1.45	0.73	0.95	1.88
1.2	1.6	1.183	1.54	0.75	0.92	1.95
1.6	2.0	0.7705	2.03	0.99	1.06	2.49
2.0	2.4	0.3287	3.17	1.31	1.25	3.65

**Table 14:** Differential cross section for the $Z/\gamma ^* \rightarrow \ell \ell$ process in the forward region in two dilepton invariant mass ranges, extrapolated to the common fiducial region. The relative statistical ($\delta _\mathrm {sta}$), uncorrelated systematic ($\delta _\mathrm {unc}$), correlated systematic ($\delta _\mathrm {cor}$), and total ($\delta _\mathrm {tot}$) uncertainties are given in percent of the cross-section values. The overall $1.8$% luminosity uncertainty is not included

$|y_{\ell \ell }|^\mathrm {min}$	$|y_{\ell \ell }|^\mathrm {max}$	$\mathrm {d}\sigma /\mathrm {d}|y_{\ell \ell }|$ (pb)	$\delta _\mathrm {sta}$ (%)	$\delta _\mathrm {unc}$ (%)	$\delta _\mathrm {cor}$ (%)	$\delta _\mathrm {tot}$ (%)
Forward $Z/\gamma ^* \rightarrow \ell \ell$, $66<m_{\ell \ell }<116\,\,\text {GeV}$						
1.2	1.4	7.71	1.76	1.84	3.10	4.01
1.4	1.6	17.93	1.02	1.11	2.93	3.30
1.6	1.8	32.52	0.73	0.70	2.68	2.87
1.8	2.0	50.55	0.59	1.77	2.52	3.14
2.0	2.2	68.88	0.58	2.66	2.14	3.46
2.2	2.4	86.59	0.50	1.90	1.90	2.73
2.4	2.8	86.21	0.34	3.03	1.68	3.48
2.8	3.2	40.69	0.49	0.64	5.49	5.55
3.2	3.6	10.95	1.23	3.69	6.40	7.48
Forward $Z/\gamma ^* \rightarrow \ell \ell$, $116<m_{\ell \ell }<150\,\,\text {GeV}$						
1.2	1.6	0.300	6.84	6.58	8.96	13.06
1.6	2.0	0.548	5.21	7.78	7.20	11.81
2.0	2.4	0.925	3.99	13.52	4.26	14.72
2.4	2.8	0.937	3.87	20.86	3.87	21.57
2.8	3.2	0.437	5.30	14.40	6.59	16.70
3.2	3.6	0.0704	14.49	11.60	7.04	19.85

## Comparison with theory

### Theoretical framework and methodology

#### Drell–Yan cross-section predictions

Predictions for Drell–Yan production in proton–proton collisions in this paper are calculated at fixed order in perturbative QCD using the programs DYNNLO 1.5 [24, 25] and FEWZ  3.1.b2 [26–28]. Both programs calculate *W* and $Z/\gamma ^*$ boson production up to next-to-next-to-leading order in the strong coupling constant, $\mathcal {O}(\alpha _{\text {S}} ^2)$, and include the boson decays to leptons ($\ell ^+\nu$, $\ell ^-\bar{\nu }$, or $\ell ^+\ell ^-$) with full spin correlations, finite width, and interference effects. They allow kinematic phase-space requirements to be implemented for a direct comparison with experimental data. In addition, the programs ZWPROD [97] and VRAP [98] are available for total cross-section calculations enabling cross-checks or fast estimates of factorization and renormalization scale uncertainties.

At leading order (LO) in the electroweak (EW) couplings, there is a significant dependence of the cross-section predictions on the electroweak parameter scheme. For all calculations the $G_\mu$ scheme [99] is chosen, in which the primary parameters are the Fermi constant and the particle masses. Corrections for NLO EW effects reduce the dependence on the EW scheme and are important at the precision level required for the present measurements. These NLO EW corrections, however, require a separate treatment, discussed in Sect. 6.1.2, as they are currently not provided by the NNLO QCD programs, with the exception of the NC Drell–Yan calculation in FEWZ [28].

**Table 15:** Electroweak input parameters, in the $G_{\mu }$ scheme, for the NC and CC Drell–Yan *pp* and deep inelastic *ep* scattering cross-section calculations, see text. Standard Model parameters are taken from Refs. [39, 100], except $\Gamma (W \rightarrow \ell \nu )$. The $V_{ij}$ symbols denote the elements of the CKM matrix. The parameters below the line, the weak mixing angle $\sin ^2\theta _\mathrm {W}$, the fine-structure constant $\alpha _{G_{\mu }}$, and the vector couplings of up-type quarks $v_{u}$, down-type quarks $v_{d}$, and charged leptons $v_{\ell }$ to the *Z* boson, are calculated at tree level from the ones above

$m_Z$	91.1876 $\,\text {GeV}$	$|V_{ud}|$	0.97427
$\Gamma _Z$	2.4949 $\,\text {GeV}$	$|V_{us}|$	0.22534
$\Gamma (Z \rightarrow \ell \ell )$	0.08400 $\,\text {GeV}$	$|V_{ub}|$	0.00351
$m_W$	80.385 $\,\text {GeV}$	$|V_{cd}|$	0.22520
$\Gamma _W$	2.0906 $\,\text {GeV}$	$\vert V_{cs} \vert$	0.97344
$\Gamma (W \rightarrow \ell \nu )$	0.22727 $\,\text {GeV}$	$|V_{cb}|$	0.0412
$m_H$	125 $\,\text {GeV}$	$|V_{td}|$	0.00867
$m_t$	173.5 $\,\text {GeV}$	$|V_{ts}|$	0.0404
$G_\mathrm {F}$	$1.1663787 \times 10^{-5}$ $\,\text {GeV}$ $^{-2}$	$|V_{tb}|$	0.999146
$\sin ^2\theta _\mathrm {W}$	0.222897		
$\alpha _{G_{\mu }}$	$7.562396 \times 10^{-3}$		
$v_{u}$	0.405607		
$v_{d}$	−0.702804		
$v_{\ell }$	−0.108411		

The QCD analysis of the *ep* and *pp* data presented below assumes that the SM electroweak parameters are known. Their values are taken from the PDG [39], and are listed for reference in Table 15. The leptonic decay width of the *W* boson, $\Gamma (W \rightarrow \ell \nu )$, is an exception. The predicted value of $\Gamma (W \rightarrow \ell \nu )=226.36\,\text {MeV}$ quoted in the PDG effectively includes higher-order EW effects. For consistency with the higher-order EW corrections, provided by MCSANC [101], however, the leading-order partial width value, $\Gamma (W \rightarrow \ell \nu )=227.27\,\text {MeV}$, is used in both the QCD and EW calculations. It was verified that consistent results were obtained by using the PDG value and omitting the extra NLO EW corrections. For the leptonic decay width of the Z boson, the predicted value of $\Gamma (Z \rightarrow \ell \ell )=84.00\,\text {MeV}$ differs only by $0.1\%$ from the leading-order value of $\Gamma (Z \rightarrow \ell \ell )=83.92\,\text {MeV}$ and this difference is of no practical relevance for the NC Drell–Yan cross-section calculation. The values of the magnitudes of the CKM matrix elements, listed in Table 15, are taken from Ref. [100]. The $\vert V_{cs} \vert$ matrix parameter is accessible through $cs \rightarrow W$ production and thus related to the fraction of strange quarks in the proton, which is of special interest in this analysis. In Sect. 7.2.3 a dedicated QCD fit analysis is presented, where no prior knowledge is assumed on the magnitude of the CKM matrix element $\vert V_{cs} \vert$ , which instead is determined from the data together with the PDF parameters.

The nominal theoretical predictions of the differential, fiducial and total cross sections at NNLO in QCD are computed with DYNNLO 1.5 using the default program parameters.^2^ For an estimate of the current uncertainties of fixed-order perturbative QCD NNLO calculations, the DYNNLO  predictions are compared with predictions using FEWZ 3.1.b2. For the total cross sections, agreement to better than $0.2\%$ is observed. For the fiducial and differential cross-section measurements with additional kinematic requirements on the lepton transverse momenta and rapidities, however, poorer agreement is found: for the integrated fiducial $W^+,~W^-,~Z/\gamma ^*$ cross sections, the differences between FEWZ and DYNNLO predictions calculated with the ATLAS-epWZ12 PDF set amount to $(+1.2,\,+0.7,\,+0.2)\%$, which may be compared to the experimental uncertainties of $\pm (0.6,\,0.5,\,0.32)\%$, respectively^3^. See Ref. [102] for a further discussion of this effect.

In the calculation of the Drell–Yan cross sections, the renormalization and factorization scales, $\mu _\mathrm {r}$ and $\mu _\mathrm {f}$, are chosen to be the dilepton invariant mass, $m_{\ell \ell }$ , at the centre of the respective cross-section bin in the NC case and the *W*-boson mass, $m_W$, in the CC case. Variations of the scales by factors of 2 and 1 / 2 are conventionally used as an estimate of the approximation represented by NNLO as compared to still unknown higher-order corrections. The numerical implication of the scale choices, termed scale uncertainties, is considered in the evaluation of the QCD fit results on the strange-quark fraction and the CKM element $\vert V_{cs} \vert$. The DIS cross sections are calculated in all cases at the scale of $\mu _\mathrm {r}=\mu _\mathrm {f}= \sqrt{Q^2}$, where $Q^2$ denotes the negative square of the four-momentum transfer in NC and CC *ep* scattering.

The relative uncertainty of the LHC proton beam energy of $\pm 0.1\%$ [102] induces an uncertainty of the cross-section predictions of typically ${\pm } 0.1\%$, which is negligible compared to the other theoretical uncertainties discussed above.

#### Electroweak corrections and combination with QCD predictions

In Drell–Yan production, the dominant part of the higher-order electroweak corrections is the QED radiation from the final-state leptons. This contribution is included in the Drell–Yan MC samples using Photos [69] and then passed through the detailed ATLAS detector simulation as described in Sect. 2.2. The data are unfolded for QED FSR effects at the same time as for other detector effects. The calculations of the QED FSR effects by Photos and MCSANC 1.20 [103] agree very well [104]. The remaining NLO EW corrections are then calculated using MCSANC, excluding the QED FSR contributions, for both the NC and CC Drell–Yan processes. These terms include NLO contributions from initial-state photon radiation, EW loop corrections, and initial-state–final-state photon interference.

The NLO EW corrections calculated with MCSANC need to be combined with the NNLO QCD predictions, calculated with DYNNLO, to obtain complete predictions.^4^ This combination may be achieved using either a factorized or an additive approach [109]. A common PDF set at NNLO, ATLAS-epWZ12, is used for the calculation of both the absolute NNLO QCD and NLO EW cross sections. The combination of QCD and EW calculations in the factorized approach may be expressed using $K$-factor corrections as

13 $$\begin{aligned} \sigma _\mathrm {NNLO\;QCD}^\mathrm {NLO\;EW} = \sigma _\mathrm {NNLO\;QCD}^\mathrm {LO\;EW} \cdot K^\mathrm {EW} = \sigma _\mathrm {LO\;QCD}^\mathrm {LO\;EW} \cdot K_\mathrm {QCD} \cdot K^\mathrm {EW} \end{aligned}$$

with the electroweak $K^\mathrm {EW}$ and QCD $K_\mathrm {QCD}$ correction factors defined as

14 $$\begin{aligned} K_\mathrm {QCD } = \frac{\sigma _\mathrm {NNLO\;QCD}^\mathrm {LO\;EW}}{\sigma _\mathrm {LO\;QCD}^\mathrm {LO\;EW}} \;\;\;\;\text{ and } \;\;\;\; K^\mathrm {EW} = \frac{\sigma ^\mathrm {NLO\;EW}_\mathrm {LO\;QCD} }{\sigma _\mathrm {LO\;QCD}^\mathrm {LO\;EW}}. \end{aligned}$$

This assumes that the fractional higher-order EW corrections, quantified by $K^\mathrm {EW}$, are the same for all orders of QCD. They thus can be determined based on LO QCD Drell–Yan cross-section calculations.

The alternative additive approach assumes the absolute contribution of the EW correction to be independent of the order of the underlying QCD calculation. Thus the relative fraction of the higher-order EW corrections is different for each order of QCD by $(K^\mathrm {EW}-1)/K_\mathrm {QCD}$. The combination of QCD and EW calculations then proceeds as

15 $$\begin{aligned} \sigma _\mathrm {NNLO\;QCD}^\mathrm {NLO\;EW} = \sigma _\mathrm {NNLO\;QCD}^\mathrm {LO\;EW} + \left( \sigma ^\mathrm {NLO\;EW}_\mathrm {LO\;QCD} -\sigma _\mathrm {LO\;QCD}^\mathrm {LO\;EW} \right) =\sigma _\mathrm {NNLO\;QCD}^\mathrm {LO\;EW} \cdot \left( 1+\frac{K^\mathrm {EW}-1}{K_\mathrm {QCD}} \right) . \end{aligned}$$

The central value of the combined NNLO QCD and NLO EW prediction is taken from the additive approach, which is also implemented in FEWZ [28]. The corrections to be applied to the NNLO QCD fiducial cross sections according to Eq. (15) are about −0.4 and −0.3% for the $W^+$ and $W^-$ channels, respectively. For the neutral-current channels, those corrections are $+6\%$, $-0.3\%\,(-0.4\%)$ and $-0.5\%\,(-1.2\%)$ for the central (forward) selection in the low-mass, *Z*-peak and high-mass regions of $m_{\ell \ell }$, respectively. The corrections are calculated separately for each measurement bin, but they depend only weakly on $\eta _{\ell }$ and $y_{\ell \ell }$ for the CC and NC case, respectively.

The differences between the additive and factorized approaches are in general found to be small and significantly smaller than the experimental uncertainty of the results presented in this paper. They are at most 0.3–0.9% for the low-mass $m_{\ell \ell }= 46$–$66\,\,\text {GeV}$ region for the NC case with larger effects observed at central rapidity. In the forward *Z*-peak phase space, they extend to $0.4\%$. In all other regions of phase space, the effect is ${<}0.1\%$. These differences are taken as a systematic uncertainty applied symmetrically to the central value obtained using the additive approach.

Additional two-loop EW corrections for the leading contributions are calculated using MCSANC for the NC case [110]. This correction is found to be ${<}0.1\%$ everywhere except for the region $m_{\ell \ell }= 46$–$66\,\text {GeV}$, where it reaches $(-0.62 \pm 0.15)\%$.

The radiation of real (on-shell) *W* and *Z* bosons is very small for the considered phase space [111] and neglected. An important background to the NC process outside the *Z*-boson mass region arises from photon-induced dileptons, $\gamma \gamma \rightarrow \ell \ell$. This contribution is calculated including NLO effects for the fiducial phase space with the MCSANC [103] program and subtracted from the unfolded data. The calculation uses the average of the two available MRST2004qed [112] predictions for the photon PDF as the central value and half the difference as an uncertainty estimate. The size of the photon-induced contribution is about $1.5\%$ in the low and high $m_{\ell \ell }$ bins, while it is negligible (${<}0.1\%$) at the *Z* peak. Due to large uncertainties on the photon PDF, the fractional uncertainties are at the level of 30–50%.

#### Methodology of PDF profiling

The impact of new data on a given PDF set can be estimated in a quantitative way with a profiling procedure [36, 37]. The profiling is performed using a $\chi ^2$ function which includes both the experimental uncertainties and the theoretical ones arising from PDF variations:

16 $$\begin{aligned} \chi ^2(\mathbf {b}_{\mathrm {exp}},\mathbf {b}_{\mathrm {th}})= & {} \sum _{i=1}^{N_\mathrm {data}} \frac{\textstyle \left[ \sigma ^\mathrm {exp}_i - \sigma ^\mathrm {th}_i (1 - \sum _j \gamma ^\mathrm {exp}_{ij} b_{j,\mathrm {exp}} - \sum _k \gamma ^\mathrm {th}_{ik}b_{k,\mathrm {th}}) \right] ^2}{\Delta _i^2} \nonumber \\&+\, \sum _{j=1}^{N_\mathrm {exp. sys}} b_{j,\mathrm {exp}}^2 + \sum _{k=1}^{N_\mathrm {th. sys}} b_{k,\mathrm {th}}^2. \end{aligned}$$

This $\chi ^2$ function resembles the one used for the combination, described in Sect. 5.4. The index *i* runs over all $N_\mathrm {data}$ data points. The measurements and the theory predictions are given by $\sigma ^\mathrm {exp}_i$ and $\sigma _i^\mathrm {th}$, respectively. The correlated experimental and theoretical uncertainties are included using the nuisance parameter vectors $\mathbf {b}_{\mathrm {exp}}$ and $\mathbf {b}_{\mathrm {th}}$, respectively. Their influence on the data and theory predictions is described by the matrices $\gamma ^\mathrm {exp}_{ij}$ and $\gamma ^\mathrm {th}_{ik}$, where the index *j* (*k*) corresponds to the $N_\mathrm {exp. sys}$ experimental ($N_\mathrm {th. sys}$ theoretical) nuisance parameters. Both the correlated and uncorrelated systematic uncertainties are treated as multiplicative. The estimation of the statistical uncertainties is protected against statistical fluctuations in data using the expected rather than the observed number of events and the denominator is hence calculated as

17 $$\begin{aligned} \Delta _i^2=\delta _{i,\mathrm {sta}}^2\sigma ^\mathrm {exp}_i \sigma ^\mathrm {th}_i + (\delta _{i,\mathrm {unc}}\sigma ^\mathrm {th}_i)^2. \end{aligned}$$

The contribution to the $\chi ^2$ from the two sums over $b_{j,k}^2$, which implement the $\pm 1\sigma$ constraints of the nuisance parameters, is later also referred to as the “correlated” contribution. The $\chi ^2$ function of Eq. (16) can be generalized to account for asymmetric uncertainties, as described in Ref. [37].

The value of the $\chi ^2$ function at its minimum provides a compatibility test of the data and theory. In addition, the values of the nuisance parameters at this minimum, $b^\mathrm {min}_{k,\mathrm {th}}$, can be interpreted as an optimization (“profiling”) of PDFs to describe the data [36]. The profiled central PDF set $f'_0$ is given by

18 $$\begin{aligned} f'_0 = f_0 + \sum _k \left[ b^\mathrm {min}_{k, \mathrm {th}} \left( \frac{f^{+}_k - f^{-}_k}{2} \right) + \left( b^\mathrm {min}_{k, \mathrm {th}}\right) ^2 \left( \frac{f^{+}_k + f^{-}_k - 2f_0}{2} \right) ^2\right] , \end{aligned}$$

where $f_0$ is a short notation for the original central PDFs of each parton flavour, $f_0 = xf(x,Q^2)$, and $f^{\pm }_k$ represent the eigenvector sets corresponding to up and down variations. For the LHAPDF6 [84] parameterizations, $f_0$ and $f^{\pm }_k$ are given as data tables at fixed $x,Q^2$ grid points. Equation (18) is a parabolic approximation of the PDF dependence close to the central value, e.g. for a single nuisance parameter, taking the values $b_\mathrm {th} = +1,~-1,~0$, the values of $f'_0$ are $f'_0 = f^+,~f^-,~f_0$, respectively.

The profiled PDFs $f'_0$ have reduced uncertainties. In general, the shifted eigenvectors are no longer orthogonal and are transformed to an orthogonal representation using a standard procedure [96], which can be extended to asymmetric uncertainties. The profiling procedure used in this analysis is implemented in the xFitter package [113]. The $\chi ^2$ function used in the analysis takes into account asymmetric PDF uncertainties.

The profiling procedure quantifies the compatibility of a data set with the predictions based on a PDF set and estimates the PDF sensitivity of the data set. However, the results of profiling are only reliable when the prediction is broadly consistent with the data within the PDF uncertainties because of the approximation involved in Eq. (18), and the profiling cannot act as a substitute for a full QCD fit analysis. A second caveat is that the $\chi ^2$ tolerance criteria, which many global PDF analyses use [114], are different from the $\Delta \chi ^2=1$ employed in the profiling. Thus the impact of the data in a full PDF fit pursued by those groups may differ from the result of a profiling analysis as outlined here. Profiling results are presented below for the PDF sets ABM12, CT14, MMHT2014, NNPDF3.0 (Hessian representation [115]), and ATLAS-epWZ12.

### Integrated cross sections and their ratios

**Fig. 19:**
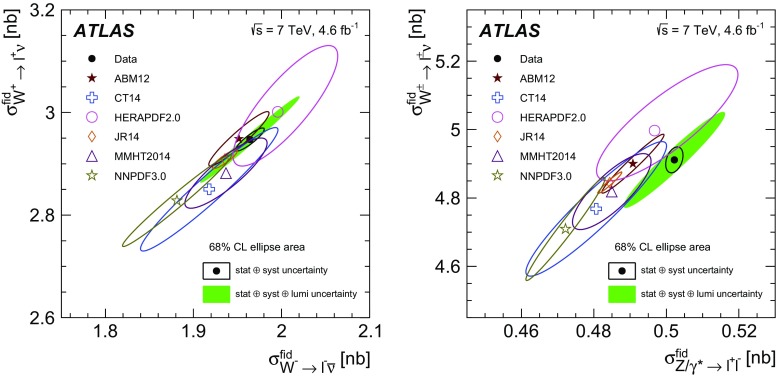
Integrated fiducial cross sections times leptonic branching ratios of $\sigma ^\mathrm {fid}_{W^+\rightarrow \ell ^+\nu }$ vs. $\sigma ^\mathrm {fid}_{W^-\rightarrow \ell ^-\bar{\nu }}$ (*left*) and $\sigma ^\mathrm {fid}_{W^\pm \rightarrow \ell ^\pm \nu }$ vs. $\sigma ^\mathrm {fid}_{Z/\gamma ^{*}\rightarrow \ell ^+\ell ^-}$ (*right*). The data ellipses illustrate the 68% CL coverage for the total uncertainties (*full green*) and total excluding the luminosity uncertainty (*open black*). Theoretical predictions based on various PDF sets are shown with *open symbols of different colours*. The uncertainties of the theoretical calculations correspond to the PDF uncertainties only

The combined integrated cross sections in the fiducial phase space are shown in Fig. 19. NNLO QCD predictions with NLO EW corrections based on the ABM12, CT14, HERAPDF2.0, JR14, MMHT2014, NNPDF3.0 PDF sets are compared to the data. The central values and their uncertainties for these PDF sets are provided in Table 16 together with the combined measurements reported before in Table 7.

**Table 16:** Predictions at NNLO QCD and NLO EW as obtained with DYNNLO 1.5 for the integrated fiducial cross sections. The given uncertainties correspond to PDF uncertainties only and are evaluated following the different prescriptions of the PDF groups. The measured cross sections as reported before in Table 7 are shown in the last row with their total uncertainties

PDF set	$\sigma ^\mathrm {fid}_{W^+\rightarrow \ell ^+\nu }$ (pb)	$\sigma ^\mathrm {fid}_{W^-\rightarrow \ell ^-\bar{\nu }}$ (pb)	$\sigma ^\mathrm {fid}_{W^\pm \rightarrow \ell ^\pm \nu }$ (pb)	$\sigma ^\mathrm {fid}_{Z/\gamma ^*\rightarrow \ell \ell }$ (pb)
ABM12	$2949 \pm 35$	$1952 \pm 23$	$4900 \pm 57$	$490.8 \pm 5.7$
CT14	$2850^{+77}_{-82}$	$1918^{+46}_{-57}$	$4770^{+120}_{-140}$	$481^{+11}_{-14}$
HERAPDF2.0	$3001^{+89}_{-66}$	$1996^{+48}_{-31}$	$5000^{+140}_{-90}$	$497^{+16}_{-9}$
JR14	$2909^{+13}_{-11}$	$1936^{+10}_{-9}$	$4845^{+23}_{-19}$	$484.4 \pm 2.2$
MMHT2014	$2882^{+49}_{-42}$	$1937^{+30}_{-32}$	$4819^{+75}_{-72}$	$485^{+7.4}_{-6.9}$
NNPDF3.0	$2828 \pm 59$	$1881 \pm 41$	$4709 \pm 99$	$472.2 \pm 7.2$
Data	$2947 \pm 55$	$1964 \pm 37$	$4911 \pm 92$	$502.2 \pm 9.2$

The two-dimensional presentation is particularly instructive, as it conveys both the values and correlations of both the measurements and predictions. The cross-section calculations are performed with the DYNNLO program as described in Sect. 6.1. All experimental and theoretical ellipses are defined such that their area corresponds to $68\%$ CL.^5^

Correlations between the predicted cross sections are evaluated from individual error eigenvectors in each PDF set. The spread of the predictions as well as the size of the individual PDF uncertainties are significantly larger than the uncertainty of the data. The measurements are seen to discriminate between different PDF choices and to provide information to reduce PDF uncertainties. As seen in Fig. 19, the PDF sets CT14, MMHT2014 and NNPDF3.0 give predictions that are lower for both the $W^+$ and the $W^-$ cross sections, a trend that is also observed for the $Z/\gamma ^*$ cross section.

**Fig. 20:**
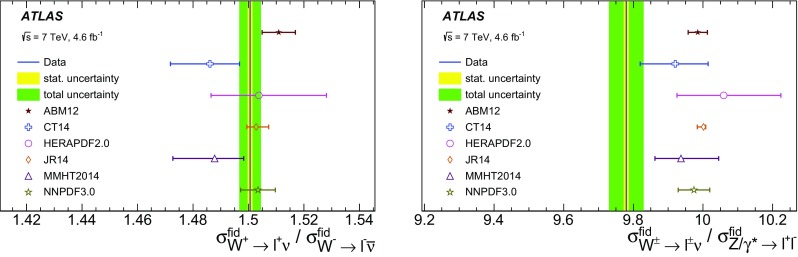
Ratios of the fiducial cross sections times leptonic branching ratios of $\sigma ^\mathrm {fid}_{W^+\rightarrow \ell ^+\nu }/\sigma ^\mathrm {fid}_{W^-\rightarrow \ell ^-\bar{\nu }}$ (*left*) and $\sigma ^\mathrm {fid}_{W^\pm \rightarrow \ell ^\pm \nu }/\sigma ^\mathrm {fid}_{Z/\gamma ^{*}\rightarrow \ell ^+\ell ^-}$ (*right*). The data (*solid blue line*) are shown with the statistical (*yellow band*) and the total uncertainties (*green band*). Theoretical predictions based on various PDF sets are shown with *open symbols of different colours*. The uncertainties of the theoretical calculations correspond to the PDF uncertainties only

The ratios of the combined fiducial cross sections, presented before in Table 8, are compared in Fig. 20 to NNLO QCD predictions based on various PDF sets. It is observed that the measured $W^+/W^-$ ratio is well reproduced, but, as already seen in the correlation plots above, all PDF sets predict a higher *W* / *Z* ratio than measured in the data.

### Rapidity distributions

#### $W^+$ and $W^-$ cross sections

**Fig. 21:**
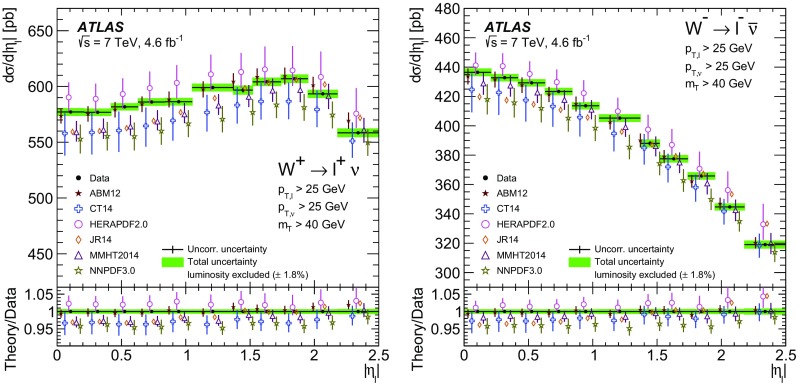
Differential $\mathrm {d}\sigma _{W+}/\mathrm {d}|\eta _{\ell }|$ (*left*) and $\mathrm {d}\sigma _{W-}/\mathrm {d}|\eta _{\ell }|$ (*right*) cross-section measurement for $W \rightarrow \ell \nu$. Predictions computed at NNLO QCD with NLO EW corrections using various PDF sets (*open symbols*) are compared to the data (*full points*). The ratio of theoretical predictions to the data is also shown. The predictions are displaced within each bin for better visibility. The theory uncertainty corresponds to the quadratic sum of the PDF uncertainty and the statistical uncertainty of the calculation

Differential cross sections as a function of lepton pseudorapidity in $W \rightarrow \ell \nu$ decays, for both $W^+$ and $W^-$, are shown in Fig. 21 and compared to NNLO perturbative QCD predictions, including NLO EW corrections. The predictions with the ABM12 PDF set match the data particularly well, while the predictions of NNPDF3.0, CT14, MMHT14 and JR14, tend to be below and the HERAPDF2.0 set slightly above the *W* cross-section data. For many PDF sets, the differences, however, do not exceed the luminosity uncertainty of $1.8\%$ by a significant amount. Different groups producing PDF sets make different choices in their evaluation of uncertainties. For example, the JR14 set is less consistent with these data even though it is somewhat closer to the data than the NNPDF3.0 set, which quotes much larger uncertainties than JR14.

The measurements of $W^{+}$ and $W^{-}$ cross sections as a function of $\eta _{\ell }$ are used to extract the lepton charge asymmetry

19 $$\begin{aligned} A_{\ell } = \frac{\mathrm {d}\sigma _{W+}/\mathrm {d}|\eta _{\ell }| - \mathrm {d}\sigma _{W-}/\mathrm {d}|\eta _{\ell }|}{\mathrm {d}\sigma _{W+}/\mathrm {d}|\eta _{\ell }| + \mathrm {d}\sigma _{W-}/\mathrm {d}|\eta _{\ell }|} , \end{aligned}$$

taking into account all sources of correlated and uncorrelated uncertainties.

**Fig. 22:**
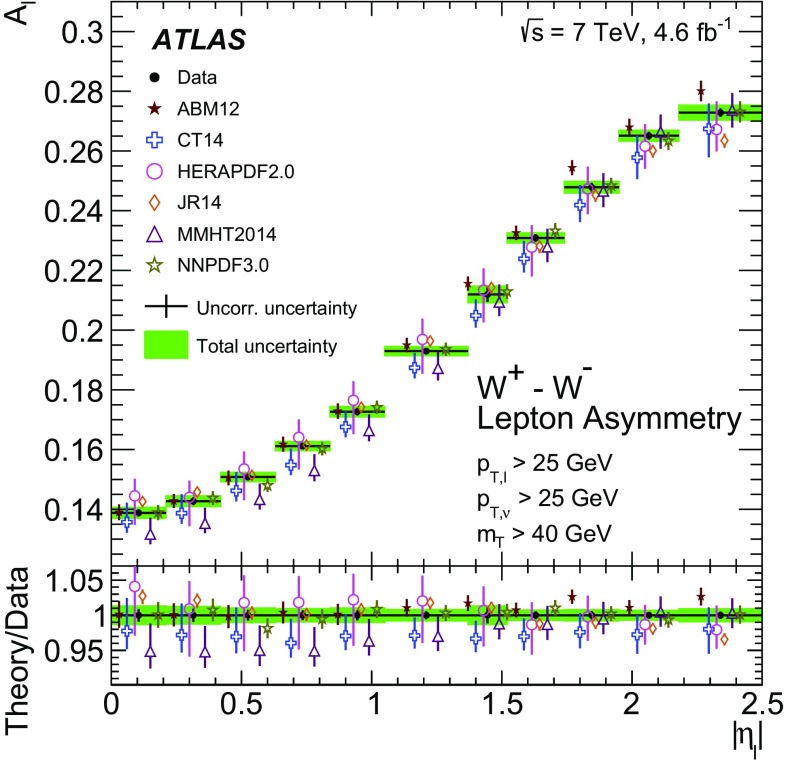
Lepton charge asymmetry $A_{\ell }$ in $W \rightarrow \ell \nu$ production as a function of the lepton pseudorapidity $|\eta _{\ell }|$. Predictions computed at NNLO QCD with NLO EW corrections using various PDF sets (*open symbols*) are compared to the data (*full points*). The ratio of theoretical predictions to the data is also shown. The predictions are displaced within each bin for better visibility. The theory uncertainty corresponds to the quadratic sum of the PDF uncertainty and the statistical uncertainty of the calculation

Figure 22 shows the measured charge asymmetry and the predictions based on various PDF sets. The experimental uncertainty ranges from 0.5 to 1%. Most of the predictions agree well with the asymmetry measurement, only CT14 somewhat undershoots the data. The NNPDF3.0 set, which uses $W^{\pm }$ asymmetry data from the CMS Collaboration [19, 20], matches the ATLAS data very well, even within its very small uncertainties. On the other hand, these predictions are in general 3–5% below both the measured $W^+$ and $W^-$ differential cross sections. This highlights the additional information provided by precise, absolute differential measurements with full uncertainty information, including the correlations, as compared to an asymmetry measurement.

#### $Z/\gamma ^*$ cross sections

**Fig. 23:**
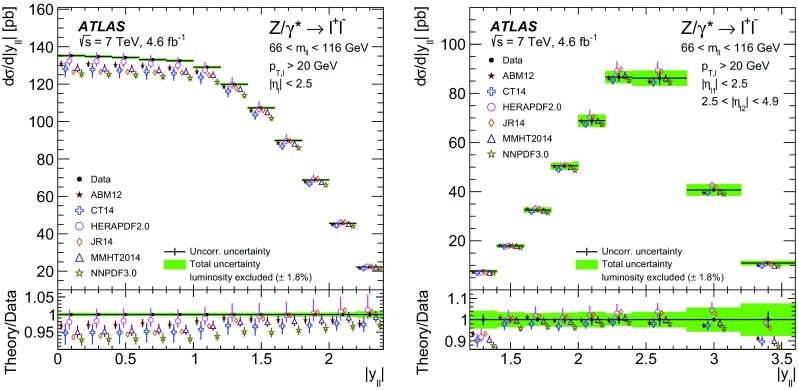
Differential cross-section measurement $\mathrm {d}\sigma /\mathrm {d}|y_{\ell \ell }|$ for $Z/\gamma ^* \rightarrow \ell \ell$ in the *Z*-peak region, $66< m_{\ell \ell }< 116\,\,\text {GeV}$, for central (*left*) and forward rapidity values (*right*). Predictions computed at NNLO QCD with NLO EW corrections using various PDF sets (*open symbols*) are compared to the data (*full points*). The ratio of theoretical predictions to the data is also shown. The predictions are displaced within each bin for better visibility. The theory uncertainty corresponds to the quadratic sum of the PDF uncertainty and the statistical uncertainty of the calculation

**Fig. 24:**
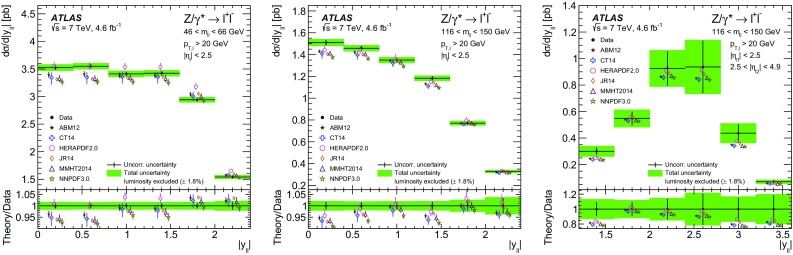
Differential cross-section measurement $\mathrm {d}\sigma /\mathrm {d}|y_{\ell \ell }|$ for $Z/\gamma ^* \rightarrow \ell \ell$ in the central-rapidity low-mass region (*left*), the central-rapidity high-mass region (*middle*), and the forward-rapidity high-mass region (*right*). Predictions computed at NNLO QCD with NLO EW corrections using various PDF sets (*open symbols*) are compared to the data (*full points*). The ratio of theoretical predictions to the data is also shown. The predictions are displaced within each bin for better visibility. The theory uncertainty corresponds to the quadratic sum of the PDF uncertainty and the statistical uncertainty of the calculation

Differential $Z/\gamma ^* \rightarrow \ell \ell$ cross-sections, as a function of the dilepton rapidity, are shown in Figs. 23 and 24, and compared to NNLO perturbative QCD predictions, including NLO EW corrections. The predictions are evaluated with various PDF sets. At the *Z* peak, where the highest precision is reached for the data, all predictions are below the data at central rapidity, $|y_{\ell \ell }| < 1$, but least for the HERAPDF2.0 set, which quotes the largest uncertainties. In the forward region, the PDFs agree well with the measurement, which, however, is only precise to the level of a few percent and thus not very sensitive to differences between PDFs. In the low mass $Z/\gamma ^* \rightarrow \ell \ell$ region, Fig. 24, several of the PDF sets exhibit a different rapidity dependence than the data although being mostly consistent with the measurement. This also holds for the central rapidity region at high mass, $116< m_{\ell \ell }< 150\,\text {GeV}$. The precision of the data in the forward region at high mass is too low to allow discrimination between the various PDF sets, all of which reproduce the measured rapidity dependence within the quoted uncertainties.

### PDF profiling results

Using the profiling technique introduced in Sect. 6.1, the agreement between data and predictions can be quantitatively assessed. Table 17 provides $\chi ^2/\mathrm {n.d.f.}$ values for each Drell–Yan data set and a number of PDFs, taking into account the experimental uncertainties, and also including the uncertainties provided by the individual PDF sets. Including the full PDF uncertainties, a satisfactory description of the data is achieved with the CT14 PDFs, where the $\chi ^2/\mathrm {n.d.f.}$ is similar to the dedicated PDF analysis presented in Sect. 7.^6^ The predictions with the MMHT14 and ATLAS-epWZ12 sets have a total $\chi ^2$ increased by about ten units compared to CT14, while the ABM12 and NNPDF3.0 predictions exhibit a larger tension with the data. The poorer description of the $Z/\gamma ^* \rightarrow \ell \ell$ data in the low mass region $m_{\ell \ell }=46$–$66\,\,\text {GeV}$ may reflect the enhanced theoretical uncertainties below the *Z* peak, which are not included in the $\chi ^2$ calculation.

**Table 17:** Values of $\chi ^2$ for the predictions using various PDF sets split by data set with the respective number of degrees of freedom ($\mathrm {n.d.f.}$). The contribution of the penalty term constraining the shifts of experimental and theoretical correlated uncertainties is listed separately in the row labelled “Correlated $\chi ^2$”, see Eq. (16). The values to the left (right) of the vertical line refer to $\chi ^2$ when the PDF uncertainties are included (excluded) in the evaluation

Data set	n.d.f.	ABM12	CT14	MMHT14	NNPDF3.0	ATLAS-epWZ12
$W^+ \rightarrow \ell ^+\nu$	11	11|21	10|26	11|37	11|18	12|15
$W^- \rightarrow \ell ^-\bar{\nu }$	11	12|20	8.9|27	8.1|31	12|19	7.8|17
$Z/\gamma ^* \rightarrow \ell \ell$ $(m_{\ell \ell }=46$–$66\,\,\text {GeV}$)	6	17|21	11|30	18|24	21|22	28|36
$Z/\gamma ^* \rightarrow \ell \ell$ $(m_{\ell \ell }=66$–$116\,\,\text {GeV})$	12	24|51	16|66	20|116	14|109	18|26
Forward $Z/\gamma ^* \rightarrow \ell \ell$ $(m_{\ell \ell }=66$–$116\,\,\text {GeV})$	9	7.3|9.3	10|12	12|13	14|18	6.8|7.5
$Z/\gamma ^* \rightarrow \ell \ell$ $(m_{\ell \ell }=116$–$150\,\,\text {GeV})$	6	6.1|6.6	6.3|6.1	5.9|6.6	6.1|8.8	6.7|6.6
Forward $Z/\gamma ^* \rightarrow \ell \ell$ $(m_{\ell \ell }=116$–$150\,\,\text {GeV})$	6	4.2|3.9	5.1|4.3	5.6|4.6	5.1|5.0	3.6|3.5
Correlated $\chi ^2$		57|90	39|123	43|167	69|157	31|48
Total $\chi ^2$	61	136|222	103|290	118|396	147|351	113|159

**Fig. 25:**
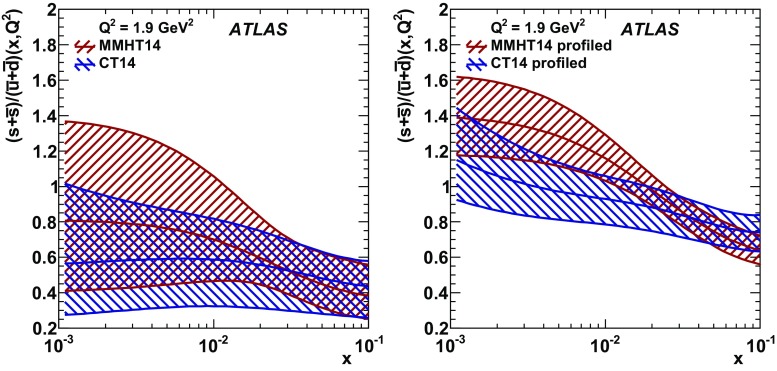
Ratio $R_s(x) = (s(x)+\bar{s}(x))/(\bar{u}(x)+\bar{d}(x))$ as a function of Bjorken-*x* at a scale of $Q^2=1.9\,\,\text {GeV}^2$ for the original MMHT14 and CT14 PDF sets (*left*) and for the MMHT14 and CT14 sets when profiled with the new *W*,  *Z* differential cross-section data (*right*)

Profiling PDFs, by introducing the data presented here, provides a shifted set of parton distributions with generally reduced uncertainties. Given the previous observation [38] of an enlarged strangeness fraction of the light sea, the effect of the data on the strange-quark distribution is examined. This is illustrated in Fig. 25, where the ratio $R_s(x)=(s(x)+\bar{s}(x))/(\bar{u}(x)+\bar{d}(x))$ is shown for two selected PDF sets, MMHT14 and CT14, before and after profiling, at a scale of $Q^2=1.9\,\,\text {GeV}^2$. The uncertainties of $R_s$ are seen to be significantly reduced and the central values, at $x \simeq 0.023$, increased towards unity, supporting the hypothesis of an unsuppressed strange-quark density at low *x*.

**Fig. 26:**
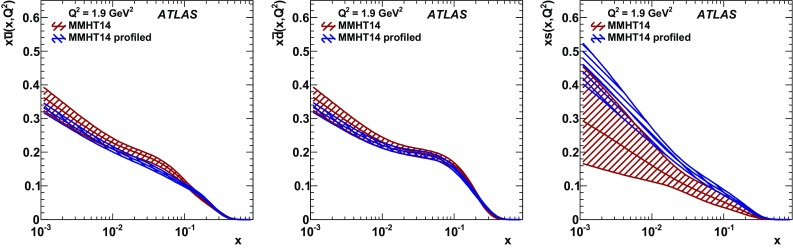
Distribution of $x\bar{u}$ (*left*), $x\bar{d}$ (*middle*) and *xs* (*right*) PDFs as a function of Bjorken-*x* at a scale of $Q^2=1.9\,\,\text {GeV}^2$ for the MMHT14 PDF set before and after profiling

**Fig. 27:**
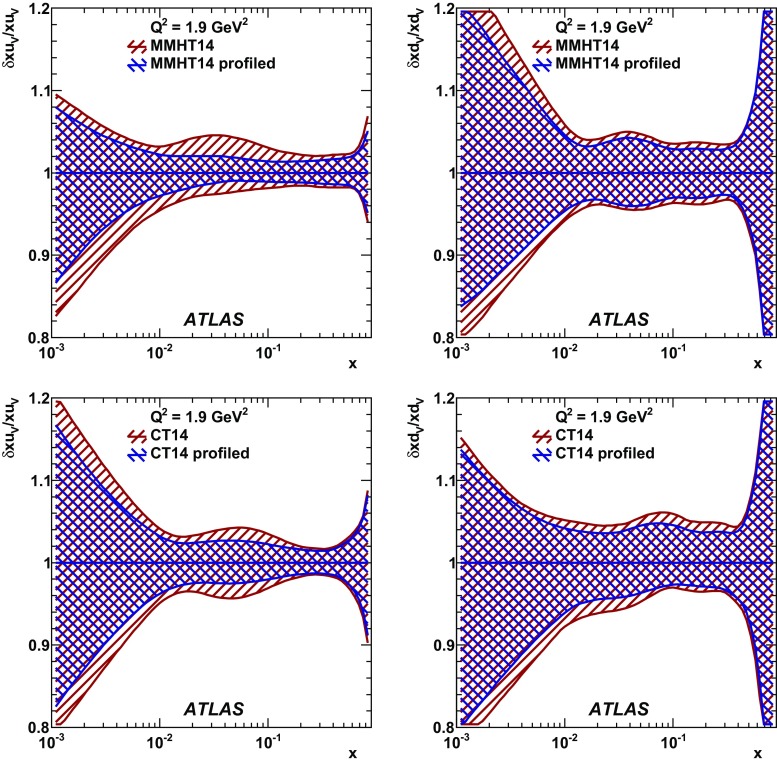
Effect of profiling on the relative uncertainties of the valence up-quark distribution $\delta xu_\mathrm {v}(x)/xu_\mathrm {v}(x)$ (*left*) and the valence down-quark distribution $\delta xd_\mathrm {v}(x)/xd_\mathrm {v}(x)$ (*right*) as a function of Bjorken-*x* at a scale of $Q^2=1.9\,\,\text {GeV}^2$. The *top row* shows the MMHT14 PDF set and the *bottom row* shows the CT14 PDF set

The sea-quark distributions, $x\bar{u}$, $x\bar{d}$ and $x\bar{s}$, before and after profiling with the MMHT14 set, are shown in Fig. 26. The strange-quark distribution is significantly increased and the uncertainties are reduced. This in turn leads to a significant reduction of the light sea, $x\bar{u}+x\bar{d}$, at low *x*, resulting from the tight constraint on the sum $4 \bar{u} + \bar{d} + \bar{s}$ from the precise measurement of the proton structure function $F_2$ at HERA. Some reduction of the uncertainty is also observed for the valence-quark distributions, $xu_\mathrm {v}$ and $xd_\mathrm {v}$, as is illustrated in Fig. 27 for the CT14 and MMHT14 sets.

## QCD analysis

In this section, the differential Drell–Yan production cross sections of $W^{\pm } \rightarrow \ell \nu$ and $Z/\gamma ^* \rightarrow \ell \ell$ $(\ell =e,\mu )$ are studied in combination with the final NC and CC deep inelastic scattering (DIS) HERA I+II data [32] within the framework of perturbative QCD. The Drell–Yan and DIS reactions are theoretically very well understood processes for such an analysis, and *ep* and *pp* collider data are particularly suitable because of the absence of nuclear corrections and negligible higher-twist effects. The HERA data alone can provide a full set of PDFs with certain assumptions [32]. Adding the ATLAS data provides more sensitivity to the flavour composition of the quark sea as well as to the valence-quark distributions at lower *x*. The HERA and ATLAS data are used to obtain a new set of PDFs, termed ATLAS-epWZ16. Following the previous, similar QCD fit analysis in Ref. [38], special attention is given to the evaluation of the strange-quark distribution, which was found to be larger than previous expectations based on dimuon data in DIS neutrino–nucleon scattering. The enhanced precision of the present data also permits a competitive determination of the magnitude of the CKM matrix element $\vert V_{cs} \vert$.

### Fit framework

The present QCD fit analysis is performed using the xFitter platform [113, 117] which uses QCDNUM [118] for PDF evolution and MINUIT [119] for minimization. Each step is cross-checked with an independent fit program as also used in Ref. [32].

Predictions for the differential CC and NC Drell–Yan cross sections calculated at fixed order in QCD at NNLO accuracy and with NLO electroweak corrections are described in Sect. 6.1. These calculations, however, cannot be used directly in an iterative fit because of the large computational effort required to produce even a single prediction. Therefore, the xFitter package uses the APPLGRID [120] code interfaced to the predictions of MCFM [121] for the fast calculation at fixed-order NLO accuracy in QCD. The improved NNLO QCD and NLO EW predictions discussed above are incorporated in the fit with additional $K$-factors defined as

20 $$\begin{aligned} K_\mathrm {f} = \frac{\sigma _\mathrm {NNLO\;QCD}^\mathrm {NLO\;EW}(\mathrm {DYNNLO})}{\sigma _\mathrm {NLO\;QCD}^\mathrm {LO\;EW} (\mathrm {APPLGRID})}. \end{aligned}$$

All predictions are calculated in the respective fiducial phase space of the experimental data. The $K$-factors are applied bin-by-bin and estimated using the same PDF, ATLAS-epWZ12, in both the numerator and denominator. They are typically close to unity within 1–2%, but are up to $6\%$ in the low-mass region, $m_{\ell \ell }= 46$–$66\,\text {GeV}$. These higher-order corrections are calculated using DYNNLO 1.5 and cross-checked with FEWZ3.1.b2 as detailed in Sect. 6.1. The $K$-factors are available as xFitter format files.

The QCD analysis uses the full set of ATLAS $W^{\pm } \rightarrow \ell \nu$ and $Z/\gamma ^* \rightarrow \ell \ell$ data, as described in the preceding sections, together with the combined H1 and ZEUS *ep* data [32]. There are 131 sources of experimental correlated systematic uncertainty for the ATLAS data and 167 sources of experimental correlated systematic uncertainty for the HERA data. The statistical precision of the $K$-factors  is typically ${<}0.1\%$ per measurement bin and is accounted for as an extra uncorrelated systematic uncertainty.

The nominal fit analysis is performed using the variable flavour number scheme from Refs [122, 123].^7^ The heavy-quark distributions are generated dynamically above the respective thresholds chosen as $m_c = 1.43\,\text {GeV}$ for the charm quark and as $m_b=4.5\,\text {GeV}$ for the bottom quark, corresponding to the recent heavy-quark differential cross-section measurements at HERA [135]. The PDFs are parameterized at the starting scale $Q_0^2=1.9\,\text {GeV}^2$, chosen to be below the charm-mass threshold as required by QCDNUM. The strong coupling constant at the *Z* mass is set to be $\alpha _{\text {S}} (m_Z)=0.118$, a value conventionally used by recent PDF analyses.

Besides the gluon distribution, *xg*, the valence and anti-quark distributions $xu_\mathrm {v}$, $xd_\mathrm {v}$, $x\bar{u}$, $x\bar{d}$, $x\bar{s}$, are parameterized at the starting scale $Q_0^2$, assuming that the sea quark and anti-quark distributions are the same. These distributions are evolved to the scale of the measurements and convolved with hard-scattering coefficients to obtain the theoretical cross-section predictions. The prediction is then confronted with the data through the $\chi ^2$ function,

21 $$\begin{aligned} \chi ^2(\mathbf {b}_{\mathrm {exp}})= & {} \nonumber \sum _{i=1}^{N_\mathrm {data}} \frac{\textstyle \left[ \sigma ^\mathrm {exp}_i - \sigma ^\mathrm {th}_i \left( 1 - \sum _j \gamma ^\mathrm {exp}_{ij} b_{j,\mathrm {\exp }}\right) \right] ^2}{\Delta _i^2}\nonumber \\&+\, \sum _{j=1}^{N_\mathrm {exp. sys.}} b_{j,\mathrm {exp}}^2 + \sum _{i=1}^{N_\mathrm {data}} \ln \frac{\Delta _i^2}{(\delta _{i,\mathrm {sta}}\sigma ^\mathrm {exp}_i)^2 + (\delta _{i,\mathrm {unc}}\sigma ^\mathrm {exp}_i)^2}, \end{aligned}$$

which is defined similarly to Eq. (16) and accounts for the various sources of correlated and uncorrelated uncertainties. The definition of $\Delta _i^2$ with scaled uncertainties is given by Eq. (17) and discussed there. This particular form is of higher importance in this context, as the relative uncertainties of the HERA data points can be large in parts of the phase space. The use of this form of $\Delta _i^2$ leads to a logarithmic term, introduced in Ref. [124], arising from the likelihood transition to $\chi ^2$. The contribution to the $\chi ^2$ from the last two sums related to the nuisance parameter constraints and the logarithmic term is referred to as “correlated + log penalty” later.

The optimal functional form for the parameterization of each parton distribution is found through a parameter scan requiring $\chi ^2$ saturation [125, 126]. The general form is of the type $A_i x^{B_i}(1-x)^{C_i} P_i(x)$ for each parton flavour *i*. The scan starts with the contribution of the factors $P_i(x)=(1+D_i x+E_ix^2)e^{F_i x}$ set to unity by fixing the parameters $D_i=E_i=F_i=0$ for all parton flavours. The parameter $A_g$ is constrained by the momentum sum rule relating the sum of the quark and gluon momentum distribution integrals, while the parameters $A_{u_\mathrm {v}}$ and $A_{d_\mathrm {v}}$ are fixed by the up and down valence-quark number sum rules. The assumption that $\bar{u}=\bar{d}$ as $x\rightarrow 0$ implies that $A_{\bar{u}} = A_{\bar{d}}$ and $B_{\bar{u}} = B_{\bar{d}}$. The procedure thus starts with ten free parameters and, subsequently, additional parameters are introduced one at a time.^8^ A parameterization with 15 variables is found to be sufficient to saturate the $\chi ^2$ value after minimization, i.e. no further significant $\chi ^2$ reduction is observed when adding further parameters. The final parameterization used to describe the parton distributions at $Q^2=Q_0^2$ is:

22 $$\begin{aligned} x u_\mathrm {v}(x)= & {} A_{u_\mathrm {v}} x^{B_{u_\mathrm {v}}} (1-x)^{C_{u_\mathrm {v}}} ( 1 + E_{u_\mathrm {v}} x^2),\nonumber \\ x d_\mathrm {v}(x)= & {} A_{d_\mathrm {v}} x^{B_{d_\mathrm {v}}} (1-x)^{C_{d_\mathrm {v}}},\nonumber \\ x \bar{u} (x)= & {} A_{\bar{u}} x^{B_{\bar{u}}} (1-x)^{C_{\bar{u}}}, \nonumber \\ x \bar{d} (x)= & {} A_{\bar{d}} x^{B_{\bar{d}}} (1-x)^{C_{\bar{d}}}, \nonumber \\ x g(x)= & {} A_g x^{B_g} (1-x)^{C_g} - A'_gx^{B'_g}(1-x)^{C'_g}, \nonumber \\ x \bar{s}(x)= & {} A_{\bar{s}} x^{B_{\bar{s}}} (1-x)^{C_{\bar{s}}}, \end{aligned}$$

where $A_{\bar{u}}=A_{\bar{d}}$ and $B_{\bar{s}}=B_{\bar{d}}=B_{\bar{u}}$. Given the enhanced sensitivity to the strange-quark distribution through the ATLAS data, $A_{\bar{s}}$ and $C_{\bar{s}}$ appear as free parameters, assuming $s = \bar{s}$. The experimental data uncertainties are propagated to the extracted QCD fit parameters using the asymmetric Hessian method based on the iterative procedure of Ref. [127], which provides an estimate of the corresponding PDF uncertainties.

### Fit results

The $\chi ^{2}$ values characterizing the NNLO QCD fit to the ATLAS Drell–Yan and HERA DIS data are listed in Table 18. The fit describes both the HERA and the ATLAS data well. Most of the correlated systematic uncertainties are shifted by less than one standard deviation and none are shifted by more than twice their original size in the fit. The overall normalization is shifted by less than half of the luminosity uncertainty of $1.8\%$. The only significant departure from a partial ${\chi ^2/\mathrm {n.d.f.}}\sim 1$ is seen for the low-mass $Z/\gamma ^* \rightarrow \ell \ell$ data. Here the $K$-factors are large, and the theoretical uncertainties, such as the FEWZ-DYNNLO difference, are sizable. As described below, this part of the data has little influence on the extracted PDFs.

**Table 18:** Quality of the QCD fit, expressed as the $\chi ^2/\mathrm {n.d.f.}$, to the final DIS HERA data and the ATLAS differential $W \rightarrow \ell \nu$ and $Z/\gamma ^* \rightarrow \ell \ell$ cross-section measurements. This NNLO fit is the base for the new ATLAS-epWZ16 set of PDFs

Data set	ATLAS-epWZ16
	$$\chi ^2/\mathrm {n.d.f.}$$
ATLAS $W^+ \rightarrow \ell ^+\nu$	8.4/11
ATLAS $W^- \rightarrow \ell ^-\bar{\nu }$	12.3/11
ATLAS $Z/\gamma ^* \rightarrow \ell \ell$ $(m_{\ell \ell }=46$–$66\,\,\text {GeV})$	25.9/6
ATLAS $Z/\gamma ^* \rightarrow \ell \ell$ $(m_{\ell \ell }=66$–$116\,\,\text {GeV})$	15.8/12
ATLAS forward $Z/\gamma ^* \rightarrow \ell \ell$ $(m_{\ell \ell }=66$–$116\,\,\text {GeV})$	7.4/9
ATLAS $Z/\gamma ^* \rightarrow \ell \ell$ $(m_{\ell \ell }=116$–$150\,\,\text {GeV})$	7.1/6
ATLAS forward $Z/\gamma ^* \rightarrow \ell \ell$ $(m_{\ell \ell }=116$–$150\,\,\text {GeV})$	4.0/6
ATLAS correlated $+$ log penalty	27.2
ATLAS total	108/61
HERA I $+$ II CC $e^+p$	44.3/39
HERA I $+$ II CC $e^-p$	62.7/42
HERA I $+$ II NC $e^-p$	222/159
HERA I $+$ II NC $e^+p$	838/816
HERA correlated $+$ log penalty	45.5
HERA total	1213/1056
Total	1321/1102

**Fig. 28:**
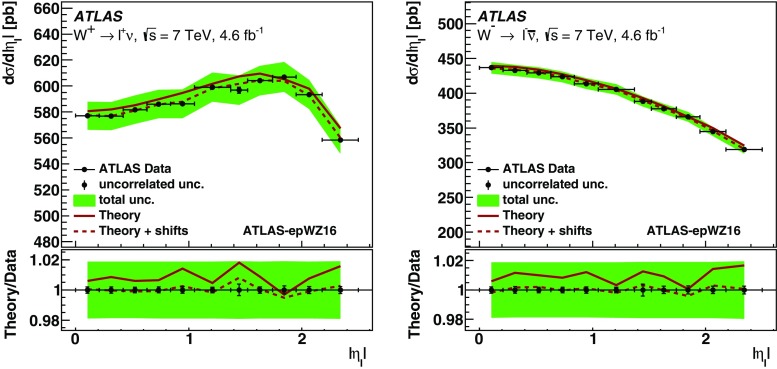
Differential cross-section measurements for $W^+ \rightarrow \ell ^+\nu$ (*right*) and $W^- \rightarrow \ell ^-\bar{\nu }$ (*left*) compared to the predictions of the QCD fit. The predictions are shown before (*solid lines*) and after (*dashed lines*) the shifts due to the correlated uncertainties are applied. The *lower box of each plot* shows the ratio of the theoretical calculations to the data

**Fig. 29:**
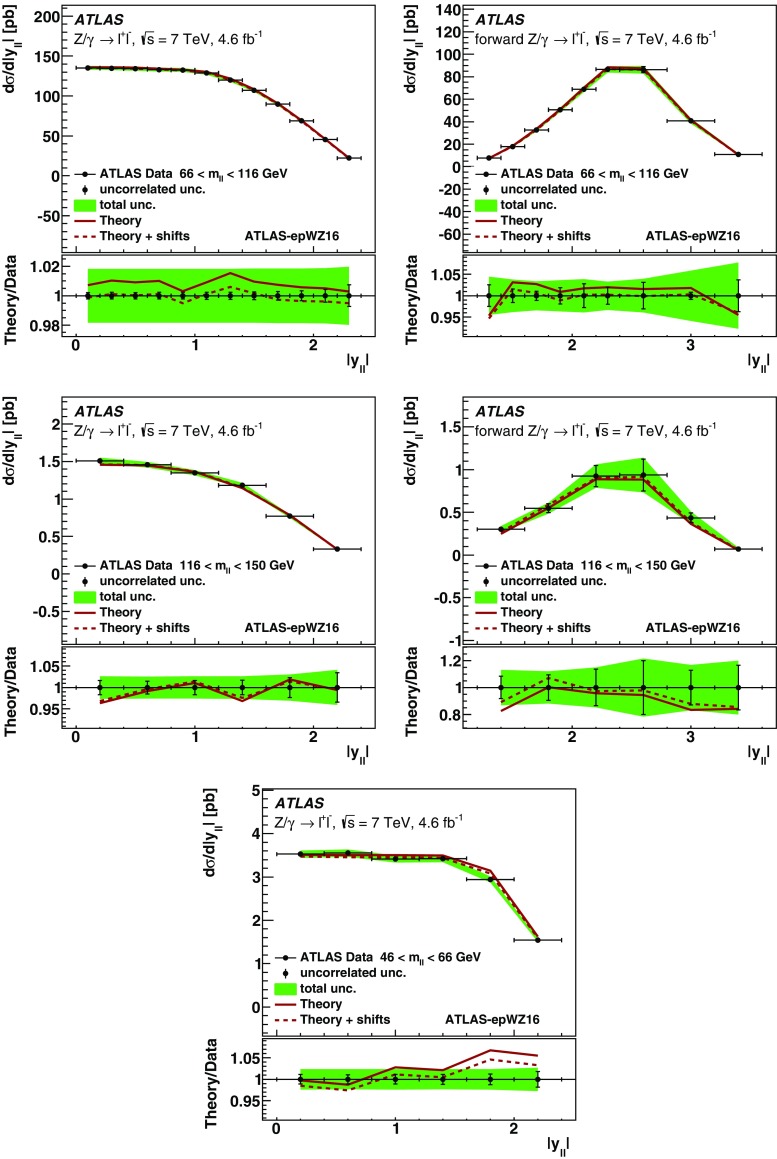
Differential $\mathrm {d}\sigma /\mathrm {d}|y_{\ell \ell }|$ cross-section measurement for $Z/\gamma ^* \rightarrow \ell \ell$ in the *Z*-peak region (*upper row*), as well as high dilepton mass $m_{\ell \ell }=116$–$150\,\,\text {GeV}$ (*middle row*), and low dilepton mass $m_{\ell \ell }=46$–$66\,\,\text {GeV}$ (*lower row*) compared to the QCD fit result. In the *Z*-peak region and at high dilepton mass the measurements are shown separately for both the *central* (*left*) and *forward* (*right*) regions. The predictions are shown before (*solid lines*) and after (*dashed lines*) the shifts due to the correlated uncertainties are applied. The *lower box of each plot* shows the ratio of the theoretical calculations to the data

Figure 28 shows the $W^+ \rightarrow \ell ^+\nu$ and $W^- \rightarrow \ell ^-\bar{\nu }$ lepton pseudorapidity distributions, which are well described by the fit. The fit results are presented before (solid) and after (dashed) application of the shifts accounting for the correlated systematic uncertainties of the data. Figure 29 presents the new ATLAS $Z/\gamma ^* \rightarrow \ell \ell$ measurements in the three different mass bins, further subdivided into the central and forward measurements. Also these data are well described by the QCD fit.

#### Parton distributions

**Fig. 30:**
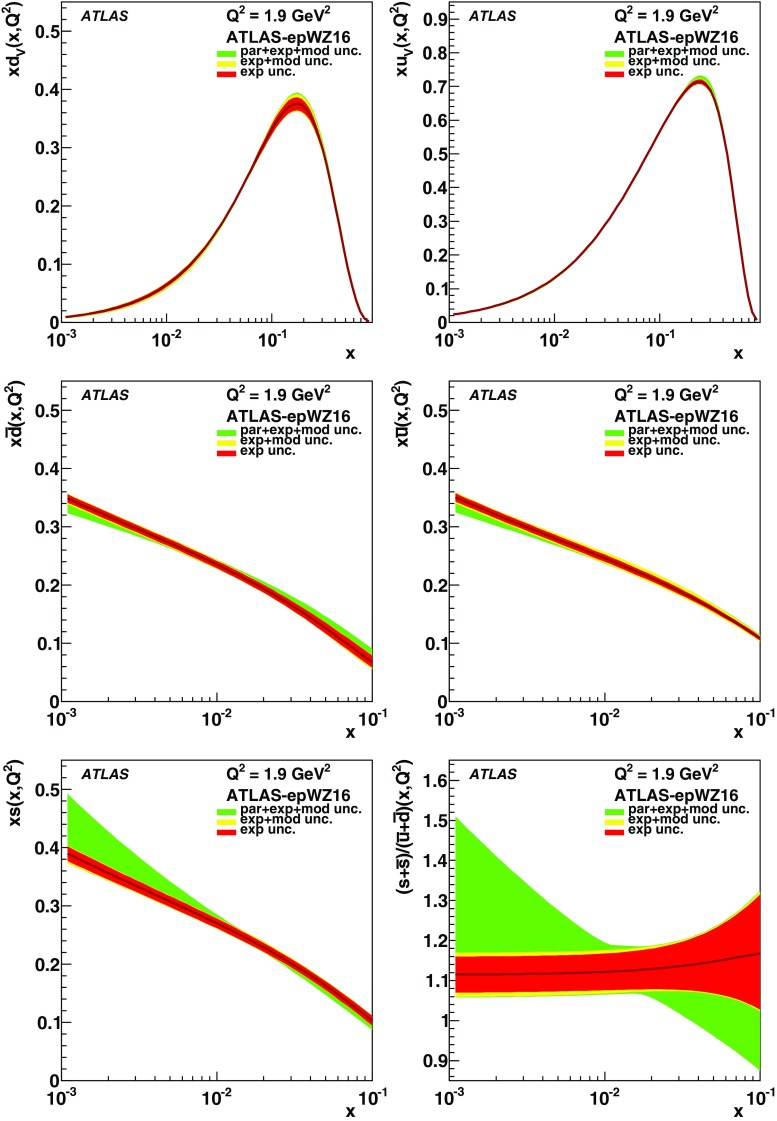
PDFs from the present ATLAS-epWZ16 determination at the starting scale $Q_0^2=1.9\,\text {GeV}^2$. *Top* valence PDFs $xd_\mathrm {v}(x)$, $xu_\mathrm {v}(x)$; *middle* light sea PDFs $x\bar{d}(x)$, $x\bar{u}(x)$; *bottom* strange-quark distribution and ratio $R_s(x)$. Uncertainty bands represent the experimental (*exp*), model (*mod*) and parameterization (*par*) components in *red*, *yellow* and *green*, respectively. The PDFs are shown in the region of maximum sensitivity of the ATLAS *W* and $Z/\gamma ^*$ data, $10^{-3}< x < 10^{-1}$, except for the valence quarks

The QCD fit determines a new set of PDFs, termed ATLAS-epWZ16, which has much smaller experimental uncertainties than the previous ATLAS-epWZ12 set. Further uncertainties in the PDFs are estimated and classified as model uncertainties and parameterization uncertainties, which are listed separately in Table 19. Model uncertainties comprise variations of $m_c$ and $m_b$ and variations of the starting scale value $Q_0^2$ and of the minimum $Q^2$ value ($Q^2_\mathrm {min}$) of the HERA data included in the analysis. The variation of the heavy-quark masses follows the HERAPDF2.0 analysis [32]. The variation of the charm-quark mass and the starting scale are performed simultaneously, as the constraint $Q_0^2<m_c^2$ has to be fulfilled. The parameterization uncertainties are estimated by adding further parameters in the polynomials $P_i(x)$ and allowing $B_{\bar{s}} \ne B_{\bar{d}}$. The PDFs including all uncertainties are shown in Fig. 30. The high level of precision of the data makes it necessary to evaluate further uncertainties, such as those from the effect of the renormalization and factorization scales and the limitations of the NNLO calculations. These are detailed below in terms of their influence on the ratio of strange quarks to the light sea.

**Table 19:** Overview of the impact of variations in the QCD fit regarding the model, parameterization, and further theoretical choices as compared to the nominal fit. For each variation the total fit $\chi ^2/\mathrm {n.d.f.}$ is given as well as the values of the two quantities $r_s$ and $R_s$ which describe the strange-to-light-sea-quark fraction at $Q_0^2$ and $x=0.023$. In the part of the table corresponding to the parameterization variations, the name of the additional parameter considered in addition to the 15-parameter set given in Eq. (22) is listed

Variation	Total $\chi ^2/\mathrm {n.d.f.}$	$r_s=\frac{s+\bar{s}}{2\bar{d}}$	$R_s=\frac{{s}+\bar{s}}{\bar{u}+\bar{d}}$
Nominal fit	1321/1102	1.193	1.131
Model variations			
$m_b=4.25\,\text {GeV}$	1319/1102	1.172	1.111
$m_b=4.75\,\text {GeV}$	1322/1102	1.211	1.149
$Q^2_\mathrm {min} = 5\,\text {GeV}^2$	1389/1149	1.202	1.128
$Q^2_\mathrm {min} = 10\,\text {GeV}^2$	1263/1062	1.188	1.129
$Q^2_0=1.6\,\text {GeV}^2$ and $m_c=1.37\,\text {GeV}$	1322/1101	1.198	1.148
$Q^2_0=2.2\,\text {GeV}^2$ and $m_c=1.49\,\text {GeV}$	1323/1101	1.197	1.119
Parameterization variations			
$B_{\bar{s}}$	1319/1101	1.094	1.067
$D_{\bar{s}}$	1321/1101	1.192	1.130
$D_{\bar{u}}$	1318/1101	1.184	1.128
$D_{\bar{d}}$	1321/1101	1.194	1.132
$D_{d_\mathrm {v}}$	1320/1101	1.195	1.132
$D_{u_\mathrm {v}}$	1320/1101	1.161	1.107
$D_{g}$	1319/1101	1.209	1.141
$F_{u_\mathrm {v}}$	1321/1101	1.206	1.143
$F_{d_\mathrm {v}}$	1323/1101	1.203	1.141
Theoretical uncertainties			
$\alpha _{\text {S}} (m_Z)=0.116$	1320/1102	1.185	1.121
$\alpha _{\text {S}} (m_Z)=0.120$	1323/1102	1.194	1.136
NLO EW down	1323/1102	1.199	1.132
NLO EW up	1319/1102	1.187	1.130
FEWZ 3.1b2	1314/1102	1.294	1.211

#### Strange-quark density

The QCD analysis of the ATLAS 2010 *W* and *Z* measurements [38] led to the unexpected observation that strangeness is unsuppressed at low *x* of ${\simeq } 0.023$ and low $Q^2=1.9\,\,\text {GeV}^2$, which means that the strange, down and up sea quarks are of similar strength in that kinematic range. This was supported by the ATLAS measurement of associated *W* and charm production [128] and not in contradiction with a similar measurement performed by CMS [20, 129]. But a large strange-quark density had not been expected from previous analyses of dimuon production in neutrino scattering [130–133] within the global PDF fit approaches  [31, 34, 35, 134].

The fraction of the strange-quark density in the proton can be characterized by a quantity $r_s$, defined as the ratio of the strange to the down sea-quark distributions. When evaluated at the scale $Q^2=Q^2_0=1.9\,\,\text {GeV}^2$ and $x=0.023$,^9^ the result is

23 $$\begin{aligned} r_s = \frac{s+\bar{s}}{2 \bar{d}}= 1.19 \pm 0.07\,\mathrm {(exp)}\;\pm 0.02\,\mathrm {(mod)}\;^{+0.02}_{-0.10}\,\mathrm {(par)}. \end{aligned}$$

Here the uncertainties relate to those of the experimental data (exp) determined by the Hessian method. The model (mod) and parameterization (par) uncertainties are discussed in Sect. 7.2.1 and the corresponding individual variations of $r_s$ are listed separately in Table 19. This result represents an improvement of a factor of three in the experimental uncertainty relative to the ATLAS-epWZ12 fit [38]. The improvement derives from the more precise ATLAS data, which provide the sensitivity to the strange-quark density through the shape of the *Z* rapidity distribution in combination with the common, absolute normalization of both the $W^\pm$ and $Z/\gamma ^*$ cross sections. The model uncertainties are reduced by a factor of three, mainly because of the better control of the charm-quark mass parameter from the HERA data [135]. The parameterization uncertainty is determined to be $^{+0.02}_{-0.10}$ as compared to $^{+0.10}_{-0.15}$ in the former analysis since the new, more precise data leave less freedom in the parameter choice. The variation to lower $r_s$ is dominated by the variation due to adding the $B_{\bar{s}}$ parameter which was not accounted for in the previous analysis. The result is thus a confirmation and improvement of the previous observation [38] of an unsuppressed strange-quark density in the proton. As a cross-check, a re-analysis of the 2010 data with the present theoretical framework was performed, which yields a value of $r_s$ consistent with both the former and the new value.

One may also express the strange-quark fraction with respect to the total light-quark sea, which is the sum of up and down sea-quark distributions, at the scale $Q^2=Q^2_0=1.9\,\,\text {GeV}^2$ and $x=0.023$:

24 $$\begin{aligned} R_s = \frac{s+\bar{s}}{\bar{u}+ \bar{d}}=1.13 \pm 0.05\,\mathrm {(exp)} \pm 0.02\,\mathrm {(mod)} \;^{+0.01}_{-0.06}\,\mathrm {(par)}. \end{aligned}$$

**Fig. 31:**
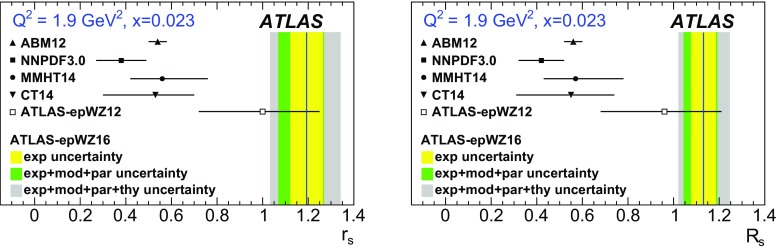
Determination of the relative strange-to-down sea quark fractions $r_s$ (*left*) and $R_s$ (*right*). *Bands* show the present result and its uncertainty contributions from experimental data, QCD fit, and theoretical uncertainties, see text; *closed symbols with horizontal error bars* give the predictions from different NNLO PDF sets; *open square* show the previous ATLAS result [38]. The ratios are calculated at the initial scale $Q_0^2=1.9\,\,\text {GeV}^2$ and at $x=0.023$ corresponding to the point of largest sensitivity at central rapidity of the ATLAS data

The new determinations of $r_s$ and $R_s$ are illustrated in Fig. 31. The measurement is presented with the experimental and the PDF-fit related uncertainties, where the latter results from adding the model and parameterization uncertainties in quadrature. The outer band illustrates additional, mostly theoretical uncertainties which are presented below. The result is compared with recent global fit analyses, ABM12, MMHT14, CT14 and NNPDF3.0. All of these predict $r_s$ and $R_s$ to be significantly lower than unity, with values between about 0.4 and 0.6. Furthermore, these global fit analyses are seen to exhibit substantially different uncertainties in $r_s$ and $R_s$ due to exploiting different data and prescriptions for fit uncertainties. The new result is in agreement with the previous ATLAS-epWZ12 analysis also shown in Fig. 31. It is also consistent with an earlier analysis by the NNPDF group [63] based on collider data only, which obtains a value near unity, albeit with large uncertainties.^10^

A careful evaluation of the value of $r_s$ requires the consideration of a number of additional, mostly theoretical uncertainties. These lead to the more complete result for $r_s$

25 $$\begin{aligned} r_s=1.19 \pm 0.07\,\mathrm {(exp)}\;^{+0.13}_{-0.14}\,\mathrm {(mod+par+thy)}. \end{aligned}$$

Here the previously discussed model and parameterization uncertainties are summarized and added together with further theoretical uncertainties (thy) as follows: (1) the uncertainty in $\alpha _{\text {S}} (m_Z^2)$ is taken to be $\pm 0.002$ with a very small effect on $r_s$; (2) the electroweak corrections and their application, as described in Sect. 6.1, introduce a one percent additional error for $r_s$; (3) the whole analysis was repeated with predictions obtained with the FEWZ program (version 3.1b2) leading to a value of $r_s$ enlarged by $+0.10$ as compared to the DYNNLO result; (4) finally the variation of the renormalization ($\mu _\mathrm {r}$) and factorization ($\mu _\mathrm {f}$) scales changes the result by $10\%$ if one varies these by factors of 2 up and 1 / 2 down (see below for further details). Table 20 details all uncertainty components of $r_s$ and also $R_s$.

**Table 20:** Summary of the central value and all uncertainties in the variables $r_s$ and $R_s$ evaluated at $Q^2=1.9\,\,\text {GeV}^2$ and $x=0.023$ characterizing the fraction of the strange-quark density in the proton

	$r_s=\frac{s+\bar{s}}{2\bar{d}}$	$R_s=\frac{s+\bar{s}}{\bar{u}+\bar{d}}$
Central value	1.19	1.13
Experimental data	$\pm 0.07$	$\pm 0.05$
Model ($m_b$, $Q^2_\mathrm {min}$, $Q^2_0\,\mathrm{and}\,m_c$)	$\pm 0.02$	$\pm 0.02$
Parameterization	$^{+0.02}_{-0.10}$	$^{+0.01}_{-0.06}$
$\alpha _{\text {S}}$	$^{+0.00}_{-0.01}$	$\pm 0.01$
EW corrections	$\pm 0.01$	$\pm 0.00$
QCD scales	$^{+0.08}_{-0.10}$	$^{+0.06}_{-0.07}$
FEWZ 3.1b2	$+0.10$	$+0.08$
Total uncertainty	$^{+0.15}_{-0.16}$	$\pm 0.11$

Various further cross-checks are performed in order to assess the reliability of the strange-quark density measurement.

- To test the sensitivity to assumptions about the low-*x* behaviour of the light-quark sea, the constraint on $\bar{u}=\bar{d}$ as $x\rightarrow 0$ is removed by allowing $A_{\bar{d}}$ and $B_{\bar{d}}$ to vary independently from the respective $A_{\bar{u}}$ and $B_{\bar{u}}$. The resulting $\bar{u}$ is compatible with $\bar{d}$ within uncertainties of ${\simeq } 8\%$ at $x \sim 0.001$ and $Q^2_0$, while $s+\bar{s}$ is found to be unsuppressed with $r_s = 1.16$.
- The ATLAS-epWZ16 PDF set results in a slightly negative central value of $x\bar{d}-x\bar{u}$ at $x\sim 0.1$, which with large uncertainties is compatible with zero. This result is about two standard deviations below the determination from E866 fixed-target Drell–Yan data [136] according to which $x\bar{d}-x\bar{u} \sim 0.04$ at $x\sim 0.1$. It has been suggested that the ATLAS parameterization forces a too small $x\bar{d}$ distribution if the strange-quark PDF is unsuppressed [134]. However, the E866 observation is made at $x \sim 0.1$, while the ATLAS *W*,  *Z* data have the largest constraining power at $x\sim 0.023$. For a cross-check, the E866 cross-section data was added to the QCD fit with predictions computed at NLO QCD. In this fit $x\bar{d}-x\bar{u}$ is enhanced and nevertheless the strange-quark distribution is found to be unsuppressed with $r_s$ near unity.
- Separate analyses of the electron and muon data give results about one standard deviation above and below the result using their combination. If the $W^{\pm }$ and *Z*-peak data are used without the $Z/\gamma ^*$ data at lower and higher $m_{\ell \ell }$, a value of $r_s=1.23$ is found with a relative experimental uncertainty almost the same as in the nominal fit.
- A suppressed strange-quark PDF may be enforced by fixing $r_s = 0.5$ and setting $C_{\bar{s}} = C_{\bar{d}}$. The total $\chi ^2$ obtained this way is 1503, which is 182 units higher than the fit allowing these two parameters to be free. The ATLAS partial $\chi ^2$ increases from 108 to 226 units for the 61 degrees of freedom. A particularly large increase is observed for the *Z*-peak data, where ${\chi ^2/\mathrm {n.d.f.}}= 53/12$ is found for a fit with suppressed strangeness.

A final estimate of uncertainties is performed with regard to choosing the renormalization and factorization scales in the calculation of the Drell–Yan cross sections. The central fit is performed using the dilepton and *W* masses, $m_{\ell \ell }$ and $m_W$, as default scale choices. Conventionally both scales are varied by a factor of 2 and 0.5 as an estimate of missing higher-order QCD terms. Table 21 presents the results of varying the scales separately and jointly. It is observed that a choice of half the mass values leads to a significant improvement of the $\chi ^2$ by about 24 units. All separate variations of $\mu _\mathrm {r}$ and $\mu _\mathrm {f}$ cause the resulting strange fraction values to be inside the envelope obtained from the joint variation $\mu _\mathrm {r}=\mu _\mathrm {f}$ up or down.

**Table 21:** Effect of varying the scales for the Drell–Yan data in the NNLO QCD fit. The renormalization, $\mu _\mathrm {r}$, and factorization, $\mu _\mathrm {f}$, scales, are expressed relatively to the dilepton mass for NC and the *W* mass for the CC cross section. Changes of the total fit $\chi ^2$ values are almost exclusively due to variations of the ATLAS values while the HERA $\chi ^2$, given by their difference, remains nearly constant. Right columns: resulting $r_s$ and $R_s$ values, quoted at $Q^2=Q_0^2$ and $x=0.023$

$\mu _\mathrm {r}$	$\mu _\mathrm {f}$	$\chi ^2/\mathrm {n.d.f.}$		$r_s=\frac{s+\bar{s}}{2\bar{d}}$	$R_s=\frac{s+\bar{s}}{\bar{u}+\bar{d}}$
		Total	ATLAS		
1	1	1321/1102	108/61	1.193	1.131
1/2	1/2	1297/1102	85/61	1.093	1.066
2	2	1329/1102	115/61	1.270	1.186
1	1/2	1307/1102	94/61	1.166	1.115
1	2	1312/1102	100/61	1.201	1.130
1/2	1	1304/1102	94/61	1.128	1.088
2	1	1321/1102	107/61	1.241	1.165

#### Determination of $\vert V_{cs} \vert$

As discussed in the preceding section, the combination of HERA DIS and newly presented ATLAS measurements results in a precise determination of the light-quark composition of the proton and specifically of the strange-quark density. The most significant contributions to *W*-boson production are from the Cabibbo-favoured initial states *ud* and *cs*, where the rate is also controlled by the magnitude of the CKM matrix elements $|V_{ud}|$ and $\vert V_{cs} \vert$. While $|V_{ud}|$ is experimentally measured to very high precision, this is not true for the $\vert V_{cs} \vert$ element. The contributions from the Cabibbo-suppressed initial state *cd*, which are sensitive to $|V_{cd}|$, are suppressed by one order of magnitude compared to the *cs* contribution. Both the $W^\pm$ production rates and the lepton pseudorapidity distributions contain information about the $cs\rightarrow W$ contribution to the CC Drell–Yan cross section. A PDF fit as described above is performed, but in addition the $\vert V_{cs} \vert$ parameter is allowed to vary freely while all other CKM matrix elements are fixed to the values given in Table 15, which were obtained from a global fit imposing unitarity. The following value and corresponding uncertainties are found

26 $$\begin{aligned}\vert V_{cs} \vert &=  0.969 \pm0.013\,\mathrm{(exp)}^{+0.006}_{-0.003}\,\mathrm{(mod)}^{+0.003}_{-0.027}  \mathrm{(par)}\\&\quad\,^{+0.011}_{-0.005}\,\mathrm{(thy)}.\end{aligned}$$

Table 22 details all the uncertainty components of $\vert V_{cs} \vert$. In this fit the value of $r_s$ is found to be 1.18, compared to 1.19 when $\vert V_{cs} \vert$ is fixed to the value assuming unitarity of the CKM matrix. The experimental uncertainty of $\vert V_{cs} \vert$ is $66\%$ correlated with the parameter $A_s$ controlling the normalization of the strange-quark density, while the parameter $B_s$ is fixed to $B_{\bar{d}}$. The correlation with $C_s$ is found to be $10\%$.

**Table 22:** Summary of the central value and all uncertainties in the CKM matrix element $\vert V_{cs} \vert$

	$$\vert V_{cs} \vert $$
Central value	0.969
Experimental data	$\pm 0.013$
Model ($m_b$, $Q^2_\mathrm {min}$, $Q^2_0\,\mathrm{and}\,m_c$)	$^{+0.006}_{-0.003}$
Parameterization	$^{+0.003}_{-0.027}$
$\alpha _{\text {S}}$	$\pm 0.000$
EW corrections	$\pm 0.004$
QCD scales	$^{+0.000}_{-0.003}$
FEWZ 3.1b2	$+0.011$
Total uncertainty	$^{+0.018}_{-0.031}$

The dominant uncertainty of $\vert V_{cs} \vert$ arises from the parameterization variation associated with the extra freedom given to the strange-quark distribution by releasing the assumption $B_{\bar{d}}=B_{\bar{s}}$ that fixes the rise of $x\bar{d}(x)$ and $x\bar{s}(x)$ to be the same at low *x*.

**Fig. 32:**
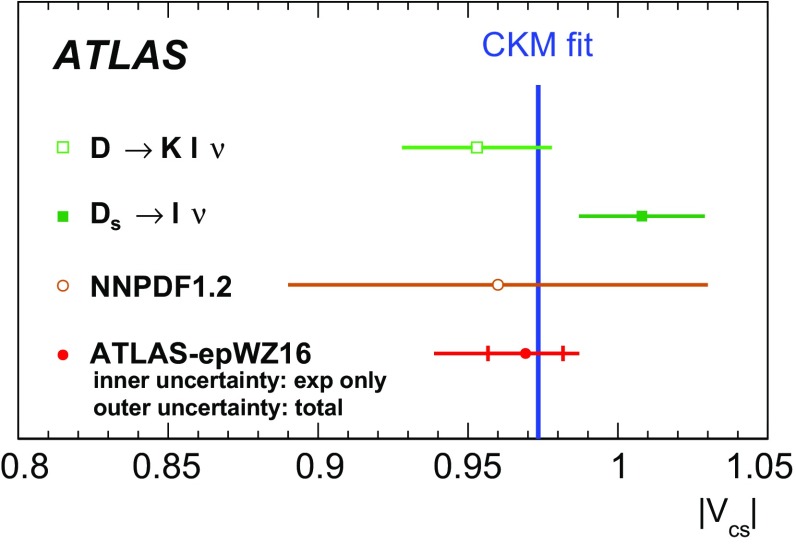
$\vert V_{cs} \vert$ as determined in the global CKM fit cited by the PDG [39] (*blue vertical line*) compared to extractions from $D_s \rightarrow \ell \nu$ and $D \rightarrow K\ell \nu$ decays [39] and the NNPDF1.2 fit [146]. The ATLAS-epWZ16 fit result is shown with uncertainty contributions from the experimental data (*inner error bar*) and the total uncertainty including all fit and further theoretical uncertainties (*outer error bar*). The uncertainty in $\vert V_{cs} \vert$ from the CKM fit with unitarity constraint is smaller than the width of the *vertical line*

This determination represents a new, competitive measurement of $\vert V_{cs} \vert$. Figure 32 compares the result to determinations of $\vert V_{cs} \vert$ extracted from leptonic $D_s$ meson decays, $D_s \rightarrow \ell \nu$ [137–142], and from semileptonic *D* meson decays, $D \rightarrow K\ell \nu$ [142–145], from data by the CLEO-c, BABAR, and Belle experiments as reported in Ref. [39]. In addition, an early determination of $\vert V_{cs} \vert$ by the NNPDF Collaboration from a QCD fit is shown [146].

## Summary

New cross-section measurements by the ATLAS Collaboration are presented for inclusive Drell–Yan production in the neutral-current channel, $Z/\gamma ^* \rightarrow \ell \ell$, and the charged-current channel, $W^+ \rightarrow \ell ^+\nu$ and $W^- \rightarrow \ell ^-\bar{\nu }$. The measurement is based on data taken in *pp* collisions at the LHC at a centre-of-mass energy of $\sqrt{s}=7\,\text {TeV}$ with an integrated luminosity of $4.6\,\mathrm {fb}^{-1}$. Cross sections are provided in the electron and muon decay channels, integrated over the fiducial regions and differentially. The $W^+ \rightarrow \ell ^+\nu$ and $W^- \rightarrow \ell ^-\bar{\nu }$ cross sections are measured as a function of lepton pseudorapidity $\eta _{\ell }$. The $Z/\gamma ^* \rightarrow \ell \ell$ cross sections are measured as a function of the dilepton rapidity, $y_{\ell \ell }$, in three dilepton mass bins $46< m_{\ell \ell }<150\,\text {GeV}$ in the central region and extended into the forward region up to $|y_{\ell \ell }|=3.6$ for $66< m_{\ell \ell }<150\,\text {GeV}$.

The electron and muon channel results are combined considering all sources of correlated and uncorrelated uncertainties. A new sensitive test of electron–muon universality in on-shell *W* and *Z* decays is presented. The combined integrated fiducial $W^+,~W^-,$ and *Z* cross sections are measured to an experimental precision of 0.6,  0.5,  and $0.32\%$, respectively, apart from the common $1.8\%$ normalization uncertainty through the luminosity determination. The differential measurements are nearly as precise as the integrated cross-section results except at the edges of the phase space. With the full information about correlated uncertainties given, the data provide correspondingly precise results of cross-section ratios and the $W^{\pm }$ lepton charge asymmetry as well.

A measurement precision at sub-percent level represents an opportunity and challenge for the QCD interpretation. Predictions for the Drell–Yan processes $W^{\pm } \rightarrow \ell \nu$ and $Z/\gamma ^* \rightarrow \ell \ell$ are calculated at NNLO fixed order in QCD and including NLO electroweak corrections. A quantitative comparison of the differential cross sections shows deviations of the predictions obtained with many of the contemporary PDF sets, hinting to a special impact of the data on the determination of the strange-quark distribution.

An NNLO QCD analysis is performed on the new $W^{\pm } \rightarrow \ell \nu$ and $Z/\gamma ^* \rightarrow \ell \ell$ ATLAS data together with the final, combined data from H1 and ZEUS on inclusive neutral-current and charged-current deep inelastic scattering. A new set of parton distribution functions, termed ATLAS-epWZ16, is provided. A detailed fit analysis supports the previous observation by ATLAS of a large ratio of the strange-quark distribution to the lighter sea-quark distributions at low *x*. Specifically, the ratio of the strange to the down sea-quark distributions, evaluated at a scale of $Q^2=1.9\,\,\text {GeV}^2$ at a mean $x=0.023$, is found to be $r_s=1.19$ with a total uncertainty of 0.16. Experimentally, $r_s$ is determined with an uncertainty of 0.07 which is a threefold reduction relative to the previous determination by the ATLAS Collaboration.

A complete set of uncertainties in the QCD fit result is provided in addition to the experimental uncertainties. This covers the effects of model, parameterization, and further theoretical uncertainties. Detailed studies are performed regarding the accuracy with which NNLO QCD predictions for the Drell–Yan process can be computed, including the differences in existing codes, DYNNLO and FEWZ, and the effect of the choice of scales. The uncertainties in the strange-quark density from the limitations of NNLO QCD calculations of the fiducial cross sections are found to significantly exceed the experimental errors. An interesting observation is the significant improvement in the description of the ATLAS data when factorization and renormalization scales are set to a half of the canonically used dilepton mass scales. Several cross-checks are presented to evaluate the reliability of the measured enhancement of the strange-quark density. The paper finally presents a determination of the CKM matrix element $\vert V_{cs} \vert$ which has a precision comparable to extractions from charm meson decays.

## Appendix

### Differential measurements in electron and muon channels

The differential cross-sectionmeasurements for electron and muon channels before combination are shown in Tables 23, 24, 25, 26, 27, 28 and 29.

**Table 23:** Differential cross section for the $W^- \rightarrow e^- \bar{\nu }$ (a) and $W^+ \rightarrow e^+ \nu$ (b) processes, extrapolated to the common fiducial region. The relative statistical ($\delta _\mathrm {sta}$), uncorrelated systematic ($\delta _\mathrm {unc}$), correlated systematic ($\delta _\mathrm {sys}$), and total ($\delta _\mathrm {tot}$) uncertainties are given in percent. The overall $1.8$% luminosity uncertainty is not included

$|\eta _{\ell }|^\mathrm {min}$	$|\eta _{\ell }|^\mathrm {max}$	$\mathrm {d}\sigma /\mathrm {d}|\eta _{\ell }|$ (pb)	$\delta _\mathrm {sta}$ (%)	$\delta _\mathrm {unc}$ (%)	$\delta _\mathrm {sys}$ (%)	$\delta _\mathrm {tot}$ (%)
(a)						
0.00	0.21	436.8	0.15	0.15	0.91	0.93
0.21	0.42	433.1	0.14	0.17	0.89	0.91
0.42	0.63	430.0	0.14	0.15	0.90	0.92
0.63	0.84	424.5	0.14	0.13	0.99	1.01
0.84	1.05	415.3	0.15	0.17	1.08	1.10
1.05	1.37	405.1	0.13	0.16	1.36	1.38
1.52	1.74	371.0	0.17	0.17	1.31	1.34
1.74	1.95	367.6	0.18	0.26	1.26	1.30
1.95	2.18	345.8	0.17	0.18	1.28	1.31
2.18	2.50	322.3	0.2	0.2	2.2	2.2

$|\eta _{\ell }|^\mathrm {min}$	$|\eta _{\ell }|^\mathrm {max}$	$\mathrm {d}\sigma /\mathrm {d}|\eta _{\ell }|$ (pb)	$\delta _\mathrm {sta}$ (%)	$\delta _\mathrm {unc}$ (%)	$\delta _\mathrm {sys}$ (%)	$\delta _\mathrm {tot}$ (%)
(b)						
0.00	0.21	577.2	0.13	0.14	1.00	1.01
0.21	0.42	577.5	0.12	0.15	0.94	0.96
0.42	0.63	583.2	0.12	0.14	0.93	0.95
0.63	0.84	588.7	0.12	0.12	0.97	0.98
0.84	1.05	588.4	0.12	0.16	0.94	0.96
1.05	1.37	598.5	0.10	0.15	1.13	1.14
1.52	1.74	593.7	0.14	0.14	1.17	1.19
1.74	1.95	610.8	0.14	0.19	1.03	1.05
1.95	2.18	594.6	0.12	0.15	1.04	1.05
2.18	2.50	559.6	0.13	0.15	1.55	1.56

**Table 24:** Differential cross section for the $Z/\gamma ^* \rightarrow e^+e^-$ process in the central region with $46<m_{\ell \ell }<66\,\,\text {GeV}$, extrapolated to the common fiducial region. The relative statistical ($\delta _\mathrm {sta}$), uncorrelated systematic ($\delta _\mathrm {unc}$), correlated systematic ($\delta _\mathrm {sys}$), and total ($\delta _\mathrm {tot}$) uncertainties are given in percent. The overall $1.8$% luminosity uncertainty is not included

$|y_{\ell \ell }|^\mathrm {min}$	$|y_{\ell \ell }|^\mathrm {max}$	$\mathrm {d}\sigma /\mathrm {d}|y_{\ell \ell }|$ (pb)	$\delta _\mathrm {sta}$ (%)	$\delta _\mathrm {unc}$ (%)	$\delta _\mathrm {sys}$ (%)	$\delta _\mathrm {tot}$ (%)
0.00	0.40	3.595	1.5	0.9	1.3	2.2
0.40	0.80	3.622	1.5	0.8	1.2	2.1
0.80	1.20	3.456	1.8	0.9	1.4	2.4
1.20	1.60	3.382	2.0	1.0	1.5	2.7
1.60	2.00	2.968	2.3	1.1	1.5	2.9
2.00	2.40	1.567	2.9	1.2	1.2	3.4

**Table 25:** Differential cross section for the $Z/\gamma ^* \rightarrow e^+e^-$ process in the central (a) and forward (b) region with $66<m_{\ell \ell }<116\,\,\text {GeV}$, extrapolated to the common fiducial region. The relative statistical ($\delta _\mathrm {sta}$), uncorrelated systematic ($\delta _\mathrm {unc}$), correlated systematic ($\delta _\mathrm {sys}$), and total ($\delta _\mathrm {tot}$) uncertainties are given in percent. The overall $1.8$% luminosity uncertainty is not included

$|y_{\ell \ell }|^\mathrm {min}$	$|y_{\ell \ell }|^\mathrm {max}$	$\mathrm {d}\sigma /\mathrm {d}|y_{\ell \ell }|$ (pb)	$\delta _\mathrm {sta}$ (%)	$\delta _\mathrm {unc}$ (%)	$\delta _\mathrm {sys}$ (%)	$\delta _\mathrm {tot}$ (%)
(a)						
0.00	0.20	135.6	0.28	0.18	0.40	0.52
0.20	0.40	135.3	0.29	0.16	0.39	0.52
0.40	0.60	133.9	0.30	0.16	0.39	0.51
0.60	0.80	133.7	0.31	0.17	0.40	0.54
0.80	1.00	132.9	0.32	0.18	0.41	0.55
1.00	1.20	129.4	0.34	0.20	0.41	0.57
1.20	1.40	120.2	0.36	0.19	0.44	0.60
1.40	1.60	106.5	0.38	0.19	0.43	0.61
1.60	1.80	89.3	0.44	0.23	0.54	0.73
1.80	2.00	68.7	0.51	0.30	0.39	0.71
2.00	2.20	46.03	0.59	0.39	0.47	0.85
2.20	2.40	21.86	0.91	0.67	0.74	1.35
(b)						
1.20	1.40	7.71	1.8	1.8	3.2	4.1
1.40	1.60	17.95	1.0	1.1	3.0	3.4
1.60	1.80	32.57	0.7	0.7	2.7	2.9
1.80	2.00	50.5	0.6	1.8	2.6	3.2
2.00	2.20	68.5	0.6	2.7	2.2	3.5
2.20	2.40	86.6	0.5	1.9	1.9	2.8
2.40	2.80	86.1	0.3	3.0	1.7	3.5
2.80	3.20	40.71	0.5	0.6	5.5	5.6
3.20	3.60	11.00	1.2	3.7	6.4	7.5

**Table 26:** Differential cross section for the $Z/\gamma ^* \rightarrow e^+e^-$ process in the central (a) and forward (b) region with $116<m_{\ell \ell }<150\,\,\text {GeV}$, extrapolated to the common fiducial region. The relative statistical ($\delta _\mathrm {sta}$), uncorrelated systematic ($\delta _\mathrm {unc}$), correlated systematic ($\delta _\mathrm {sys}$), and total ($\delta _\mathrm {tot}$) uncertainties are given in percent. The overall $1.8$% luminosity uncertainty is not included

$|y_{\ell \ell }|^\mathrm {min}$	$|y_{\ell \ell }|^\mathrm {max}$	$\mathrm {d}\sigma /\mathrm {d}|y_{\ell \ell }|$ (pb)	$\delta _\mathrm {sta}$ (%)	$\delta _\mathrm {unc}$ (%)	$\delta _\mathrm {sys}$ (%)	$\delta _\mathrm {tot}$ (%)
(a)						
0.00	0.40	1.503	2.0	2.5	1.4	3.5
0.40	0.80	1.422	2.1	0.9	1.4	2.7
0.80	1.20	1.329	2.3	1.3	1.4	3.0
1.20	1.60	1.181	2.6	1.6	1.5	3.4
1.60	2.00	0.754	3.3	2.4	2.0	4.6
2.00	2.40	0.328	4.9	2.4	1.8	5.7
(b)						
1.20	1.60	0.300	6.8	6.6	9.1	13.1
1.60	2.00	0.547	5.2	7.8	7.3	11.9
2.00	2.40	0.912	4.0	13.5	4.5	14.8
2.40	2.80	0.931	3.9	20.9	4.0	21.6
2.80	3.20	0.438	5.3	14.4	6.8	16.8
3.20	3.60	0.070	14.5	11.6	7.2	19.9

**Table 27:** Differential cross section for the $W^- \rightarrow \mu ^- \bar{\nu }$ (a) and $W^+ \rightarrow \mu ^+ \nu$ (b) processes, extrapolated to the common fiducial region. The relative statistical ($\delta _\mathrm {sta}$), uncorrelated systematic ($\delta _\mathrm {unc}$), correlated systematic ($\delta _\mathrm {sys}$), and total ($\delta _\mathrm {tot}$) uncertainties are given in. The overall $1.8$% luminosity uncertainty is not included

$|\eta _{\ell }|^\mathrm {min}$	$|\eta _{\ell }|^\mathrm {max}$	$\mathrm {d}\sigma /\mathrm {d}|\eta _{\ell }|$ (pb)	$\delta _\mathrm {sta}$ (%)	$\delta _\mathrm {unc}$ (%)	$\delta _\mathrm {sys}$ (%)	$\delta _\mathrm {tot}$ (%)
(a)						
0.00	0.21	439.0	0.16	0.41	0.67	0.80
0.21	0.42	437.0	0.15	0.52	0.55	0.77
0.42	0.63	431.4	0.14	0.27	0.59	0.67
0.63	0.84	425.6	0.15	0.33	0.62	0.72
0.84	1.05	413.5	0.16	0.29	0.60	0.69
1.05	1.37	406.8	0.12	0.29	0.56	0.65
1.37	1.52	389.2	0.17	0.34	0.55	0.67
1.52	1.74	380.6	0.14	0.43	0.60	0.75
1.74	1.95	367.1	0.15	0.32	0.62	0.71
1.95	2.18	345.0	0.14	0.38	0.63	0.75
2.18	2.50	318.3	0.15	0.50	0.67	0.85
(b)						
0.00	0.21	581.3	0.14	0.41	0.63	0.77
0.21	0.42	583.6	0.13	0.46	0.58	0.75
0.42	0.63	583.2	0.12	0.25	0.57	0.64
0.63	0.84	587.3	0.13	0.31	0.59	0.67
0.84	1.05	585.6	0.14	0.37	0.59	0.71
1.05	1.37	601.5	0.10	0.26	0.59	0.65
1.37	1.52	599.1	0.13	0.33	0.57	0.67
1.52	1.74	607.5	0.11	0.31	0.57	0.66
1.74	1.95	604.4	0.11	0.50	0.57	0.76
1.95	2.18	598.7	0.10	0.57	0.60	0.83
2.18	2.50	563.1	0.11	0.60	0.63	0.88

**Table 28:** Differential cross section for the $Z/\gamma ^* \rightarrow \mu ^+\mu ^-$ process in the region with $66<m_{\ell \ell }<116\,\,\text {GeV}$, extrapolated to the common fiducial region. The relative statistical ($\delta _\mathrm {sta}$), uncorrelated systematic ($\delta _\mathrm {unc}$), correlated systematic ($\delta _\mathrm {sys}$), and total ($\delta _\mathrm {tot}$) uncertainties are given in percent. The overall $1.8$% luminosity uncertainty is not included

$|y_{\ell \ell }|^\mathrm {min}$	$|y_{\ell \ell }|^\mathrm {max}$	$\mathrm {d}\sigma /\mathrm {d}|y_{\ell \ell }|$ (pb)	$\delta _\mathrm {sta}$ (%)	$\delta _\mathrm {unc}$ (%)	$\delta _\mathrm {sys}$ (%)	$\delta _\mathrm {tot}$ (%)
0.00	0.20	134.8	0.25	0.12	0.41	0.50
0.20	0.40	134.2	0.26	0.12	0.41	0.50
0.40	0.60	134.3	0.26	0.11	0.41	0.50
0.60	0.80	132.5	0.26	0.11	0.41	0.50
0.80	1.00	132.2	0.25	0.12	0.40	0.48
1.00	1.20	128.8	0.26	0.13	0.40	0.49
1.20	1.40	119.6	0.26	0.11	0.42	0.50
1.40	1.60	107.6	0.28	0.16	0.41	0.52
1.60	1.80	89.9	0.30	0.13	0.46	0.57
1.80	2.00	68.7	0.34	0.17	0.49	0.62
2.00	2.20	45.39	0.45	0.27	0.44	0.69
2.20	2.40	22.43	0.78	0.43	0.52	1.03

**Table 29:** Differential cross section for the $Z/\gamma ^* \rightarrow \mu ^+\mu ^-$ process in the region $46<m_{\ell \ell }<66\,\,\text {GeV}$ (a) and $116<m_{\ell \ell }<150\,\,\text {GeV}$ (b), extrapolated to the common fiducial region. The relative statistical ($\delta _\mathrm {sta}$), uncorrelated systematic ($\delta _\mathrm {unc}$), correlated systematic ($\delta _\mathrm {sys}$), and total ($\delta _\mathrm {tot}$) uncertainties are given in percent. The overall $1.8$% luminosity uncertainty is not included

$|y_{\ell \ell }|^\mathrm {min}$	$|y_{\ell \ell }|^\mathrm {max}$	$\mathrm {d}\sigma /\mathrm {d}|y_{\ell \ell }|$ (pb)	$\delta _\mathrm {sta}$ (%)	$\delta _\mathrm {unc}$ (%)	$\delta _\mathrm {sys}$ (%)	$\delta _\mathrm {tot}$ (%)
(a)						
0.00	0.40	3.444	1.3	0.6	1.6	2.2
0.40	0.80	3.479	1.2	0.6	1.5	2.0
0.80	1.20	3.375	1.2	0.6	1.5	2.0
1.20	1.60	3.412	1.2	0.5	1.4	1.9
1.60	2.00	2.914	1.3	0.5	1.4	1.9
2.00	2.40	1.522	2.0	0.7	1.5	2.6
(b)						
0.00	0.40	1.505	1.8	0.8	1.4	2.4
0.40	0.80	1.467	1.8	0.8	1.4	2.4
0.80	1.20	1.356	1.9	0.9	1.3	2.5
1.20	1.60	1.172	1.9	0.8	1.3	2.5
1.60	2.00	0.766	2.5	0.9	1.7	3.2
2.00	2.40	0.324	4.2	1.5	1.9	4.8

## Footnotes

1 ATLAS uses a right-handed coordinate system with its origin at the nominal interaction point (IP) in the centre of the detector and the *z*-axis along the beam pipe. The *x*-axis points from the IP to the centre of the LHC ring, and the *y*-axis points upward. Cylindrical coordinates $(r,\phi )$ are used in the transverse plane, $\phi$ being the azimuthal angle around the *z*-axis. The pseudorapidity is defined in terms of the polar angle $\theta$ as $\eta =-\ln \tan (\theta /2)$. The distance in $\eta$–$\phi$ space between two objects is defined as $\Delta R = \sqrt{(\Delta \eta )^2 + (\Delta \phi )^2}$. The rapidity is defined as $y=\frac{1}{2}\ln \frac{E+p_z}{E-p_z}$.

2 Using the default parameters of this program, with an intrinsic xqtcut parameter chosen to be 0.008, the fiducial NNLO QCD predictions are found to behave in a continuous way with respect to small variations in the minimum lepton $p_{\text {T}}$ requirements around the choice of equal threshold values chosen for all fiducial regions of this paper.

3 The FEWZ and DYNNLO programs differ in the subtraction schemes used, which leads to small differences in the boson $p_{\text {T}}$ distributions at low values. This effect on the fiducial cross-section predictions is significant compared to the present experimental precision. Further efforts will be needed to understand this effect and the role of boson $p_{\text {T}}$ in fiducial cross-section predictions and to reduce the impact on the extracted PDFs.

4 Combined higher-order $\alpha \cdot \alpha _{\text {S}}$ corrections to resonant *W*,  *Z* production were recently considered in Ref. [105]. Another approach to combine NLO QCD and NLO EW effects, using the Powheg method, has been presented in Refs. [106–108].

5 This implies that the projections onto the axes correspond to 1.52 times the one-dimensional uncertainty. This is the same convention as chosen in Refs. [1, 7]. However, in the literature one may find an alternative definition, where the size of ellipses reflect the one-dimensional uncertainties when projected on the axes [116].

6 The $\chi ^2$ for the CT10 NNLO PDF set [62] is similar to that of CT14.

7 The choice of the heavy-flavour scheme is especially relevant for the HERA measurements at lower $Q^2$, see Ref. [32].

8 An exception is the introduction of a negative term in the gluon parameterization, $-A'_g x^{B'_g} (1-x)^{C'_g}$, for which two parameters, $A'_g$ and $B'_g$, are introduced simultaneously. As in Ref. [32], the parameter $C'_g$ is fixed to a large value, chosen to be $C'_g=25 \gg C_g$ to suppress the contribution at large *x*.

9 The value of Bjorken $x=0.023$ at $Q^2_0$ roughly corresponds to the region of maximum sensitivity of a measurement at central rapidity at $\sqrt{s}=7\,\text {TeV}$ and a scale of $Q^2=m_Z^2$ [38].

10 The CT10nnlo PDF set [62] is observed to have a less suppressed strange-quark distribution with $R_s = 0.80^{+0.20}_{-0.16}$ and $r_s=0.76^{+0.19}_{-0.16}$, which is in slightly better agreement with the data than the newer CT14 PDF set.
